# Carbonic Anhydrase Inhibition Activities of Schiff’s Bases Based on Quinazoline-Linked Benzenesulfonamide

**DOI:** 10.3390/molecules27227703

**Published:** 2022-11-09

**Authors:** Adel S. El-Azab, Alaa A.-M. Abdel-Aziz, Hazem A. Ghabbour, Silvia Bua, Alessio Nocentini, Hamad M. Alkahtani, Nawaf A. Alsaif, Mohamed H. M. Al-Agamy, Claudiu T. Supuran

**Affiliations:** 1 Department of Pharmaceutical Chemistry, College of Pharmacy, King Saud University, P.O. Box 2457, Riyadh 11451, Saudi Arabia; 2 Department of Medicinal Chemistry, Faculty of Pharmacy, Mansoura University, Mansoura 35516, Egypt; 3 Department of Neurofarba, Sezione di Scienze Farmaceutiche Nutraceutiche, Università degli Studi di Firenze, Via U. Schiff 6, 50019 Sesto Fiorentino, Florence, Italy; 4 Department of Pharmaceutics and Microbiology, College of Pharmacy, King Saud University, P.O. Box 2457, Riyadh 11451, Saudi Arabia

**Keywords:** quinazolinones, sulfonamide, schiff’s bases, selectivity CA inhibitors

## Abstract

Human carbonic anhydrase (CA, EC 4.2.1.1) (hCA) isoforms I, II, IX, and XII were investigated for their inhibitory activity with a series of new Schiff’s bases based on quinazoline scaffold **4**–**27**. The hCA I isoform was efficiently inhibited by Schiff’s bases **4**–**6**, **10**–**19**, **22**–**27** and had an inhibition constant (Ki) value of 52.8–991.7 nM compared with AAZ (Ki, 250 nM). Amongst the quinazoline derivatives, the compounds **2**, **3**, **4**, **10**, **11**, **16**, **18**, **24**, **26**, and **27** were proven to be effective hCA II inhibitors, with Ki values of 10.8–52.6 nM, measuring up to AAZ (Ki, 12 nM). Compounds **2**–**27** revealed compelling hCA IX inhibitory interest with Ki values of 10.5–99.6 nM, rivaling AAZ (Ki, 25.0 nM). Quinazoline derivatives **3**, **10**, **11**, **13**, **15**–**19**, and **24** possessed potent hCA XII inhibitory activities with K_I_ values of 5.4–25.5 nM vs. 5.7 nM of AAZ. Schiff’s bases **7**, **8**, **9**, and **21** represented attractive antitumor hCA IX carbonic anhydrase inhibitors (CAIs) with K_I_ rates (22.0, 34.8, 49.2, and 45.3 nM, respectively). Compounds **5**, **7**, **8**, **9**, **14**, **18**, **19**, and **21** showed hCA I inhibitors on hCA IX with a selectivity index of 22.46–107, while derivatives **12**, **14**, and **18** showed selective hCA I inhibitors on hCA XII with a selectivity profile of 45.04–58.58, in contrast to AAZ (SI, 10.0 and 43.86). Compounds **2**, **5**, **7**–**14**, **19**–**23**, and **25** showed a selectivity profile for hCA II inhibitors over hCA IX with a selectivity index of 2.02–19.67, whereas derivatives **5**, **7**, **8**, **13**, **14**, **15**, **17**, **20**, **21**, and **22** showed selective hCA II inhibitors on hCA XII with a selectivity profile of 4.84–26.60 balanced to AAZ (SI, 0.48 and 2.10).

## 1. Introduction

**Figure 1:**
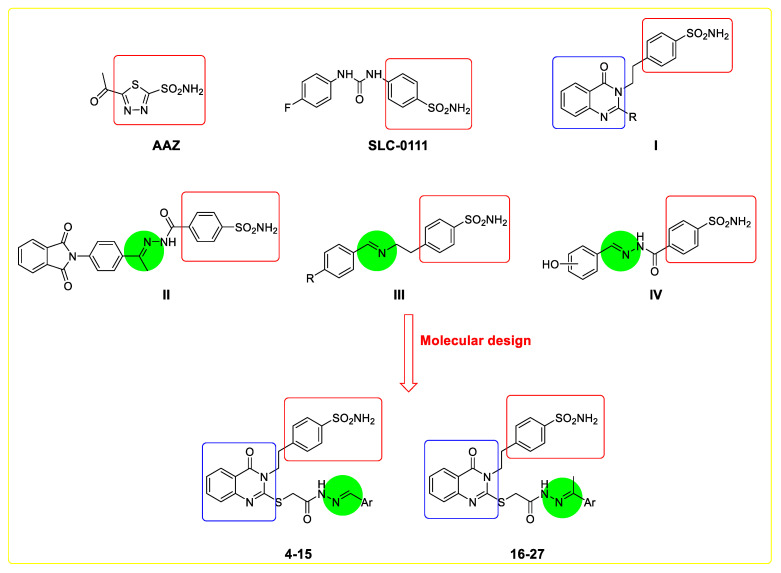
Structures of **AAZ**, **SLC**-**0111**, **I**-**IV**, and the designed Schiff’s bases incorporating quinazoline scaffold (**4**–**27**) as carbonic anhydrase inhibitors (CAIs).

Carbonic anhydrase is a zinc-metalloenzyme (CA, EC 4.2.1.1). In vertebrates, the α-carbonic anhydrases are present in different tissues and differ in function and kinetic patterns [1,2]. Functional impairment of different CA isoforms may lead to several human diseases [1]. CA I and CA II control the acid-base equilibrium throughout the physiological pathways used for cerebral edema, glaucoma, and epilepsy treatment [3]. CA IX and CA XII have been verified in plain tissues. Furthermore, CA IX and CA XII isotypes boost several cancer cells, such as breast, urinary bladder, and lung [4]. Metamorphosis of CO_2_ to HCO_3_^−^ and a proton is achieved via CA IX and CA XII. It simplifies the diffusion of the protons inside tumor cells, directing a decrease in extracellular pH and accelerating matrix decomposition, invasion, and drug resistance [1,5]. Significant architectural similarities in CA isoforms necessitate the production of more small compounds with high CA isoform selectivity to treat certain diseases without side effects, which is critical in medicinal chemistry [6]. Sulfonamide derivatives are a preferred class of COX-2 inhibitors and antitumor agents [7,8,9,10,11]. In addition, compounds incorporating sulfonamide fragments are highly used as CA inhibitors [10,11,12,13,14,15,16,17,18,19,20,21,22,23,24,25]. Furthermore, SLC-0111 (Figure 1) is a sensitive inhibitor of CA IX/XII with a sulfonamide moiety (Phase I) for the rehabilitation of solid metastatic tumors via zinc metalloenzyme fixation [12,13,26,27,28,29]. In pharmaceutical chemistry, the quinazolinone scaffold is also commonly employed [30,31,32,33,34,35,36,37,38,39,40,41,42,43,44,45,46,47,48,49,50,51,52,53,54]. Interestingly, a family of mercaptoquinazolines (**I**) could be exploited to develop new efficacious and sensitive CAIs countering various disorders and antitumor activities [32,40,44,45,55,56]. Indeed, Schiff’s bases containing benzenesulfonamide moieties, such as compounds **II**–**IV** (Figure 1), displayed versatile inhibition against CAs [13,57,58]. According to the logic mentioned above, this work focused on using quinazolinone scaffold as hCA isoforms inhibitor (C, Figure 1), incorporating ethylbenzenesulfonamide moiety [55,59,60], for (i) the synthesis of novel ester, acid-hydrazide, and Schiff’s bases with activating and deactivating groups; (ii) evaluation results for these new molecules’ CA inhibitory action on the isoenzymes I, II, IX, and XII; (iii) the structure–activity interactions of these Schiff’s bases together with various substituents and the inhibition of CA isoforms I, II, IX, and XII.

## 2. Results and Discussion

### 2.1. Chemistry

**Scheme 1:**
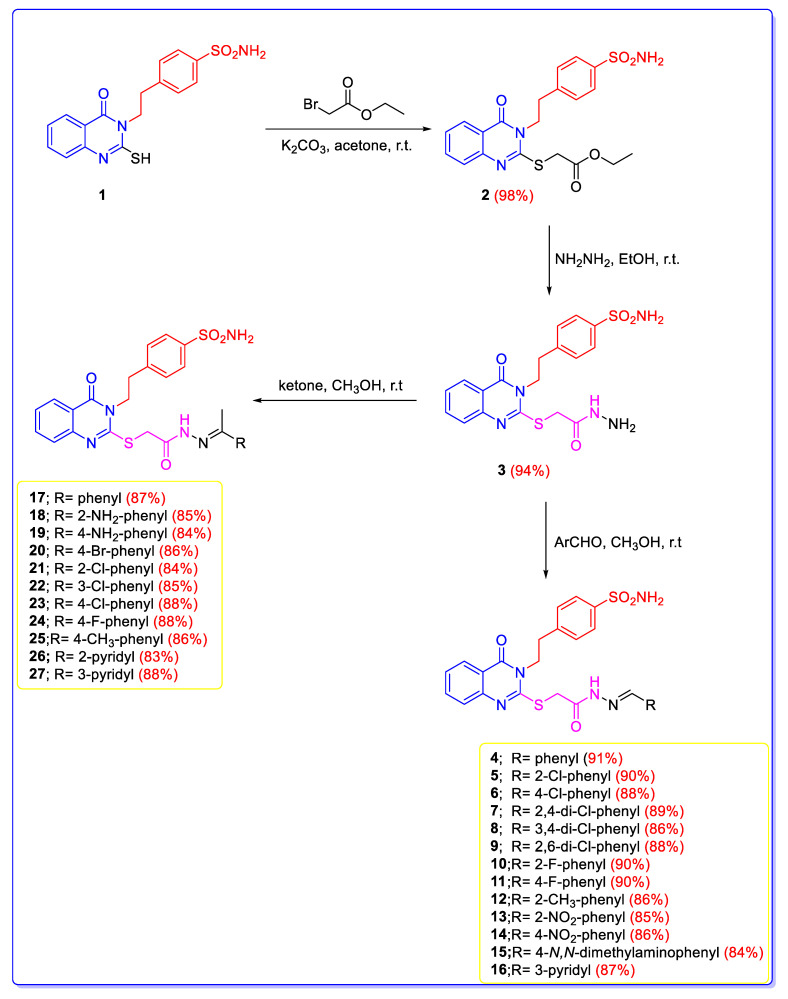
Synthesis of the designed quinazoline derivatives (**2**–**27**).

The synthetic pathway of the novel series of 2-mercapto-quinazolines-incorporating benzylidene thioacetohydrazide and phenylethylidene thioacetohydrazide derivatives is shown in Scheme 1. The stirring of 4-(2-(2-mercapto-4-oxoquinazolin-3(4H)-yl)ethyl)benzenesulfonamide (**1**) with ethyl 2-bromoacetate in acetone and potassium carbonate afforded the corresponding 2-ethyl-thioacetate ester **2** in 98% yield. The ester **2** was assessed by thiomethyl peaks (SCH_2_-COOCH_2_CH_3_) at 4.16 and 33.61 ppm and ethyl ester peaks (SCH_2_-COOCH_2_CH_3_) at 4.28, 1.22, 61.62, and 14.62 ppm in ^1^H NMR and ^13^C NMR, respectively, as well as the carbonyl peak (SCH_2_-COOCH_2_CH_3_) at 168.73 ppm in ^13^C NMR. The acid hydrazide **3** was obtained in 94% yield by stirring 2-ethyl-thioacetate ester **2** with hydrazine hydrate in ethanol. ^1^H NMR and ^13^C NMR of compound **3** revealed thiomethyl peaks (SCH_2_CONHNH_2_) at 4.02 and 33.60 ppm, respectively. The hydrazide group of thioacetohydrazide moiety (SCH_2_CONHNH_2_) was assessed by peaks at 9.42 and 4.37 ppm in ^1^H NMR, together with the carbonyl peak (SCH_2_CONHNH_2_) at 166.36 ppm in ^13^C NMR. Derivative **3** was stirred with an aldehyde or acetophenone derivative in methanol containing acetic acid-produced Schiff’s bases **4**–**27** (E and Z mixtures) in 83–91% yield. Schiff’s bases **4**–**27** were recognized via the disappearance of the singlet peak of the NH_2_ group at 4.37 ppm and the presence of substituted benzylidene fragment (-SCH2CONHN = CH-R) or phenylethylidene fragment (-SCH_2_CONHN = C(CH_3_)-R) peaks. Benzylidene fragment of Schiff’s bases **4**–**16** was identified by a singlet mercaptomethyl peak (SCH_2_CONHN = CH-R) at 4.75–4.14 ppm in ^1^H NMR and 33.64–33.59 ppm in ^13^C NMR. It was further recognized by aminocarbonyl proton (SCH_2_CONHN = CH-R) at 12.25–11.39 ppm and benzylidene proton (SCH_2_CONHN = CH-R) at 9.68–7.99 in ^1^H NMR. ^13^C NMR confirmed the carbonyl and imino-carbon groups (SCH_2_CONHN = CH-R) at 169.60–167.78 and 156.56–156.10 ppm, respectively in ^13^C NMR. A singlet mercaptomethyl peak assigned phenylethylidene moiety of Schiff’s bases **17**–**27** (SCH_2_CONHN = C(CH_3_)-R) at 4.74–4.22 ppm in ^1^H NMR and 33.64–33.61 ppm in ^13^C NMR. Additionally, it was further identified by aminocarbonyl peaks (SCH_2_CONHN = C(CH_3_)-R) at 11.11–10.71 ppm in ^1^H NMR, as well as methyl peaks (SCH_2_CONHN = C(CH_3_)-R) at 2.46–2.21 ppm in ^1^H NMR and 18.64–12.49 in ^13^C NMR, respectively. ^13^C NMR confirmed the presence of carbonyl (CO) and imino-carbon groups of phenylethylidene moiety (SCH_2_CONHN = C(CH_3_)-R) at 170.15–169.92 and 156.48–156.11 ppm, respectively. Additionally, compounds **2**–**27** were characterized by three peaks at 4.64–4.12, 3.13–3.06, and 7.38–7.35 ppm for ethylbenzenesulfonamide moieties (-CH_2_CH_2_-Ph-SO_2_NH_2_) in ^1^H NMR, together with typical peaks at 47.05–45.47 and 45.53–35.94 ppm in ^13^C NMR spectra and carbonyl group (C = O) at 160.89–160.79 ppm representing quinazoline nucleus.

### 2.2. CA Inhibitory Activity

Newly prepared quinazolines **2**–**27** were assayed against CAI activity isoforms, such as hCA I, II, IX, and XII compared with the standard sulfonamide inhibitor acetazolamide (AAZ). The tested compounds showed a selectivity index (SI) of hCA I and hCA II/hCA IX (SI, 0.77–107 and 1.85–85.58, respectively), related to the AAZ selectivity index (SI, 10.0 and 43.86). The selectivity index of hCA I and hCA II/hCA XII of the tested compounds exhibited (SI, 0.32–19.67 and 0.31–26.60, respectively, allied to AAZ (SI, 0.48 and 2.10). Compounds **2**, **5**, **7**, **8**, **9**, **10**, **12**, **13, 14**, **18**, **19**, **20**, **21**, **22**, and **25** showed selective solid hCA I inhibitory activity/hCA IX (SI, 10.16–107), while compounds **1**, **9**, **13**, **15**, and **18** displayed great selective hCA I inhibitory activity/hCA XII (SI, 40.35–85.58) associated with AAZ (SI, 10.0 and 43.86), respectively. Compounds **2** and **4**–**25** showed effective selective hCA II inhibitory activity/hCA IX (SI, 0.63–19.67), whereas compounds **3**, **5**, **7**, **8**, **9**, **11**, **13**, **14**, **15**, **17**, **19**, **20**, **21**, **22**, **23**, and **25** exhibited unusual selective hCA II inhibitory activity over hCA XII (SI, 2.19–26.60), compared with AAZ (SI, 0.48 and 2.10), respectively.

#### 2.2.1. CA I Inhibitory Activity

The CAI activity toward distinct hCA isoforms, hCA I, was efficiently inhibited by quinazolines **2**, **3**, **4**, **6**, **10**, **11**, **12**, **14**, **15**, **16**, **17**, **19**, **23**, **24**, **25**, **26**, and **27** (Ki, 52.8–635.4 nM), equated to (AAZ: Ki, 250.0 nM). Compounds **5**, **13**, **18**, and **22** showed moderate hCA I inhibitory action (Ki, 824.3–991.7 nM). In contrast, derivatives **7**, **8**, **9**, **20**, and **21** had a feeble inhibitory action (Ki, 1145–2354 nM). Structure–activity interactions of hCA I activities with Ki values indicated the following: (i) Ester **2** and acid-hydrazide **3** showed strong hCA I activities (Ki, 106.7 and 87.6 nM, respectively); (ii) conversion of acid-hydrazide **3** into corresponding Schiff’s bases **4***–***27** gave different hCA I activities (Ki, 52.8–2354 nM); (iii) unsubstituted-2-benzylidene derivatives, such as **4** (Ki, 152.4 nM) are more influential than different substituted chloro-2-benzylidenes, such as hydrazones **5**–**9** with (Ki, 567.6–2345 nM); (iv) 4-chloro-2-benzylidene derivative **6** (Ki, 567.6 nM) is more powerful than 2-chloro-2-benzylidene derivative **5** (Ki, 940.3 nM); (v) dichloro-2-benzylidene derivatives, such as **7**–**9** (Ki, 1827–2354 nM) are less powerful than monochloro-2-benzylidene derivatives **5**–**6** (Ki, 567.6–940.3 nM); (vi) substitution of 2-chloro or 4-chloro group of compounds **5**–**6** (Ki, 567.6–940.3 nM) by 2-fluoro or 4-fluoro group produced fluoro-2-benzylidenehydrazineyl derivatives **10**–**11** with an increase in hCA I activity (Ki, 132.9–274.1 nM); (vii) substituting the 2-chloro group of hydrazone **5** (Ki, 940.3 nM) by activating a group, such as the 2-methyl group produced compound **12** with an improvement in hCA I activity (Ki, 337.8 nM), whereas substituting a 4-chloro group of hydrazone **6** (Ki, 567.6 nM) by an activating group, such as 4-*N*,*N*-dimethyl amino group produced compound **15** while maintaining the hCA I activity (Ki, 582.0 nM); (viii) substituting the chloro group of compounds **5** and **6** (Ki, 940.3 and 567.6 nM, respectively) by the NO_2_ group produced hydrazones **13** and **14** with a decrease in hCA I activity (Ki, 991.7 and 635.4 nM, respectively); (ix) switching phenyl nucleus of hydrazone **4** (Ki, 152.4 nM) by the 3-pyridyl moiety produced compound **16** with a mild decrease in hCA I activity (Ki, 207.9 nM); (x) 1-phenylethylidenehydrazineyl **17** (Ki, 124.3 nM) is more effective than the substituted-1-phenylethylidenehydrazineyl derivatives, such as **18**–**25** (Ki, 186.9–1356 nM); (xi) replacing the phenyl nucleus of hydrazone **17** (Ki, 124.3 nM) by the 2-pyridyl moiety produced compound **26** with an increase in hCA I activity (Ki, 52.8 nM), whereas replacing the phenyl nucleus of compound **17** by 3-pyridyl moiety gave compound **27** with a decrease in hCA I activity (Ki, 238.4 nM); (xii) in comparison, 4-amino or 4-chloro-phenylethylidenehydrazineyl derivatives, such as **19** and **23** (Ki, 439.5 and 563.5 nM, respectively) are more active than the corresponding 2-amino, 2-chloro-phenylethylidenes, such as **18** and **21** (Ki, 824.3 and 1145 nM, respectively); (xiii) 4-fluoro-phenylethylidene **24** (Ki, 186.9 nM) is more active than the corresponding 4-amino, 4-bromo, 4-chloro or 4-methyl-phenylethylidenes, such as **19, 20, 23,** and **25** (Ki, 439.5–1356 nM); (xiv) in comparison, phenylethylidene **17** (Ki, 124.3 nM) is more forceful than the parallel benzylidene **4** (Ki, 152.4 nM), whereas benzylidenes **5**, **6**, and **11** (Ki, 940.3, 567.6, and 132.0 nM, respectively) are more equivalent than the comparable phenylethylidenes **21**, **23**, and **24** (Ki, 1145, 563.5, and 186.9 nM, respectively).

#### 2.2.2. CA II Inhibitory Activity

Quinazolines **2**, **3**, **10**, **11**, **16**, **18**, **24**, **26**, and **27** are potent hCA II inhibitors, with Ki of 10.80–49.5 nM, superior to or nearly equally active to AAZ (Ki, 12.0 nM). Compounds **4**, **12**, **15**, **17**, and **19** showed moderate hCA II inhibitory activity with (Ki, 52.6 and 82.1 nM), whereas hydrazonoquinazolines **5**, **6**, **7**, **8**, **9**, **13**, **14**, **20**, **21**, **22**, **23**, and **25** showed a weedy inhibitory action with Ki in the range of 126.0–698.2 nM. Structure–activity interactions of hCA II activities with Ki values designated the following: (i) Ester **2** and acid-hydrazide **3** showed intense hCA II activities (Ki, 16.9–21.3 nM), but the ester **2** (Ki, 21.3 nM) is less active than acid-hydrazide **3** (Ki, 16.9 nM); (ii) conversion of acid-hydrazide **3** into the corresponding hydrazones **4***–***27** gave different hCA II activities (Ki, 10.8–698.2 nM); (iii) fluoro group insertion into unsubstituted-2-benzylidene **4** (Ki, 52.6 nM) gave fluoro-2-benzylidenes **10** and **11** (Ki, 35.7–49.5 nM) with an increase in hCA II activity; (iv) substitution of fluoro group of compounds **10**–**11** (Ki, 35.7–49.5 nM) by chloro, nitro, methyl or dimethylamino groups produced substituted-2-benzylidenes **5**–**9** and **12**–**15** with a decrease in hCA II activity (Ki, 69.4–569.4 nM); (v) monochloro-2-benzylidenes **5** and **6** (Ki, 126.8–251.3 nM) are more powerful than dichloro-2-benzylidenes **7**, **8**, and **9** (Ki, 164.8–432.8 nM); (vi) in comparison, 4-chloro or 4-nitro-2-benzylidenes **6** and **14** (Ki, 126.8 and 220.7 nM, respectively) are more active than the corresponding 2-chloro or 2-nitro-2-benzylidenes **5** and **13** (Ki, 251.3 and 569.4 nM, respectively); (vii) replacing the chloro group of compounds **5** and **6** (Ki, 251.3 and 126.8 nM, respectively) by activating factions as 2-methyl or 4-*N*,*N*-dimethyl amino produced compounds **12** and **15** with an hCA II improvement in activity (Ki, 69.4 and 82.1 nM, respectively); (viii) phenyl moiety replacement of compound **4** (Ki, 52.6 nM) by the 3-pyridyl moiety produced compound **16** with a likely increase in hCA II activity (Ki, 23.8 nM); (ix) 1-phenylethylidene **17** (Ki, 67.5 nM) is more active than the substituted-1-phenylethylidene derivatives **18**–**25** (Ki, 126.0–698.2 nM) except for amino-1-phenylethylidenes **18**, **19**, and 4-fluoro-1-phenylethylidene **24** (Ki, 35.2–59.3 nM); (x) replacing the phenyl moiety of compound **17** (Ki, 67.5 nM) with the pyridyl moieties produced compounds **26** and **27** with an increase in hCA II activity (Ki, 10.8–22.8 nM); (xi) replacing the chloro group of compounds **21** and **23** (Ki, 157–324.2 nM) by activating groups, such as methyl or amino groups produced compounds **18**, **19**, and **25** with an hCA II improvement in activity (Ki, 35.2–126.0 nM); (xii) in comparison, 2-amino-phenylethylidene **18** (Ki, 35.2 nM) is more active than the corresponding 4-aminophenylethylidene **19** (Ki, 59.3 nM), whereas 2-chlorophenylethylidene **21** (Ki, 324.2 nM) is rarer than 4-chlorophenylethylidene **23** (Ki, 157.0 nM); (xiii) in comparison, benzylidenes **4**, **5**, and **6** (Ki, 52.6–251.3 nM) are forceful than the corresponding phenylethylidenes **17**, **21**, and **23** (Ki, 67.5–324.2 nM), whereas benzylidenes **11** and **16** (Ki, 23.8–49.5 nM) are lower in efficacy than the congruent phenylethylidenes **24** and **27** (Ki, 10.8–39.5 nM).

#### 2.2.3. CA IX Inhibitory Activity

Hydrazonoquinazolines **2**, **5**, **7**, **8**, **9**, **10**, **11**, **12**, **14**, **16**, **18**, **19**, **21**, **24**, **25**, and **27** displayed compelling hCA IX inhibitory effect with (Ki, 5.8–49.2 nM), being more effectual than or nearly equivalent to AAZ (Ki, 25 nM). Against hCA IX, hydrazonoquinazolines **3**, **4**, **6**, **13**, **15**, **17**, **20**, **22**, **23**, and **26** exhibited moderate inhibitory vigor, with (Ki, 52.1–99.6 nM), respectively. Dialkylated hydrazones **7**, **8**, and **9** showed selective inhibition against tumor-associated CA IX with Ki, 22–49.2 nM. At the same time, they possessed a neglected inhibitory activity toward hCA I (Ki, 1145–2354 nM), hCA II (Ki, 164.8–432.8 nM), and hCA XII (Ki, 55.9–84.4 nM) allied to AAZ (Ki, 250.0, 12.0, and 5.7, respectively nM). Compounds **7**, **8**, and **9** showed the selectivity index of hCA I inhibitory activity/hCA IX (SI, 107.0, 52.5, and 45.85) matched to AAZ (SI, 10.0), and the selectivity index of hCA II inhibitory activity/hCA IX (SI, 19.67, 9.33, and 3.35) matched to AAZ (SI, 0.48).

Structure–activity interactions of hCA IX activities with Ki values focused on the following: (i) Ester **2** and acid-hydrazide **3** showed intense hCA IX activities (Ki, 10.5 and 52.1 nM); (ii) hydrazinolysis of the ester **2** (Ki, 10.5 nM) produced acid-hydrazide **3** (Ki, 52.1 nM) with depression of hCA IX inhibitory vigor; (iii) conversion of acid-hydrazide **3** into corresponding hydrazones **4***–***27** gave different hCA IX activities (Ki, 5.8–99.6 nM); (iv) conversion of acid-hydrazide **3** (Ki, 52.1 nM) into unsubstituted-2-benzylidene **4** (Ki, 61.7 nM) leads to a neglected decrease in hCA IX activity; (v) substituted-2-benzylidenes **5**–**15** (Ki, 15.4–58.5 nM) are greater in efficacy than unsubstituted-2-benzylidene **4** (Ki, 61.7 nM) except for hydrazones **6** and **15** (Ki, 88.6 and 89.1 nM, respectively); (vi) in comparison, 4-fluoro or 4-nitro-2-benzylidenes **11** and **14** (Ki, 15.4 and 26.9 nM, separately) are more active than the corresponding 2-fluoro or 2-nitro-2-benzylidenes **10** and **13** (Ki, 19.7 and 58.5 nM, respectively), whereas 2-chlor-2-benzylidene **5** (Ki, 26.4 nM) are further vigorous than 4-chlor-2-benzylidene **6** (Ki, 89.1 nM); 2,4-dichloro-2-benzylidene **7** (Ki, 22.0 nM) is more active than the congruous 3,4-dichloro and 2,6- dichlor-2-benzylidenes **8** and **9** (Ki, 34.8 and 49.2 nM, respectively); (vii) in comparison, 2-fluoro or 4-fluoro-2-benzylidenes **10** and **11** (Ki, 19.7 and 15.4 nM, respectively) are more active than the corresponding 2-chloro or 4-chloro 2-benzylidenes **5** and **6** (Ki, 26.4, 89.1 nM) and 2-nitro or 4-nitro 2-benzylidenes **13** and **14** (Ki, 58.5 and 26.9 nM, respectively); (viii) phenyl moiety replacement of compound **4** (Ki, 61.7 nM) with the 3-pyridyl moiety produced compound **16** with a likely increase in hCA IX activity (Ki, 37.8 nM); (ix) 1-phenylethylidene **17** (Ki, 55.7 nM) are low in effect than 4-fluoro-1-phenylethylidene **24** (Ki, 28.5 nM); (xi) replacing the 4-fluoro group of compound **24** (Ki, 28.5 nM) by an activating group, such as the 4-amino group produced compound **19** with an hCA IX improvement in activity (Ki, 5.8 nM), whereas replacing it with 4-bromo, 4-chloro or 4-methyl groups gave compounds **20**, **23**, and **25** with a decrease in hCA IX activity (Ki, 43.9–99.6 nM); (xii) in comparison, 4-amino-1-phenylethylidene **19** (Ki, 5.8 nM) is more active than the corresponding 2-amino-1-phenylethylidene **18** (Ki, 36.7 nM), whereas 4-chloro-1-phenylethylidene **23** (Ki, 65 nM) is less active than 2-chloro-1-phenylethylidene **21** (Ki, 45.3 nM); (xiii) replacing the phenyl nucleus of compound **17** (Ki, 55.7 nM) by the 2-pyridyl moiety produced hydrazone **26** with a mild decrease in hCA IX activity (Ki, 68.2 nM), whereas replacing it with 3-pyridyl grew compound **27** (Ki, 33.7 nM) with a good increase in hCA IX activity; (xiv) replacing the chloro group of compounds **21** and **23** (Ki, 45.3–65.0 nM) by activating units, such as amino or methyl groups produced compounds **18**, **19**, and **25** with an hCA IX improvement in activity (Ki, 5.8–43.9 nM); (xv) in comparison, benzylidenes **5** and **11** (Ki, 26.4 and 15.4 nM) are further stronger than the analogous phenylethylidenes **21** and **24** (Ki, 45.3 and 28.5 nM, respectively), whereas phenylethylidenes **17**, **23**, and **27** (Ki, 55.7, 65.0, and 33.7 nM, respectively) are more powerful than the equivalent benzylidenes **4**, **6** and **16** (Ki, 61.7, 89.1, and 37.8 nM, respectively).

#### 2.2.4. CA XII Inhibitory Activity

Derivatives **3**, **11**, **15**, **17**, **18**, and **19** exerted intense hCA XII suppressant activities with (Ki, 5.4–18.3 nM) matched to AAZ (Ki, 5.7 nM). Compounds **2**, **4**, **10**, **13**, **14**, **16**, **24**, **26**, and **27** had moderate hCA XII inhibitory vigor with (Ki, 20.80–38.4 nM), whereas hydrazones **5**, **6**, **7**, **8**, **9**, **12**, **20**, **21**, **22**, **23**, and **25** showed soft hCA XII inhibitory action (Ki, 46.1–89.4 nM, Table 1). Structure–activity interactions of hCA XII activities with Ki values directed the following: (i) Ester **2** showed moderate hCA XII activity (Ki, 34.7 nM); (ii) hydrazinolysis of the ester **2** (Ki, 34.7 nM) produced acid-hydrazide **3** (Ki, 5.4 nM) with an increase in the hCA XII suppressant effect; (iii) conversion of acid-hydrazide **3** into corresponding hydrazones **4***–***27** gave different hCA XII activities (Ki, 5.4–89.4 nM); (iv) conversion of acid-hydrazide **3** (Ki, 5.4 nM) into unsubstituted-2-benzylidene **4** (Ki, 38.4 nM) decreased hCA XII activity; (v) insertion of fluoro or 4-N, N-dimethylamino groups into the unsubstituted-2-benzylidene **4** (Ki, 38.4 nM) gave compounds **10**, **11,** and **15** (Ki, 22.9, 15.8, and 6.8 nM, separately) with an increase in hCA XII activity, whereas insertion of chloro or methyl groups **5**–**9** and **12** (Ki, 46.1–89.4 nM) decreased the hCA XII activities; (vi) in comparison, 2-chloro or 2-nitro-2-benzylidenes **5** and **13** (Ki, 46.1 and 21.4 nM, respectively) are more active than the corresponding 4-chloro or 4-nitro-2-benzylidenes **6** and **14** (Ki, 67.8 and 30.5 nM, respectively). Meanwhile, 2-fluoro-2-benzylidene **10** (Ki, 22.9 nM) is lower in activity than 4-fluoro-2-benzylidene **11** (Ki, 15.8 nM), whereas 2,6-dichloro-2-benzylidene **9** (Ki, 55.9 nM) is more powerful than the analogous 2,4-dichloride and 3,4-dichlor-2-benzylidenes **7** and **8** (Ki, 89.4 and 63.4 nM, respectively); (vii) in comparison, fluoro-2-benzylidenes **10** and **11** (Ki, 22.9 and 15.8 nM, respectively) are more active than the corresponding chloro-2-benzylidenes **5**, **6**, and 4-nitro-2-benzylidene **14** (Ki, 46.1, 67.8, and 30.5 nM, respectively); (viii) phenyl moiety replacement of compound **4** (Ki, 38.4 nM) by the 3-pyridyl moiety produced compound **16** with an increasing hCA XII activity (Ki, 20.8 nM); (ix) 1-phenylethylidene **17** (Ki, 12.7 nM) is more forceful than benzylidene **4** (Ki, 38.4 nM) and insertion of an amino or fluoro group into 1-phenylethylidene **17** (Ki, 12.7 nM) gave compounds **18**, **19**, and **24** (Ki, 18.3, 15.9, and 25.4 nM, respectively) with a neglected moderate decrease in hCA XII activity; (x) insertion of chloro or methyl groups of 1-phenylethylidene **17** (Ki, 12.7 nM) produced compounds **21**, **22**, **23**, and **25** (Ki, 58.0, 46.9, 61.2, and 57.5 nM, respectively) with reduction in hCA XII activity, whereas insertion of bromo group produced compound **20** (Ki, 88.8 nM) with a substantial decrease in hCA XII activity; (xi) in comparison, 4-amino-1-phenylethylidene **19** (Ki, 15.9 nM) is significantly forceful than the analogous 2-amino-1-phenylethylidene **18** (Ki, 18.3 nM), whereas 4-chloro-1-phenylethylidene **23** (Ki, 61.2 nM) is lower in effectivity than 2-chloro-1-phenylethylidene **21** (Ki, 58.0 nM); (xii) replacing the phenyl nucleus of hydrazone **17** (Ki, 12.7 nM) by the pyridyl moiety produced compounds **26** and **27** with a mild decrease in hCA XII activity (Ki, 28.5 and 34.7 nM, respectively); (xii) in comparison, benzylidenes **5**, **11**, and **16** (Ki, 46.1, 15.8, and 20.8 nM, respectively) are higher in power than the analogous phenylethylidenes **21**, **24**, and **27** (Ki, 58.0, 25.4, and 34.7 nM, respectively). Phenylethylidenes **17** and **23** (Ki, 12.7 and 61.2 nM, respectively) are extra forceful than the analogous benzylidenes **4** and **6** (Ki, 38.4 and 67.8 nM, respectively).

## 3. Conclusions

Novel synthesized quinazolines **2**–**27** were evaluated for their activity against CAI isoforms (hCA I, II, IX, and XII) along with acetazolamide (AAZ). The CAI activity toward different hCA isoforms, hCA I, was powerfully inhibited by derivatives **2**, **3**, **4**, **6**, **10**, **11**, **12**, **14**, **15**, **16**, **17**, **19**, **23**, **24**, **25**, **26**, and **27** (Ki, 52.8–635.4 nM) matched to (AAZ: Ki, 250.0 nM). Compounds **2**, **3**, **10**, **11**, **16**, **18**, **24**, **26**, and **27** are efficacious hCA II inhibitors, (Ki, 10.80–49.5 nM) related to AAZ (Ki, 12.0 nM). On the other hand, Schiff’s bases **4**, **12**, **15**, **17**, and **19** showed a moderate hCA II inhibitory vigor (Ki, 52.6–82.1 nM). Quinazolines **2**, **5**, **7**, **8**, **9**, **10**, **11**, **12**, **14**, **16**, **18**, **19**, **21**, **24**, **25**, and **27** revealed forceful hCA IX suppressant activity (Ki, 5.8–49.2 nM) equated to AAZ (Ki, 25 nM); however, quinazolines **3**, **4**, **6**, **13**, **15**, **17**, **20**, **22**, **23**, and **26** demonstrated equitable hCA IX suppressant activity (Ki, 52.1–99.6 nM). Compounds **3**, **11**, **15**, **17**, **18**, and **19** exerted intense hCA XII inhibitory vigor (Ki, 5.4–18.3 nM) rivaled to AAZ (Ki, 5.7 nM), while compounds **2**, **4**, **10**, **13**, **14**, **16**, **24**, **26**, and **27** had middling hCA XII suppressant activity (Ki, 20.80–38.4 nM). Quinazolines **2**, **5**, **7**, **8**, **9**, **10**, **12**, **13**, **14**, **18**, **19**, **20**, **21**, **22**, and **25** showed selectively fixed hCA I inhibitory activity over hCA IX (SI, 10.16–107.0) paralleled to AAZ (SI, 10.0). Derivatives **1**, **9**, **13**, **15**, and **18** displayed distinguished selective hCA I inhibitory vigor/hCA XII (SI, 40.35–85.58) matched to AAZ (SI, 43.86). Quinazolines **2** and **4**–**25** indicated real selective hCA II inhibitory activity/hCA IX (SI, 0.63–19.67) associated with AAZ (SI, 0.48). Quinazolines **3**, **5**, **7**, **8**, **9**, **11**, **13**, **14**, **15**, **17**, **19**, **20**, **21**, **22**, **23**, and **25** presented notable selective hCA II suppressant activity/hCA XII (SI, 2.19–26.60) allied to AAZ (SI, 2.10). Compounds **7**, **8**, and **9** showed the selectivity index of hCA I inhibitory activity over hCA IX (SI, 107.0, 52.5, and 45.85) related to AAZ (SI, 10.0) and the selectivity index of hCA II inhibitory activity over hCA IX (SI, 19.67, 9.33, and 3.35) compared with AAZ (SI, 0.48).

## 4. Materials and Methods

### 4.1. Chemistry

Stirring of anthranilic acid with 4-(2-isothiocyanatoethyl)benzenesulfonamide in ethanol containing trimethylamine gave 4-(2-(2-mercapto-4-oxoquinazolin-3(4H)-yl)ethyl)benzenesulfonamide (**1**) [55,59]. Melting points (uncorrected) were recorded on a Barnstead 9100 Electrothermal melting apparatus (APS Water Services Corporation, Van Nuys, CA, USA). In contrast, the KBr disc IR spectra are recorded on an FT-IR Perkin-Elmer spectrometer (PerkinElmer Inc., Waltham, MA, USA). The ^1^H NMR and ^13^C NMR were measured in DMSO-*d_6_* on Bruker 700 and 176 MHz instruments, respectively (Bruker, Billerica, MA, USA). Supporting Information: ^1^H NMR and ^13^C NMR of compounds **2**–**27**. Mass spectra were recorded on an Agilent 6320 Ion Trap mass spectrometer (Agilent Technologies, Santa Clara, CA, USA). C, H, and N were analyzed at the Research Centre, College of Pharmacy, King Saud University, Saudi Arabia. The results were within ±0.4% of the theoretical values.

#### 4.1.1. Ethyl 2-((4-oxo-3-(4-sulfamoylphenethyl)-3,4-dihydroquinazolin-2-yl)thio)acetate (**2**)

A mixture of 4-(2-(2-mercapto-4-oxoquinazolin-3(4H)-yl)ethyl)benzenesulfonamide (**1**) (20 mmol, 7.22 gm), ethyl 2-bromoacetate (22 mmol, 3.68 gm), and anhydrous potassium carbonate (22 mmol, 3.04 gm) was stirred at room temperature in (150 mL) acetone for 24 h. The reaction mixture was filtered, dried, washed with (10 mL) of water, and dried. Mp 210–211°, 98% yield; IR (KBr, cm^−1^) *ν*: 3308, 3217 (NH), 1735, 1661 (C = O), 1334, 1157 (O = S = O); ^1^H NMR (700 MHz, DMSO-*d_6_*): δ 8.08 (d, 1H, *J* = 7.24 Hz), 7.80 (d, 3H, *J* = 6.18 Hz), 7.49 (d, 3H, *J* = 7.20 Hz), 7.44 (d, 1H, *J* = 9.0 Hz), 7.35 (s, 2H), 4.28 (s, 2H), 4.16 (s, 4H), 3.10 (s, 2H), 1.22 (s, 3H); ^13^C NMR (175 MHz, DMSO-*d_6_*): δ 168.73, 160.76, 155.92, 147.00, 143.11, 142.24, 135.36, 129.65, 126.94, 126.67, 126.49, 126.23, 119.17, 61.62, 45.54, 34.56, 33.61, 14.62; Ms: [*m/z*, 447].

#### 4.1.2. 4-(2-(2-((2-Hydrazineyl-2-oxoethyl)thio)-4-oxoquinazolin-3(4H)-yl)ethyl)benzenesulfonamide (**3**)

A mixture of hydrazine hydrate (32 mmol, 1.0 gm) and ethyl 2-((4-oxo-3-(4-sulfamoylphenethyl)-3,4-dihydroquinazolin-2-yl)thio)acetate (**2**) (15 mmol, 6.71 gm) in absolute ethanol (70 mL) was stirred for 24 h at room temperature. The reaction mixture was filtered, dried, and washed with (20 mL) of 70% ethanol. Mp 253–254°, 94% yield; IR (KBr, cm^−1^) *ν*: 3536, 3378, 3291, 3142 (NH), 1760, 1687 (C = O), 1335, 1211 (O = S = O); ^1^H NMR (700 MHz, DMSO-*d_6_*): δ 9.42 (s, 1H), 8.08 (d, 1H, *J* = 7.75 Hz), 7.81 (d, 3H, *J* = 7.79 Hz), 7.57 (d, 1H, *J* = 7.84 Hz), 7.51 (d, 2H, *J* = 7.49 Hz), 7.47 (t, 1H, *J* = 15.19 Hz), 7.36 (s, 2H), 4.37 (s, 2H), 4.28 (t, 2H, *J* = 15.51 Hz), 4.02 (s, 2H), 3.10 (t, 2H, *J* = 15.51 Hz); ^13^C NMR (175 MHz, DMSO-*d_6_*): δ 166.63. 160.88, 156.11, 147.12, 143.07, 142.36, 135.23, 129.65, 126.86, 126.56, 126.50, 119.20, 45.46, 34.46, 33.60; Ms: [*m/z*, 443].

#### 4.1.3. Synthesis of Compounds **4**–**27**

A mixture of 4-(2-(2-((2-hydrazineyl-2-oxoethyl)thio)-4-oxoquinazolin-3(4H)-yl)ethyl)benzenesulfonamide (**3**) (1 mmol, 44 mg) and appropriate aldehyde or acetophenone derivatives (1 mmol) were stirred at room temperature in absolute methanol (5 mL) and 5 drops of acetic acid for 24 h. The reaction mixture was filtered, dried, and washed with (10 mL) of 50% methanol.

##### 4-(2-(2-((2-(2-Benzylidenehydrazineyl)-2-oxoethyl)thio)-4-oxoquinazolin-3(4H)-yl)ethyl)benzenesulfonamide (**4**)

Mp 296–298°, 91% yield; IR (KBr, cm^−1^) *ν*: 3388, 3300, 3143 (NH), 1759, 1687 (C = O), 1335, 1215 (O = S = O); ^1^H NMR (700 MHz, DMSO-*d_6_*): δ 169.17, 164.02, 160.86, 160.82, 156.30, 156.19, 147.15, 147.06, 143.89, 143.08, 142.32, 135.29, 135.26, 134.58, 134.56, 130.59, 130.42, 129.67, 129.63, 129.32, 129.29, 127.57, 127.33, 126.94, 126.52, 126.50, 126.37, 126.32, 119.23, 119.18, 45.56, 45.47, 35.42, 34.27, 33.64, 33.62; ^13^C NMR (175 MHz, DMSO-*d_6_*): δ 11.88 (s, 0.36H), 11.73 (s, 0.64H), 8.29 (s, 0.36H), 8.10 (s, 0.64H), 8.08 (d, 1H, *J* = 7.49 Hz), 7.82 (d, 0.78H, *J* = 7.84 Hz), 7.79 (d, 1.72H, *J* = 7.77 Hz), 7.74 (d, 1.72H, *J* = 6.23 Hz), 7.71 (d, 0.78H, *J* = 7.00 Hz), 7.53–7.40 (m, 7H), 7.36 (s, 2H), 4.65 (s, 1.25), 4.31 (q, 2H, *J* = 8.01, 7.35, and 7.00 Hz), 4.17 (s, 0.75H), 3.12 (t, 2H, *J* = 7.0 Hz); Ms: [*m/z*, 521].

##### 4-(2-(2-((2-(2-(2-Chlorobenzylidene)hydrazineyl)-2-oxoethyl)thio)-4-oxoquinazolin-3(4H)-yl)ethyl)benzenesulfonamide (**5**)

Mp 288–290°, 90% yield; IR (KBr, cm^−1^) *ν*: 3384, 3260, 3144 (NH), 1758, 1685 (C = O), 1335, 1157 (O = S = O); ^1^H NMR (700 MHz, DMSO-*d_6_*): δ 12.12 (s, 0.35H), 11.91 (s, 0.65H), 9.69 (s, 0.35H), 8.49 (s, 0.65H), 8.08 (t, 1H, *J* = 7.21 Hz), 8.04 (d, 0.65H, *J* = 7.77 Hz), 7.94 (dd, 0.35H, *J* = 7.70 Hz), 7.82–7.75 (m, 3H), 7.55–7.7.49 (m, 3H), 7.45 (t, 2H, *J* = 14.63 Hz), 7.42–7.37 (m,2H), 7.36 (s, 2H), 4.65 (s, 1.30H), 4.31 (q, 2H, *J* = 7.91 Hz), 4.16 (s, 0.70H), 3.12 (t, 2H, *J* = 14.14 Hz); ^13^C NMR (175 MHz, DMSO-*d_6_*): δ 169.36, 164.29, 160.85, 160.81, 156.25, 156.15, 147.06, 147.04, 143.10, 143.09, 142.31, 139.94, 135.28, 133.62, 133.45, 132.05, 131.83, 131.82, 131.78, 130.46, 130.40, 129.67, 129.64, 128.14, 128.08, 127.33,, 127.26, 126.95, 126.63, 126.55, 126.52, 126.50, 126.37, 126.31, 119.23, 119.18, 45.57, 45.48, 35.41, 34.21, 33.64; Ms: [*m/z*, 555 and M + 2, 557].

##### 4-(2-(2-((2-(2-(4-Chlorobenzylidene)hydrazineyl)-2-oxoethyl)thio)-4-oxoquinazolin-3(4H)-yl)ethyl)benzenesulfonamide (**6**)

Mp 288–289°, 88% yield; ^1^H NMR (700 MHz, DMSO-*d_6_*): δ 11.94 (s, 0.40H), 11.79 (s, 0.60H), 8.28 (s, 0.38H), 8.09 (s, 0.62H), 8.07 (d, 1H, *J* = 7.42 Hz), 7.82–7.73 (m, 5H), 7.50 (t, 4H, *J* = 18.20 Hz), 7.45 (d, 1.2H, *J* = 7.28 Hz), 7.40 (d, 0.8H, *J* = 8.19 Hz), 7.36 (s, 2H), 4.64 (s, 1.2H), 4.31 (q, 2H, *J* = 7.70 Hz), 4.17 (s, 0.8H), 3.12 (t, 2H, *J* = 15.82 Hz); ^13^C NMR (175 MHz, DMSO-*d_6_*): δ 169.24, 164.14, 160.85, 160.82, 156.29, 156.17, 147.05, 145.84, 143.09, 142.60, 142.32, 135.27, 135.01, 134.82, 133.53, 129.65, 129.40, 129.21, 128.97, 126.94, 126.60, 126.52, 126.50, 126.38, 126.32, 119.23, 119.17, 45.56, 45.49, 35.4, 34.28, 33.64.

##### 4-(2-(2-((2-(2-(2,4-Dichlorobenzylidene)hydrazineyl)-2-oxoethyl)thio)-4-oxoquinazolin-3(4H)-yl)ethyl)benzenesulfonamide (**7**)

Mp 304–305°, 89% yield; IR (KBr, cm^−1^) *ν*: 3386, 3267, 3142 (NH), 1758, 1684 (C = O), 1334, 1157 (O = S = O); ^1^H NMR (700 MHz, DMSO-*d_6_*): δ 12.16 (s, 0.33H), 11.95 (s, 0.67H), 8.63 (s, 0.33H), 8.42 (s, 0.67H), 8.07 (t, 1H, *J* = 7.42 Hz), 8.03 (d, 0.67H, *J* = 8.54 Hz), 7.92 (d, 0.37H, *J* = 8.47 Hz), 7.80 (q, 2H, *J* = 8.40 Hz), 8.78–7.71 (m, 2H), 7.50 (q, 2H, *J* = 8.33 Hz),7.45 (t, 2H, *J* = 14.98 Hz), 7.39 (d, 1H, *J* = 8.12 Hz), 7.36 (s, 2H), 4.64 (s, 1.26H), 4.30 (q, 2H, *J* = 7.74 Hz), 4.16 (s, 0.74H), 3.12 (t, 2H, *J* = 15.86 Hz); ^13^C NMR (175 MHz, DMSO-*d_6_*): δ 169.38, 160.83, 160.80, 156.22, 156.10, 147.03, 143.10, 142.31, 142.07, 138.90, 135.62, 135.40, 135.27, 134.30, 134.13, 130.93, 129.90, 129.64, 128.51, 128.43, 126.93, 126.62, 126.50, 126.31, 119.16, 45.56, 45.49, 35.41, 34.26, 33.64; Ms: [*m/z*, 598 and M + 2, 591].

##### 4-(2-(2-((2-(2-(3,4-Dichlorobenzylidene)hydrazineyl)-2-oxoethyl)thio)-4-oxoquinazolin-3(4H)-yl)ethyl)benzenesulfonamide (**8**)

Mp 271–272°, 86% yield; ^1^H NMR (700 MHz, DMSO-*d_6_*): δ 12.06 (s, 0.35H), 11.89 (s, 0.65H), 8.27 (s, 0.36H), 8.07 (t, 1.64H, *J* = 6.86 and 4.14 Hz), 8.00 (s, 0.63H), 7.94 (s, 0.37H), 7.80 (t, 2.3H, *J* = 10.57 and 8.26 Hz), 7.77 (dd, 1.4H, *J* = 16.12 and 8.33 Hz), 7.70 (dd, 1.3H, *J* = 2.31 and 8.14 Hz), 7.50 (t, 2.3H, 7.49 and 7.77 Hz), 7.45 (q, 1H, *J* = 8.12 Hz), 7.39 (d, 0.7H, *J* = 8.19 Hz), 7.36 (s, 2H), 4.65 (s, 1.18H), 4.30 (q, 2H, *J* = 7.65 Hz), 4.17 (s, 0.72H), 3.12 (t, 2H, *J* = 7.70 and 7.65 Hz); ^13^C NMR (175 MHz, DMSO-*d_6_*): δ 169.39, 164.35, 160.84, 160.80, 156.26, 156.14, 147.05, 147.04, 144.46, 142.31, 141.25, 135.47, 135.43, 135.25, 132.73, 132.54, 132.23, 132.14, 131.54, 131.52, 129.66, 129.63, 129.15, 128.75, 127.24, 126.94, 126.60, 126.51, 126.50, 126.38, 126.28, 119.22, 119.17, 45.56, 45.47, 35.39, 34.33, 33.65, 33.62; Ms: [*m/z*, 589, M + 2, 591].

##### 4-(2-(2-((2-(2-(2,6-Dichlorobenzylidene)hydrazineyl)-2-oxoethyl)thio)-4-oxoquinazolin-3(4H)-yl)ethyl)benzenesulfonamide (**9**)

^1^ Mp 296–297°, 88% yield; ^1^H NMR (700 MHz, DMSO-*d_6_*): δ 12.17 (s, 0.25H), 11.99 (s, 0.75H), 8.49 (s, 0.25H), 8.35 (s, 0.75H), 8.07 (t, 1H, *J* = 7.92 Hz), 7.81 (d, 2H, *J* = 7.84 Hz), 7.74 (t, 1H, *J* = 15.06 Hz), 7.58 (d, 1.4H, *J* = 8.05 Hz), 7.56 (d, 0.60H, *J* = 8.05 Hz), 7.52 (d, 2H, *J* = 7.70 Hz), 7.47–7.43 (m, 2H), 7.39 (d, 1H, *J* = 8.12 Hz), 7.36 (s, 2H), 4.60 (s, 1.5H), 4.30 (t, 2H, *J* = 7.90 Hz), 4.15 (s, 0.50H), 3.12 (t, 2H, *J* = 15.92 Hz); ^13^C NMR (175 MHz, DMSO-*d_6_*): δ 169.57, 160.82, 156.28, 156.18, 147.06, 143.08, 142.54, 142.33, 142.30, 138.90, 135.20, 134.40, 131.56, 130.10, 129.92, 129.67, 129.63, 129.46, 126.93, 126.51, 126.24, 119.24, 119.17, 45.59, 45.49, 35.43, 34.60, 33.64.

##### 4-(2-(2-((2-(2-(2-Fluorobenzylidene)hydrazineyl)-2-oxoethyl)thio)-4-oxoquinazolin-3(4H)-yl)ethyl)benzenesulfonamide (**10**)

Mp 276–277°, 90% yield; ^1^H NMR (700 MHz, DMSO-*d_6_)*: δ 12.01 (s, 0.5H), 11.86 (s, 0.5H), 8.31–7.50 (m, 15H), 4.66 (s, 1.25H), 4.33 (s, 2.75H), 3.16 (s, 2H); ^13^C NMR (175 MHz, DMSO-*d_6_*): δ 169.30, 164.19, 161.87, 160.85, 160.45, 156.28, 147.09, 143.10, 142.33, 136.76, 135.28, 132.36, 129.66, 126.95, 126.77, 126.52, 125.42, 122.14, 119.19, 116.52, 45.49, 40.23, 40.12, 40.01, 39.89, 39.78, 34.22, 33.65; Ms: [*m/z*, 539].

##### 4-(2-(2-((2-(2-(4-Fluorobenzylidene)hydrazineyl)-2-oxoethyl)thio)-4-oxoquinazolin-3(4H)-yl)ethyl)benzenesulfonamide (**11**)

Mp 292–293°, 90% yield; ^1^H NMR (700 MHz, DMSO-*d_6_*): δ 11.89 (s, 0.38H), 11.73 (s, 0.62H), 8.29 (0.42H), 8.09 (s, 0.58H), 8.07 (s, t, 1H, *J* = 5.32 and 7.17 Hz), 7.82–7.74 (m, 5H), 7.50 (dd, 2.3H, *J* = 4.69 and 7.88 Hz), 7.45 (dd, 1H, *J* = 7.42 Hz), 7.40 (d, 0.7H, *J* = 8.12 Hz), 7.36 (s, 2H), 7.28 (a, 2H, *J* = 8.58 Hz), 4.60 (s, 1.22H), 4.31 (dd, 2H, *J* = 7.82 Hz), 4.16 (s, 0.78H), 3.12 (t, 2H, *J* = 15.86 Hz); ^13^C NMR (175 MHz, DMSO-*d_6_*): δ 169.17, 164.29, 164.16, 164.07, 162.89, 162.76, 160.87, 160.83, 156.30, 156.17, 147.07, 147.05, 146.06, 143.07, 142.78, 142.33, 135.27, 131.19, 129.79, 129.74, 129.67, 129.64, 129.52, 129.47, 126.93, 126.60, 126.51, 126.49, 126.38, 126.32, 119.22, 119.16, 116.44, 116.3, 45.55, 45.47, 35.39, 34.28, 33.638; Ms: [*m/z*, 539].

##### 4-(2-(2-((2-(2-(2-Methylbenzylidene)hydrazineyl)-2-oxoethyl)thio)-4-oxoquinazolin-3(4H)-yl)ethyl)benzenesulfonamide (**12**)

Mp 239–240°, 86% yield; ^1^H NMR (700 MHz, DMSO-*d_6_*): δ 11.86 (s, 0.39H), 11.70 (s, 0.61H), 8.25 (s, 0.35H), 8.08 (s, 0.5H), 8.07 (s, 1H), 7.82 (d, 0.75H, *J* = 7.93 Hz), 7.97 (d, 1.25H, *J* = 7.95 Hz), 7.78–7.73 (m, 1H), 7.56 (s, 0.65H), 7.53 (d, 1.5H, *J* = 8.40 Hz), 7.51 (s, 0.5H), 7.49 (d, 1.5 H, *J* = 7.92 Hz), 7.44 (q, 1H, *J* = 7.28 Hz), 7.40 (d, 1H, *J* = 8.12 Hz), 7.37 (s, 2H), 7.32 (q, 1H, *J* = 7.21 Hz), 7.24 (d, 1H, *J* = 7.42 Hz), 4.65 (1.25H), 4.30 (dd, 2H, *J* = 7.84 Hz), 4.16 (s, 0.75H), 3.12 (t, 2H, *J* = 15.84 Hz), 2.33 (d, 3H, *J* = 6.02 Hz); ^13^C NMR (175 MHz, DMSO-*d_6_*): δ 169.11, 164.00, 160.85, 160.81, 156.30, 156.17, 147.22, 147.08, 147.06, 144.04, 143.10, 143.09, 142.32, 138.57, 138.52, 135.23, 134.52, 134.51, 131.29, 131.14, 129.66, 129.62, 129.21, 129.18, 127.88, 127.65, 126.93, 126.57, 126.52, 126.50, 126.36, 126.28, 124.96, 124.70, 119.22, 119.17, 45.54, 45.45, 35.44, 34.32, 33.65, 33.62, 21.36, 21.33; Ms: [*m/z*, 535].

##### 4-(2-(2-((2-(2-(2-Nitrobenzylidene)hydrazineyl)-2-oxoethyl)thio)-4-oxoquinazolin-3(4H)-yl)ethyl)benzenesulfonamide (**13**)

Mp 288–289°, 85% yield; IR (KBr, cm^−1^) *ν*: 3392, 3287, 3142 (NH), 1755, 1682 (C = O), 1337, 1157 (O = S = O); ^1^H NMR (700 MHz, DMSO-*d_6_*): δ 12.23 (s, 0.40H), 11.98 (s, 0.60H), 8.69 (s, 0.40H), 8.46 (s, 0.60H), 8.09 (t, 1H, *J* = 8.61 Hz), 8.05 (dd, 2H, *J* = 7.49 and 7.77 Hz), 7.81–7.7.72 (m, 4H), 7.66 (q, 1H, *J* = 7.37 Hz), 7.53–7.39 (m, 4H), 7.37 (s, 2H), 4.60 (s, 1.2H), 4.30 (t, 2H, *J* = 7.35 Hz), 4.15 (s, 0.8H), 8.09 (t, 2H, *J* = 8.61 Hz); ^13^C NMR (175 MHz, DMSO-*d_6_*): δ 169.60, 164.75, 160.89, 156.18, 156.09, 148.58, 148.48, 147.01, 142.97, 142.34, 139.55, 135.26, 134.31, 133.99, 131.22, 131.04, 129.68, 129.65, 129.10, 128.73, 128.63, 128.57, 126.90, 126.66, 126.57, 126.49, 126.42, 126.30, 125.20, 125.07, 119.15, 119.10, 45.53, 45.45, 35.32, 34.16, 33.58; Ms: [*m/z*, 566].

##### 4-(2-(2-((2-(2-(4-Nitrobenzylidene)hydrazineyl)-2-oxoethyl)thio)-4-oxoquinazolin-3(4H)-yl)ethyl)benzenesulfonamide (**14**)

Mp 296–297°, 86% yield; IR (KBr, cm^−1^) *ν*: 3396, 3258, 3145 (NH), 1755, 1683 (C = O), 1340, 1157 (O = S = O); ^1^H NMR (700 MHz, DMSO-*d_6_*): δ 12.18 (s, 0.35H), 12.02 (s, 65H), 8.40 (s, 0.35H), 8.28 (t, 2H, *J* = 10.71 and 8.75 Hz), 8.20 (s, 0.65H), 8.07 (t, 1H, *J* = 5.81 and 7.07 Hz), 8.01 (dd, 2H, *J* = 8.40 Hz), 7.82 (dd, 2H, *J* = 7.88 and 7.77 Hz), 7.74 (q, 1H, *J* = 7.56 and 10.74 Hz), 7.50 (t, 2H, *J* = 8.25, 8.68 Hz), 7.45 (q, 1H, *J* = 7.28 Hz), 7.39 (d, 1H, *J* = 8.19 Hz), 7.36 (s, 2H), 4.67 (s, 1.30H), 4.31 (q, 2H, *J* = 8.33, 8.96, and 7.59 Hz), 4.20 (s, 0.70H), 3.12 (t, 2H, *J* = 15.75 Hz); ^13^C NMR (175 MHz, DMSO-*d_6_*): δ 169.61, 164.59, 160.83, 160.80, 156.23, 156.12, 148.31, 148.17, 147.05, 147.02, 144.68, 143.10, 143.09, 142.31, 141.55, 140.95, 140.86, 135.27, 129.67, 129.65, 128.49, 128.25, 126.93, 126.61, 126.54, 126.52, 126.49, 126.37, 126.32, 124.54, 124.51, 119.22, 119.17, 45.57, 45.50, 35.43, 34.25, 33.64; Ms: [*m/z*, 566].

##### 4-(2-(2-((2-(2-(4-(Dimethylamino)benzylidene)hydrazineyl)-2-oxoethyl)thio)-4-oxoquinazolin-3(4H)-yl)ethyl)benzenesulfonamide (**15**)

Mp 280–281°, 84% yield; IR (KBr, cm^−1^) *ν*: 3398, 3260, 3141 (NH), 1755, 1682 (C = O), 1338, 1157 (O = S = O); ^1^H NMR (700 MHz, DMSO-*d_6_*): δ 11.55 (s, 0.40H), 11.44 (s, 0.60H), 8.12 (s, 0.4H), 8.08 (d, 1H, *J* = 7.68 Hz), 7.95 (s, 0.60H), 7.81 (d, 1H, *J* = 7.98 Hz), 7.78 (dd, 2H, *J* = 8.12 Hz), 7.54–7.44 (m, 6H), 7.36 (d, 2H, *J* = 6.93 Hz), 6.73 (dd, 2H, *J* = 8.61 and 8.54 Hz), 4.61 (s, 1.2H), 4.30 (t, 2H, *J* = 8.12 and 7.28 Hz), 4.18 (s, 0.80H), 3.11 (t, 2H, *J* = 5.67 and 9.17 Hz), 2.96 (s, 6H); ^13^C NMR (175 MHz, DMSO-*d_6_*): δ 168.51, 163.24, 160.88, 160.83, 156.40, 156.25, 151.98, 151.83, 148.01, 147.10, 144.75, 143.09, 143.07, 142.34, 142.32, 135.26, 129.66, 129.63, 128.92, 128.61, 126.93, 126.57, 126.52, 126.50, 126.39, 126.36, 121.87, 121.75, 119.23, 119.20, 112.25, 112.22, 45.54, 45.44, 35.45, 34.40, 33.64, 33.61; Ms: [*m/z*, 564].

##### 4-(2-(4-Oxo-2-((2-oxo-2-(2-(pyridin-3-ylmethylene)hydrazineyl)ethyl)thio)quinazolin-3(4H)-yl)ethyl)benzenesulfonamide (**16**)

Mp 263–264°, 87% yield; ^1^H NMR (700 MHz, DMSO-*d_6_*): δ 12.04 (s, 0.36H), 11.89 (s, 0.46H), 8.62 (s, 1H), 8.35 (s, 0.38H), 8.17 (d, 0.64H, *J* = 7.76 Hz), 8.14 (s, 0.62H), 8.11 (d, 0.36H, *J* = 7.73 Hz), 8.07 (t, 1H, *J* = 14.07 Hz), 7.81 (dd, 2.55H, *J* = 7.94 and 7.98 Hz), 7.74 (t,0.67 H, *J* = 7.59 Hz), 7.50 (t, 3H, *J* = 9.59 and 8.47 Hz), 7.45 (dd, 2H, *J* = 8.47 Hz), 7.39 (d, 0.78H, *J* = 8.19 Hz), 7.37 (s, 2H), 4.65 (s, 1.25H), 4.31 (q, 2H, *J* = 7.94 Hz), 4.18 (s, 0.75H), 3.12 (t, 2H, *J* = 15.96 Hz); ^13^C NMR (175 MHz, DMSO-*d_6_*): δ 169.35, 164.27, 160.85, 160.82, 156.27, 156.14, 151.18, 150.99, 149.24, 148.97, 147.06, 147.04, 144.48, 143.08, 142.32, 141.13, 135.29, 135.26, 133.91, 133.82, 130.56, 129.67, 129.64, 126.93, 126.60, 126.52, 126.50, 126.39, 126.30, 124.51, 119.22, 119.16, 45.57, 45.48, 35.39, 34.27, 33.64, 33.61; Ms: [*m/z*, 522].

##### 4-(2-(4-Oxo-2-((2-oxo-2-(2-(1-phenylethylidene)hydrazineyl)ethyl)thio)quinazolin-3(4H)-yl)ethyl)benzenesulfonamide (**17**)

Mp 260–261°, 87% yield; IR (KBr, cm^−1^) *ν*: 3387, 3266, 3146 (NH), 1754, 1679 (C = O), 1339, 1156 (O = S = O); ^1^H NMR (700 MHz, DMSO-*d_6_*): δ 10.97 (s, 0.64H), 10.83 (s, 0.36H), 8.09 (dd, 1H, *J* = 7.98 Hz), 7.87 (t, 1H, *J* = 3.50 and 3.85 Hz), 7.82–7.75 (m, 3H), 7.52–7.39 (m, 8H), 7.36 (s, 2H), 4.70 (s, 1.2H), 4.31 (t, 2.8H, *J* = 8.40 and 6.79 Hz), 3.12 (t, 2H, *J* = 7.71 and 7.84 Hz), 2.34 (s, 1H), 2.32 (s, 2H); ^13^C NMR (175 MHz, DMSO-*d_6_*): δ 170.03, 164.51, 160.88, 160.83, 156.47, 156.41, 152.63, 148.47, 147.11, 147.07, 143.07, 142.33, 138.49, 138.44, 135.28, 129.82, 129.66, 129.62, 128.89, 126.93, 126.81, 126.57, 126.49, 126.29, 119.27, 119.17, 45.57. 45.42, 35.33, 34.97, 33.63, 14.72, 14.17; Ms: [*m/z*, 535].

##### 4-(2-(2-((2-(2-(1-(2-Aminophenyl)ethylidene)hydrazineyl)-2-oxoethyl)thio)-4-oxoquinazolin-3(4H)-yl)ethyl)benzenesulfonamide (**18**)

Mp 264–265°, 85% yield; ^1^H NMR (700 MHz, DMSO-*d_6_*): δ 11.00 (s, 0.80H), 10.85 (s, 0.20H), 8.10 (d, 1H, *J* = 7.91 Hz), 7.82 (t, 3H, *J* = 13.86 Hz), 7.52 (t, 2.60H, *J* = 7.70 and 7.21 Hz), 7.47 (t, 1.4H, *J* = 15. 05 Hz), 7.43 (d, 1H, *J* = 8.05 Hz), 7.36 (s, 2.60H), 7.15 (s, 1.40H), 7.05 (tt, 1H, *J* = 7.63 and 7.52 Hz), 6.78 (d, 0.22H, *J* = 8.05 Hz), 6.70 (d, 0.78H, 8.09 Hz), 6.60 (t, 0.22H, *J* = 7.45 Hz), 7.53 (t, 0.78H, 7.50 Hz), 4.60 (s, 0.50H), 4.31 (t, 3.5H, *J* = 9.17 and 8.49 Hz), 3.12 (t, 2H, *J* = 8.49 and 8.40 Hz), 2.34 (ss, 3H); ^13^C NMR (175 MHz, DMSO-*d_6_*): δ 169.13, 164.41, 160.83, 156.41, 156.36, 155.05, 152.78, 148.41, 147.56, 147.12, 147.10, 143.11, 143.08, 142.33, 135.33, 135.25, 134.62, 132.66, 130.07, 129.74, 129.66, 129.56, 126.98, 126.61, 126.53, 126.51, 126.27, 119.28, 119.15, 117.71, 116.55, 115.72, 114.89, 45.58, 45.47, 35.57, 35.07, 33.65, 16.01, 15.03; Ms: [*m/z*, 550].

##### 4-(2-(2-((2-(2-(1-(4-Aminophenyl)ethylidene)hydrazineyl)-2-oxoethyl)thio)-4-oxoquinazolin-3(4H)-yl)ethyl)benzenesulfonamide (**19**)

Mp 308–309°, 84% yield; ^1^H NMR (700 MHz, DMSO-*d_6_*): δ 10.68 (s, (s, 0.63H), 10.57 (s, 0.37H), 8.09 (t, 1H, *J* = 9.66 and 8.54 Hz), 7.80 (ddd, 3H, *J* = 11.44 and 8.22 Hz), 7.56 (dd, 1.50H, *J* = 8.19 and 8.05 Hz), 7.50 9dd, 3H, *J* = 8.05 and 7.98 Hz), 7.45 (ddd, 1.50H, *J* = 5.18 and 8.12 Hz), 7.35 (s, 2H), 6.55 (dd, 2H, *J* = 8.19 and 8.40 Hz), 5.48 (d, 2H, *J* = 11.41 Hz), 4.66 (s, 1.22H), 4.31 (dd, 2H, *J* = 8.96 and 7.21 Hz), 4.25 (s, 0.78H), 3.12 (t, 2H, *J* = 14.84 Hz), 2.21 (s, 3H); ^13^C NMR (175 MHz, DMSO-*d_6_*): δ 169.44, 163.76, 160.90, 160.84, 156.52. 156.48, 153.98, 150.76, 150.57, 149.34, 147.13, 147.10, 143.09, 143.06, 142.36, 135.29, 135.25, 129.67, 129.64, 128.09, 127.78, 126.97, 126.94, 126.50, 126.31, 126.29, 125.60, 125.36, 119.27, 119.18, 113.68, 113.56, 45.56, 45.41, 35.37, 35.09, 33.64, 14.27, 13.77; Ms: [*m/z*, 550].

##### 4-(2-(2-((2-(2-(1-(4-Bromophenyl)ethylidene)hydrazineyl)-2-oxoethyl)thio)-4-oxoquinazolin-3(4H)-yl)ethyl)benzenesulfonamide (**20**)

Mp 302–303°, 86% yield; IR (KBr, cm^−1^) *ν*: 3295, 3258, 3138 (NH), 1753, 1680 (C = O), 1338, 1156 (O = S = O); ^1^H NMR (700 MHz, DMSO-*d_6_*): δ 11.02 (s, 0.65H), 10.87 (s, 0.35H), 8.09 (dd, 1H, *J* = 8.33 and 7.19 Hz), 7.81–7.41 (m, 5H), 8.09 (t, 2H, *J* = 8.33 Hz), 7.54–7.38 (m, 4H), 7.35 (s, 2H), 4.68 (s, 1.3H), 4.31 (t, 2.7H, *J* = 11.41 and 7.14 Hz), 3.12 (t, 2H, *J* = 15 Hz), 2.33 (s, 1H), 2.31 (s, 2H); ^13^C NMR (175 MHz, DMSO-*d_6_*): δ 170.04, 164.60, 160.86, 160.82, 156.40, 156.36, 151.33, 147.40, 147.10, 147.06, 143.08, 142.33, 137.68, 137.64, 135.30, 131.82, 131.76, 129.67, 129.63, 128.83, 128.59, 126.98, 126.92, 126.61, 126.51, 126.4, 126.34, 126.28, 123.30, 123.10, 119.27, 119.17, 45.58, 45.44, 35.31, 34.99, 33.63, 14.50, 14.01; Ms: [*m/z*, 613 and M + 2, 615].

##### 4-(2-(2-((2-(2-(1-(2-Chlorophenyl)ethylidene)hydrazineyl)-2-oxoethyl)thio)-4-oxoquinazolin-3(4H)-yl)ethyl)benzenesulfonamide (**21**)

Mp 273–275°, 84% yield; ^1^H NMR (700 MHz, DMSO-*d_6_*): δ 11.01 (s, 0.65H), 10.88 (s, 0.35H), 7.39 (dd, 1H, *J* = 7.22 and 10.92 Hz), 7.18 (m, 2H), 7.55–7.7.38 (M, 7H), 7.36 (S, 2H), 7.32–7.29 (M, 1H), 4.56 (s, 1.2H), 4.30 (s, 2.8H), 3.11 (t, 2H, *J* = 6.43 Hz), 2.32 (s, 1H), 2.31 (s, 2H); ^13^C NMR (175 MHz, DMSO-*d_6_*): δ 170.14, 164.80, 160.85, 156.39, 156.36, 156.11, 153.28, 149.20, 147.10, 147.05, 143.08, 142.34, 139.45, 139.26, 135.28, 129.91, 129.65, 129.63, 127.89, 126.92, 126.63, 126.50, 126.29, 119.28, 119.15, 45.58, 45.53, 33.62, 18.96, 18.51.

##### 4-(2-(2-((2-(2-(1-(3-Chlorophenyl)ethylidene)hydrazineyl)-2-oxoethyl)thio)-4-oxoquinazolin-3(4H)-yl)ethyl)benzenesulfonamide (**22**)

Mp 297–298°, 85% yield; ^1^H NMR (700 MHz, DMSO-*d_6_*): δ 11.07 (s, 0.67H), 10.92 (s, 0.33H), 8.08 (dd, 1H, *J* = 8.12 and 7.83 Hz), 7.92 (ss, 1H), 7.82–7.74 (m, 4H), 7.51 (d, 1H, *J* = 8.05 Hz), 7.48 (t, 2.50H, *J* = 14.64 Hz), 7.46 (d, 1.5H, *J* = 7.98 Hz), 7.43 (d, 1H, *J* = 10.99 Hz), 7.36 (s, 2H), 4.71 (s, 1.40H), 4.32 (t, 2.6H, *J* = 7.07 Hz), 3.12 (t, 2H, *J* = 15.66 Hz), 2.35 (s, 1H), 2.32 (s. 2H); ^13^C NMR (175 MHz, DMSO-*d_6_*): δ 170.13, 160.87, 160.82, 156.38, 147.07, 147.02, 143.08, 142.33, 140.63, 135.31, 135.25, 133.87, 133.7, 130.80, 130.74, 129.67, 129.62, 129.39, 126.98, 126.94, 126.51, 126.49, 126.34, 126.29, 126.14, 125.53, 125.32, 119.27, 119.19, 45.58, 45.43, 35.2, 35.25, 33.64, 14.61, 14.11; Ms: [*m/z*, 569, M + 2, 571].

##### 4-(2-(2-((2-(2-(1-(4-Chlorophenyl)ethylidene)hydrazineyl)-2-oxoethyl)thio)-4-oxoquinazolin-3(4H)-yl)ethyl)benzenesulfonamide (**23**)

Mp 278–280°, 88% yield; IR (KBr, cm^−1^) *ν*: 3385, 3290, 3153 (NH), 1752, 1680 (C = O), 1339, 1157 (O = S = O); ^1^H NMR (700 MHz, DMSO-*d_6_*): δ 11.01 (s, 0.65H), 10.87 (s, 0.35H), 8.08 (t, 1H, *J* = 7.39 and 6Hz), 7.88 (d, 1H, *J* = 7.95 Hz), 7.82–7.76 (m, 4H), 7.57 (d, 1H, *J* = 8.12 Hz), 7.54–7.44 (m, 4H), 7.39 (d, 1H, *J* = 8.19 Hz), 7.36 (S, 2H), 4.69 (s, 1.2H), 4.29 (t, 2.8H, *J* = 8.51 Hz), 3.10 (t, 2H, *J* = 15. 29 Hz), 2.34 (s, 1H), 2.31 (s, 2H); ^13^C NMR (175 MHz, DMSO-*d_6_*): δ 170.04, 166.63, 160.88, 160.83, 156.37, 156.10, 147.12, 147.06, 143.07, 142.36, 142.33, 137.32, 135.31, 135.23, 134.34, 129.65, 128.90, 128.84, 128.56, 128.32, 126.86, 126.56, 126.50, 119.20, 45.46, 34.46, 33.60, 14.55, 14.06; Ms: [*m/z*, 569 and M + 2, 571].

##### 4-(2-(2-((2-(2-(1-(4-Fluorophenyl)ethylidene)hydrazineyl)-2-oxoethyl)thio)-4-oxoquinazolin-3(4H)-yl)ethyl)benzenesulfonamide (**24**)

Mp 299–300°, 88% yield; IR (KBr, cm^−1^) *ν*: 3272, 3144 (NH), 1755, 1680 (C = O), 1339, 1157 (O = S = O); ^1^H NMR (700 MHz, DMSO-*d_6_*): δ 10.97 (s, 0.65H), 10.83 (s, 0.35H), 8.09 (dd, 1H, *J* = 7.78 and 7.71 Hz), 7.91 (t, 1.3H, *J* = 12.88 Hz), 7.84 (0.7H, *J* = 13.02 Hz), 7.82–7.76 (m, 3H), 7.54–7.44 (m, 3H), 7.40 (d, 1H, *J* = 8.12 Hz), 7.36 (s, 2H), 7.25 (t, 2H, *J* = 8.57 Hz), 4.69 (s, 1.3H), 4.33–4.30 (m, 2.7H), 3.12 (t, 2H, *J* = 12.06 Hz), 2.34 (s, 1H), 2.32 (s, 2H); ^13^C NMR (175 MHz, DMSO-*d_6_*): δ 169.98, 164.49, 163.90, 162.50, 160.87, 160.83, 156.38, 151.67, 147.54, 147.11, 147.07, 143.08, 142.33, 135.30, 135.02, 135.00, 129.67, 129.63, 129.07, 129.02, 128.80, 128.75, 126.92, 126.48, 126.33, 119.27, 119.17, 115.82, 115.75, 115.70, 115.63, 45.57, 45.43, 35.31, 35.00, 33.63, 14.72, 14.20; Ms: [*m/z*, 553].

##### 4-(2-(4-Oxo-2-((2-oxo-2-(2-(1-(p-tolyl)ethylidene)hydrazineyl)ethyl)thio)quinazolin-3(4H)-yl)ethyl)benzenesulfonamide (**25**)

Mp 288–290°, 86% yield; ^1^H NMR (700 MHz, DMSO-*d_6_*): δ 10.91 (s, 0.65H), 10.77 (s, 0.35H), 8.08 (t, 1H, *J* = 12.67 and 7.91), 7.81 (d, 1.3H, *J* = 7.56 Hz), 7.77 (t, 3H, *J* = 8.47 Hz), 7.69 (d, 0.7H, *J* = 7.77 Hz), 7.54 (d, 0. 33H, *J* = 8.19 Hz), 7.51 (d, 0. 67H, *J* = 7.70 Hz), 7.48 (dd, 2H, *J* = 7.70 Hz), 7.46 (d, 0.65H, *J* = 7.35 Hz), 7.41 (d, 0.35H, *J* = 8.12 Hz), 7.36 (s, 2H), 7.22 (d, 2H, *J* = 7.77 Hz), 4.68 (s, 1.3H), 4.31 (dd, 2.7H, *J* = 16.66 and 12.60 Hz), 3.11 (t, 2H, *J* = 7.88 and 7.56 Hz), 2.33 (s, 3H), 2.31 (s, 1H), 2.29 (s, 2H); ^13^C NMR (175 MHz, DMSO-*d_6_*): δ 169.93, 164.36, 160.88, 160.83, 156.43, 156.41, 152.69, 148.52, 147.11, 147.08, 143.09, 143.07, 142.33, 139.45, 139.27, 135.72, 135.64, 135.29, 129.67, 129.62, 129.47, 129.38, 126.97, 126.93, 126.76, 126.50, 126.48, 126.30, 119.27, 119.18, 45.57, 45.42, 35.33, 34.97, 33.63, 21.30, 14.62, 14.10.

##### 4-(2-(4-Oxo-2-((2-oxo-2-(2-(1-(pyridin-2-yl)ethylidene)hydrazineyl)ethyl)thio)quinazolin-3(4H)-yl)ethyl)benzenesulfonamide (**26**)

Mp 300–301°, 83% yield; IR (KBr, cm^−1^) *ν*: 3375, 3259, 3143 (NH), 1751, 1680 (C = O), 1340, 1157 (O = S = O); ^1^H NMR (700 MHz, DMSO-*d_6_*): δ 11.12 (s, 0.66H), 10.97 (s, 0.34H), 8.62 (dd, 1H, *J* = 4.41 and 4.13 Hz), 8.15 (d, 0.66H, *J* = 7.98 Hz), 8.07 (t, 1H, *J* = 12.32 and 7.91 Hz), 8.02 (d, 0.34H, *J* = 8.05 Hz), 7.84–7.75 (m, 4H), 7.53–7.39 (m, 5H), 7.36 (s, 2H), 4.73 (s, 1.3H), 4.32 (t, 2.7H, *J* = 8.26 and 8.04 Hz), 3.12 (t, 2H, *J* = 6.39 and 8.20Hz), 2.44 (s, 1H), 2.41 (s, 2H); ^13^C NMR (175 MHz, DMSO-*d_6_*): δ 170.14, 164.83, 160.86, 160.83, 156.37, 156.33, 155.43, 155.39, 152.57, 149.20, 149.10, 147.09, 147.05, 143.09, 143.08, 142.32, 137.09, 135.29, 129.67, 129.63, 126.9, 126.93, 126.61, 126.52, 126.4, 126.31, 126.27, 124.59, 124.4, 120.77, 120.46, 119.26, 119.1, 45.59, 45.44, 35.31, 34.86, 33.63, 12.90, 12.48; Ms: [*m/z*, 536 and M + 1, 537].

##### 4-(2-(4-Oxo-2-((2-oxo-2-(2-(1-(pyridin-3-yl)ethylidene)hydrazineyl)ethyl)thio)quinazolin-3(4H)-yl)ethyl)benzenesulfonamide (**27**)

Mp 297–280°, 88% yield; ^1^H NMR (700 MHz, DMSO-*d_6_*): δ 11.10 (s, 0.66H), 10.95 (s, 0.34H), 9.08 (s, (s, 0.66H), 8.96 (s, 0.34H), 8.60 (d, 1H, *J* = 3.99 Hz), 8.22 (d, 0.66H, *J* = 7.91 Hz), 8.15 (d, 0.34H, *J* = 7.91 Hz), 9.09 (d, 0.34H, *J* = 7.98 Hz), 8.07 (0.66H, *J* = 7.91 Hz), 7.81 (d, 1H, *J* = 7.35 Hz), 7.91 (d, 1.35H, *J* = 7.84 Hz), 7.75 (d, 0.65H, *J* = 7.70 Hz), 7.54 (d, 0.35H, *J* = 8.19 Hz), 7.51 (d, 0.65H, *J* = 7.70 Hz), 7.49 (d, 1.3H, H= 7.63 Hz), 7.45 (dd, 2H, *J* = 9.73 and 13.63 Hz), 7.39 (d, 0.7H, *J* = 8.12 Hz), 7.36 (s, 2H), 4.71 (s, 1.3H), 4.32 (d, 2.7H, *J* = 11.06 Hz), 3.12 (t, 2H, *J* = 15.75 Hz), 2.38 (s, 1H), 2.36 (s, 2H); ^13^C NMR (175 MHz, DMSO-*d_6_*): δ 170.16, 164.70, 160.87, 160.83, 156.39, 156.37, 150.50, 150.33, 147.98, 147.86, 147.10, 147.06, 146.42, 143.09, 143.07, 142.33, 135.32, 135.29, 134.11, 134.06, 133.91, 129.67, 129.63, 126.98, 126.93, 126.62, 126.51, 126.49, 126.29, 123.93, 123.89, 119.27, 119.17, 45.59, 45.45, 35.29, 34.97, 33.64, 33.62, 14.58, 14.02; Ms: [*m/z*, 536 and M + 1, 537].

### 4.2. CA Inhibition

The inhibition assay for the hCA I, II, IX, and XII isozymes was carried out with the SX.18MV-R stopped-flow instrument (Applied Photophysics, Oxford, UK) according to the method reported previously [58,61].

## Figures and Tables

**Table 1:**
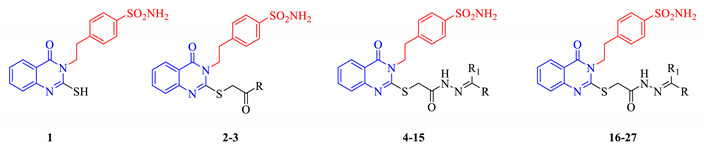
Inhibition data of human CA isoforms hCA I, II, IX, and XII for hydrazonoquinazolines **2**–**27** and (AAZ) standard drug.

Comps	R	R_1_	Ki (nM) ^a^				Selectivity Analysis			
			hCA I	hCA II	hCA IX	hCA XII	hCA I/IX	hCA I/XII	hCA II/IX	hCA II/XII
**1**			31.5	0.62		0.59		53.12		1.05
**2**	OCH_2_CH_3_		106.7	21.3	10.5	34.7	10.16	3.07	2.02	0.61
**3**	NHNH_2_		87.6	16.9	52.1	5.4	1.68	16.22	0.32	3.12
**4**	Ph	H	152.4	52.6	61.7	38.4	2.47	3.96	0.85	1.37
**5**	2-Cl-Ph	H	940.3	251.3	26.4	46.1	35.6	20.39	9.51	5.45
**6**	4-Cl-Ph	H	567.6	126.8	89.1	67.8	6.37	8.37	1.42	1.87
**7**	2,4-di-Cl-Ph	CH_3_	2354	432.8	22.0	89.4	107	26.33	19.67	4.84
**8**	3,4-di-Cl-Ph	H	1827	324.9	34.8	63.4	52.5	28.81	9.33	5.12
**9**	2,6-di-Cl-Ph	H	2256	164.8	49.2	55.9	45.85	40.35	3.35	2.95
**10**	2-F-Ph	H	274.1	35.8	19.7	22.9	13.91	11.96	1.81	1.56
**11**	4-F-Ph	H	132.0	49.5	15.4	15.8	8.57	8.35	3.21	3.13
**12**	2-CH_3_-Ph	H	337.8	69.4	29.6	63.5	11.41	5.31	2.34	1.09
**13**	2-NO_2_-Ph	H	991.7	569.4	58.5	21.4	16.95	46.34	9.73	26.60
**14**	4-NO_2_-Ph	H	635.4	220.7	26.9	30.5	23.62	20.83	8.20	7.23
**15**	4-*N*-(CH_3_)_2_-Ph	H	582.0	82.1	88.6	6.8	6.56	85.58	0.92	12.07
**16**	3-pyridyl	H	207.9	23.8	37.8	20.8	5.5	10.0	0.63	1.14
**17**	Ph	CH_3_	124.3	67.5	55.7	12.7	2.23	9.78	1.21	5.31
**18**	2-NH_2_-Ph	CH_3_	824.3	35.2	36.7	18.3	22.46	45.04	0.96	1.92
**19**	4-NH_2_-Ph	CH_3_	439.5	59.3	5.8	15.9	75.77	27.64	10.22	3.72
**20**	4-Br-Ph	CH_3_	1356	698.2	99.6	88.8	13.6	15.27	7.01	7.86
**21**	2-Cl-Ph	CH_3_	1145	324.2	45.3	58.0	25.27	19.74	7.15	5.59
**22**	3-Cl-Ph	CH_3_	867.2	339.4	75.3	46.9	11.51	18.49	4.50	7.23
**23**	4-Cl-Ph	CH_3_	563.5	157.0	65.0	61.2	8.67	9,20	2.41	2.56
**24**	4-F-Ph	CH_3_	186.9	39.5	28.5	25.4	6.55	7.35	1.38	1.55
**25**	4-CH_3_-Ph	CH_3_	627.3	126.0	43.9	57.5	14.28	10.90	2.87	2.19
**26**	2-pyridyl	CH_3_	52.8	22.8	68.2	28.5	0.77	1.85	0.33	0.80
**27**	3-pyridyl	CH_3_	238.4	10.8	33.7	34.7	7.07	6.87	0.32	0.31
**AAZ**			250.0	12.0	25.0	5.7	10	43.86	0.48	2.10

^a^ Mean from three different assays by a stopped-flow technique (errors were in the range of ±5–10% of the reported values).

## Data Availability

It can be found in supplementary materials.

## Supplementary Materials

The following supporting information can be downloaded at: https://www.mdpi.com/article/10.3390/molecules27227703/s1, ^1^H NMR and ^13^C NMR of compounds **2**–**27**.
