# Supplementary material for: Carbonic Anhydrase Inhibition Activities of Schiff’s Bases Based on Quinazoline-Linked Benzenesulfonamide
Source: Molecules. 2022 Nov 9;27(22):7703. doi: 10.3390/molecules27227703 (PMC9697198; doi:10.3390/molecules27227703)

Supporting Information

Carbonic Anhydrase Inhibition Activities of Schiff's Bases Based on Quinazoline-Linked Benzenesulfonamide

Adel S. El-Azab [1,*], Alaa A.-M. Abdel-Aziz [1], Hazem A. Ghabbour [2], Silvia Bua [3], Alessio Nocentini [3], Hamad M. Alkahtani [1], Nawaf A. Alsaif [1], Mohamed H. M. Al-Agamy [4] and Claudiu T. Supuran [3,*]

[1] Department of Pharmaceutical Chemistry, College of Pharmacy, King Saud University, P.O. Box 2457, Riyadh 11451, Saudi Arabia
[2] Department of Medicinal Chemistry, Faculty of Pharmacy, Mansoura University, Mansoura 35516, Egypt
[3] Department of Neurofarba, Sezione di Scienze Farmaceutiche Nutraceutiche, Università degli Studi di Firenze, Via U. Schiff 6, 50019 Sesto Fiorentino, Florence, Italy
[4] Department of Pharmaceutics and Microbiology, College of Pharmacy, King Saud University, P.O. Box 2457, Riyadh 11451, Saudi Arabia
* Correspondence: adelazab@ksu.edu.sa (A.S.E.-A.); claudiu.supuran@unifi.it (C.T.S.)

Content: ^{1}H NMR & ^{13}C NMR of compounds 2-27 (page S2-S29).

Compound 2 ^{1}H NMR & ^{13}C NMR

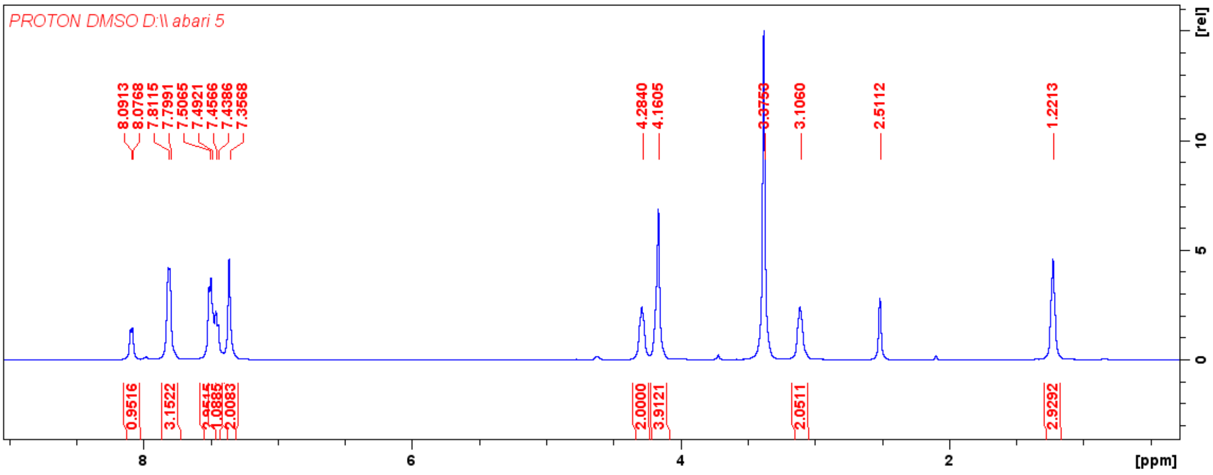

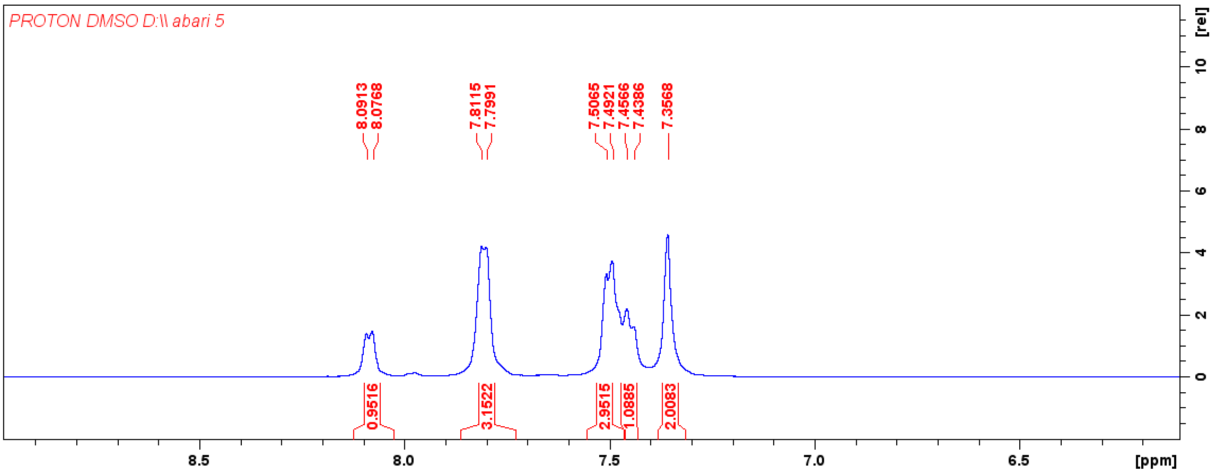

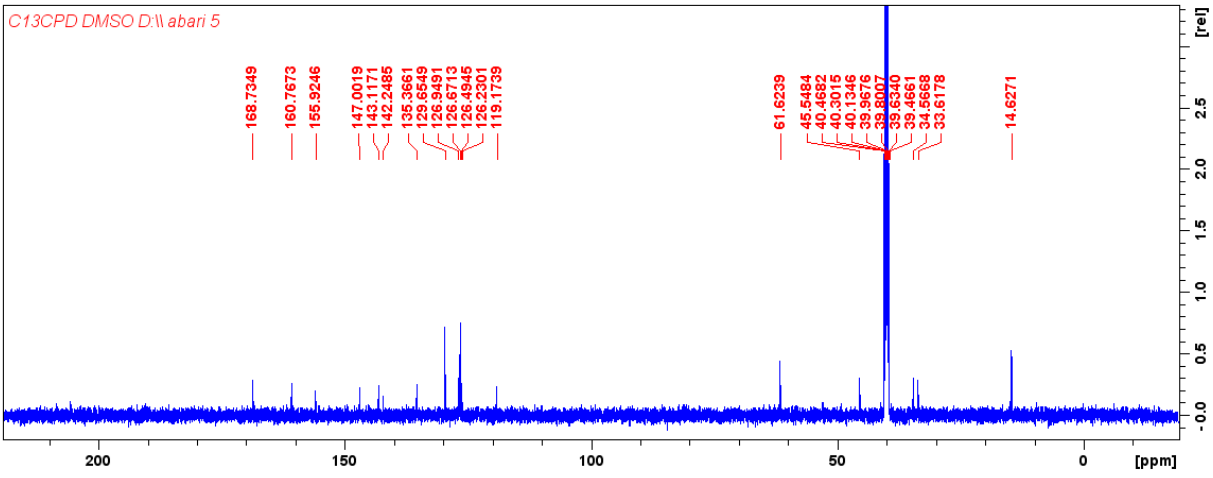

Compound 3 ^{1}H NMR

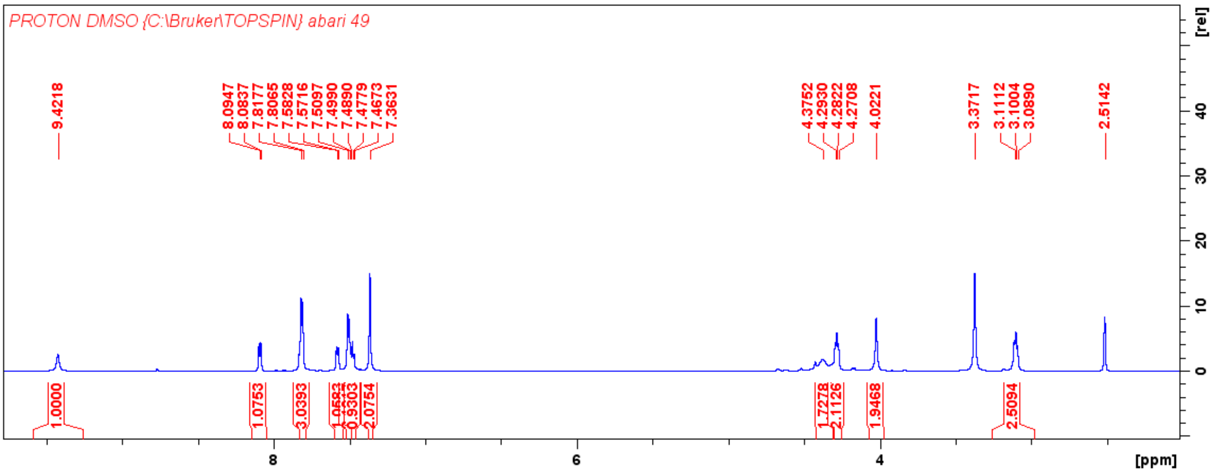

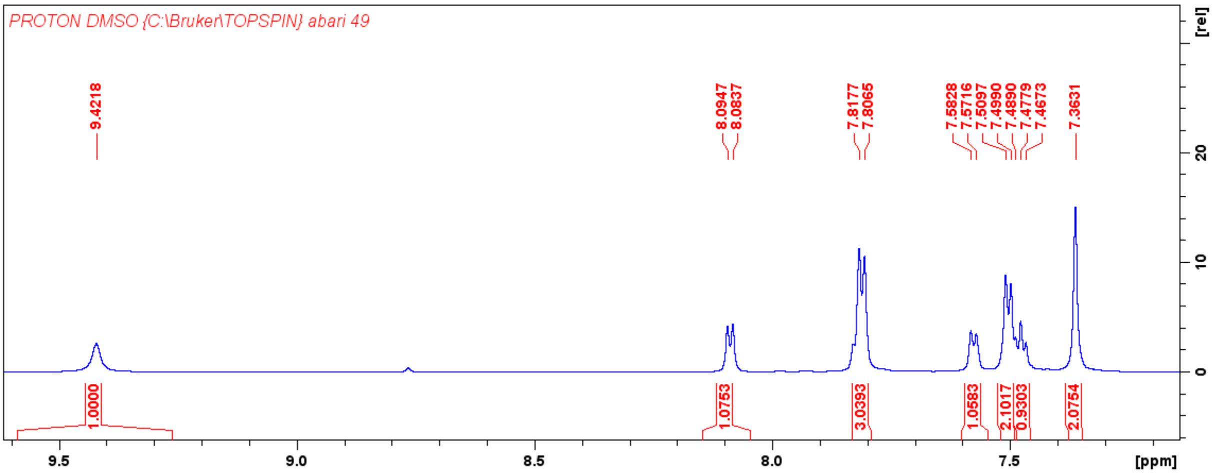

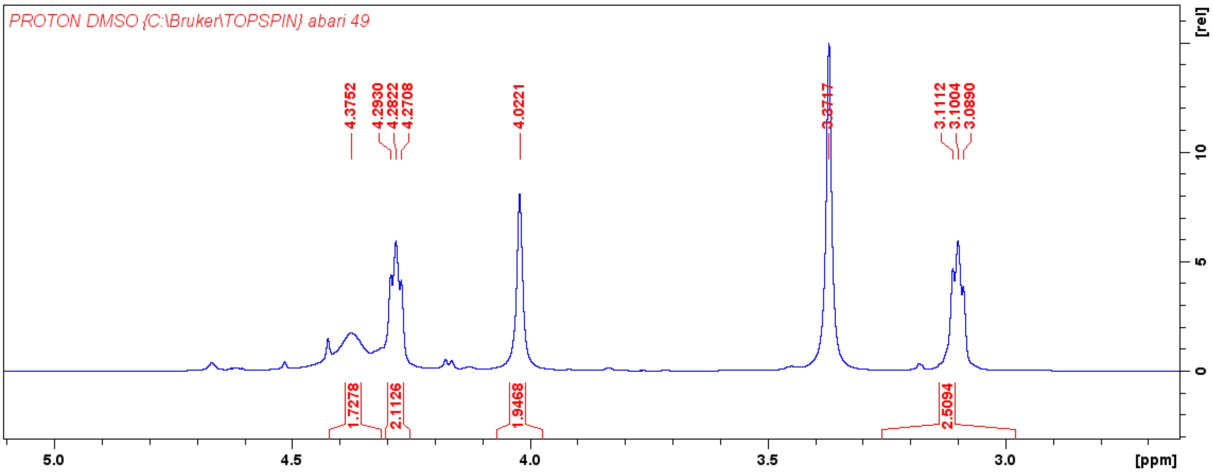

Compound 3 ^{13}C NMR

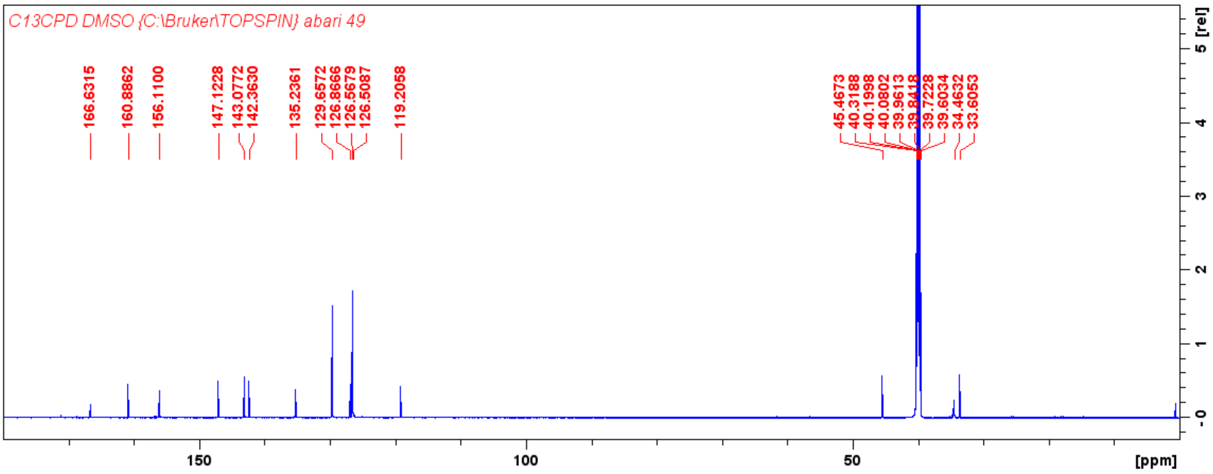

Compound 4 ^{1}H NMR

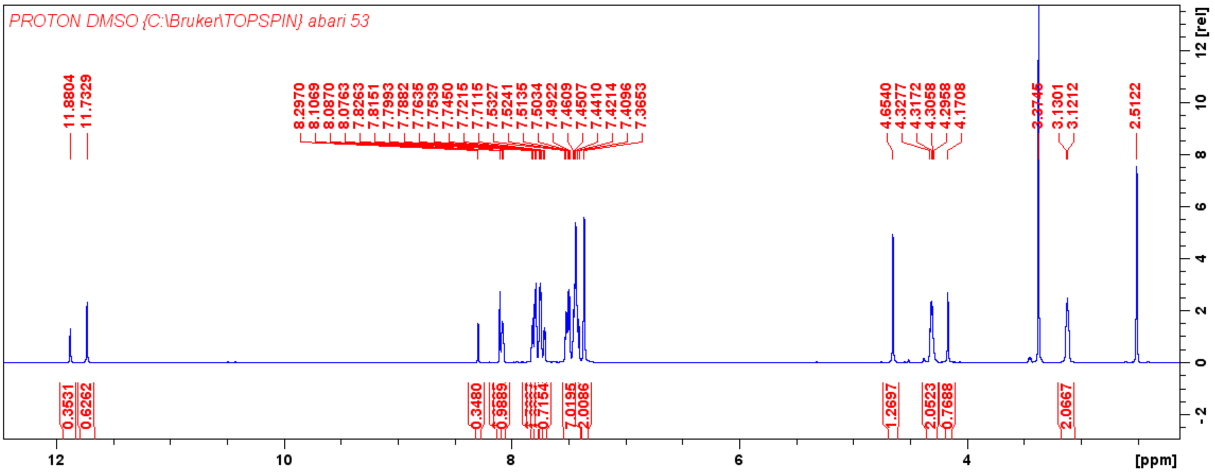

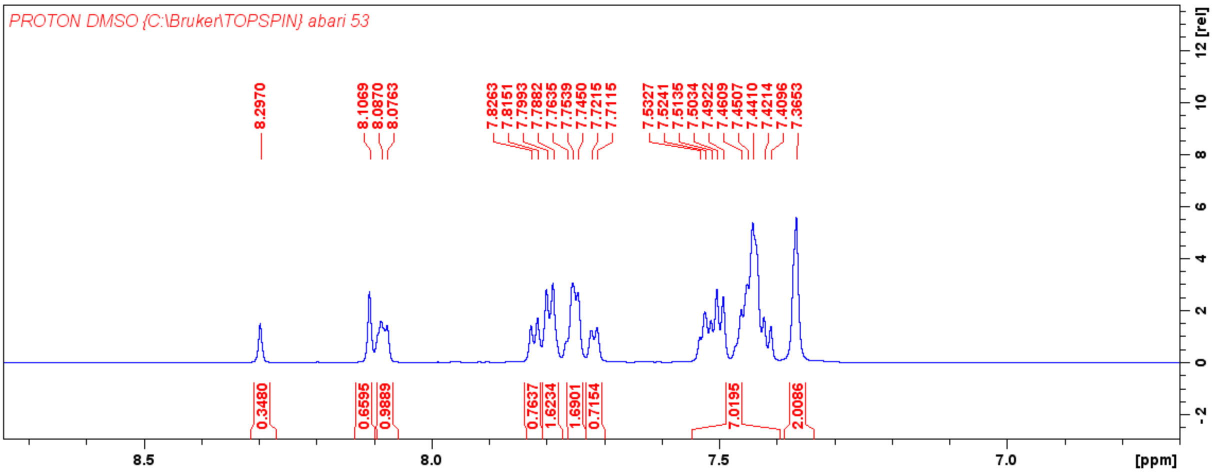

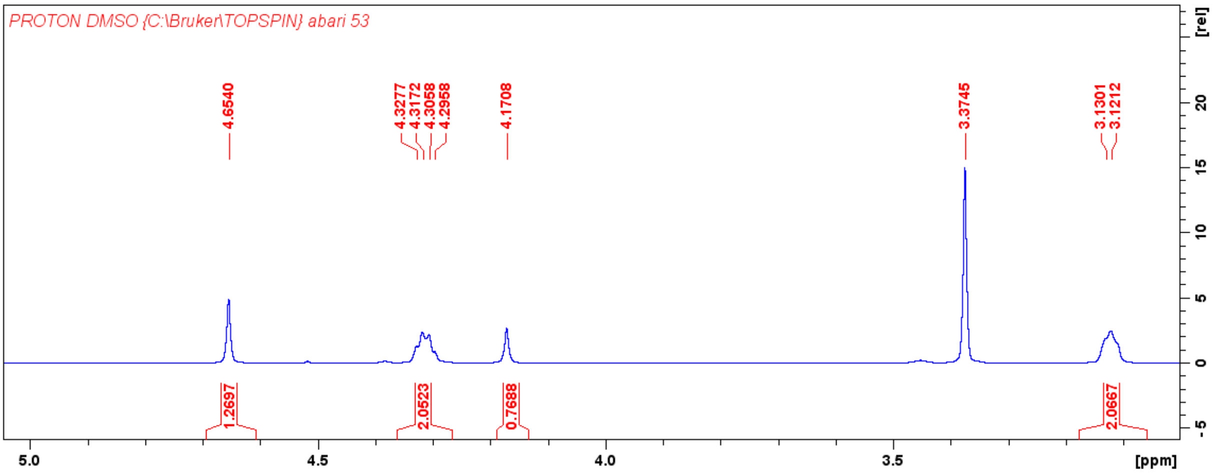

Compound 4 ^{13}C NMR

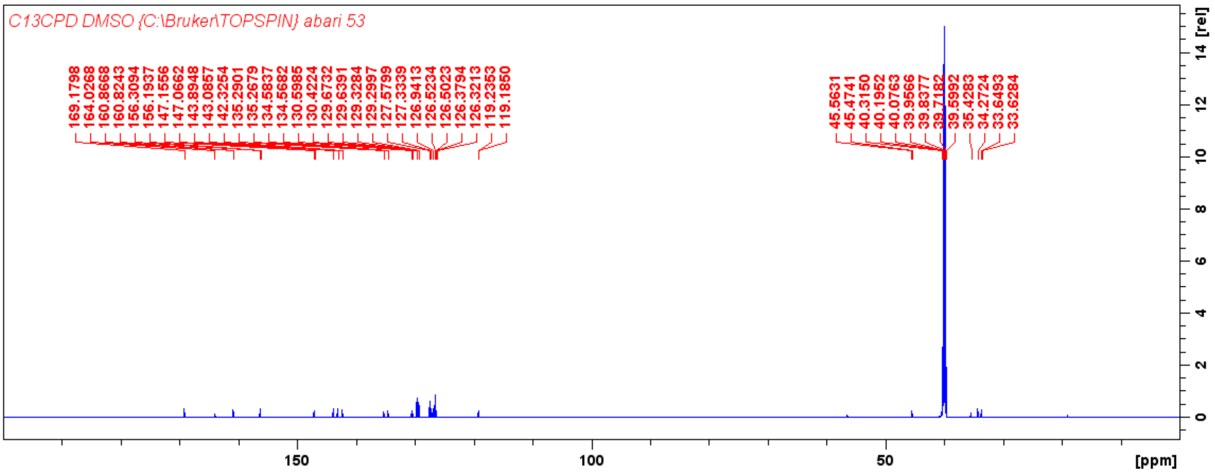

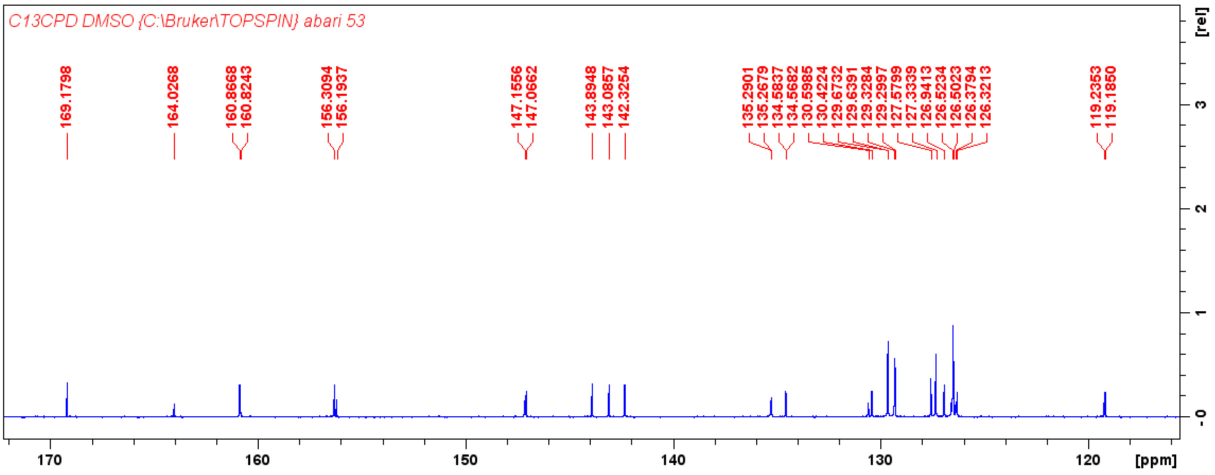

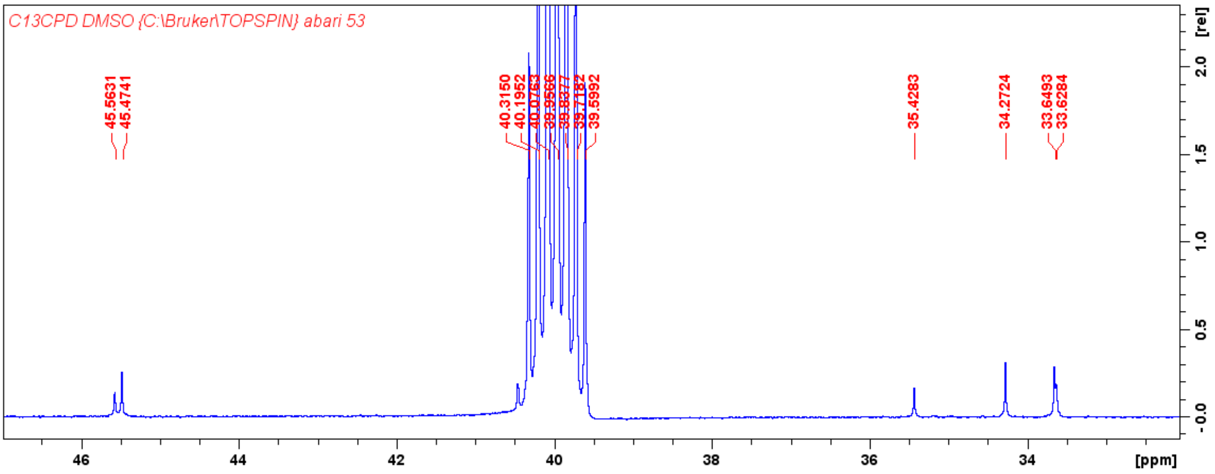

Compound 5 ¹H NMR

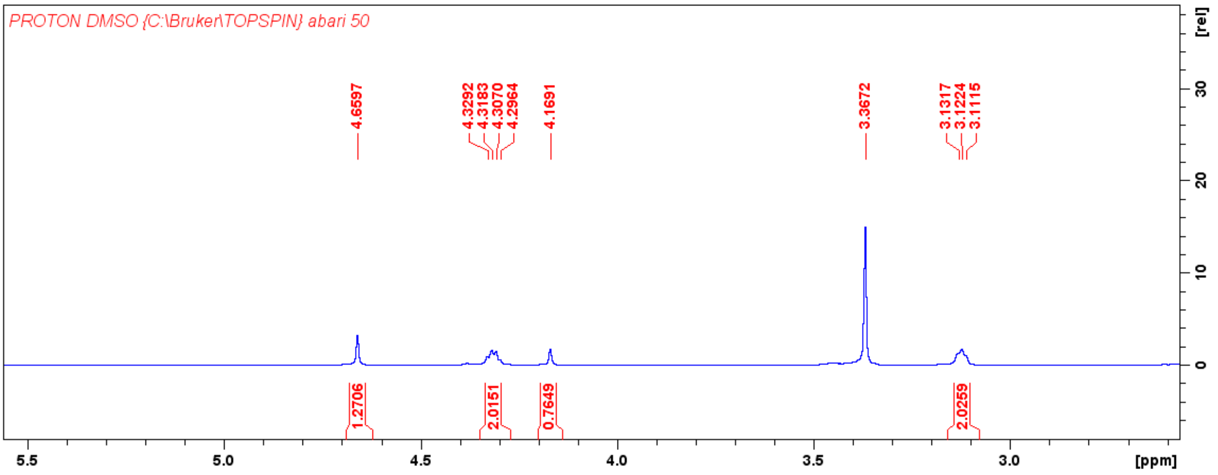

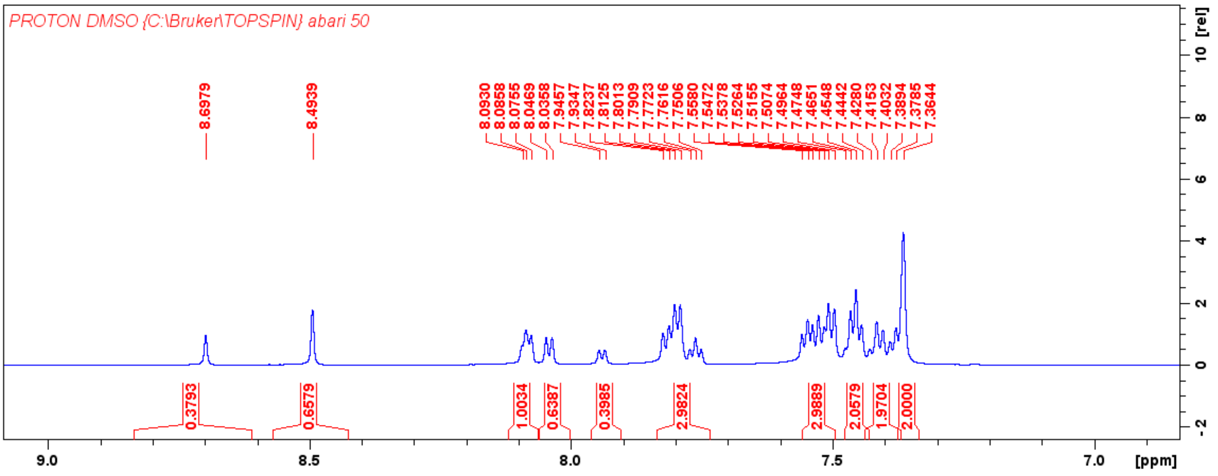

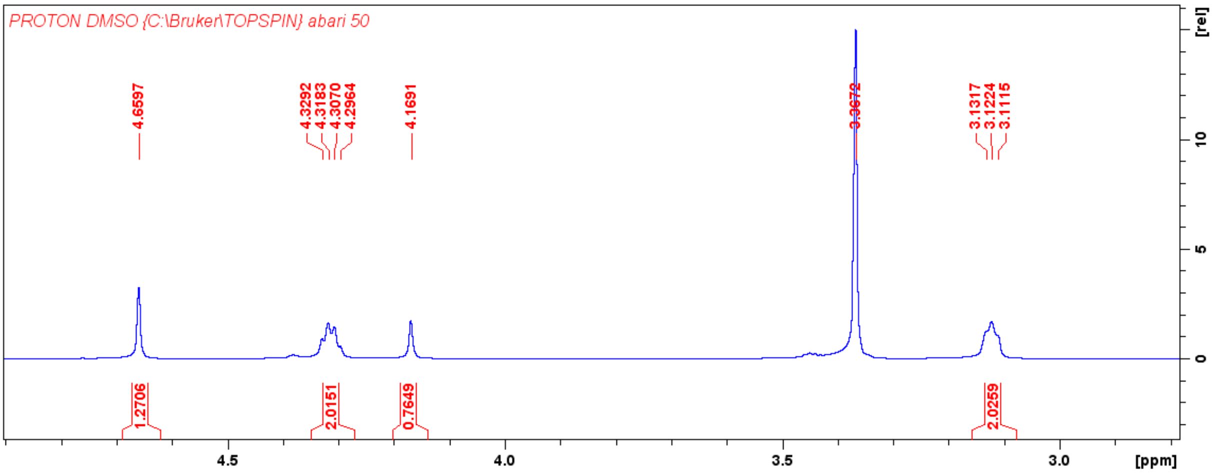

Compound 5 ^{13}C NMR

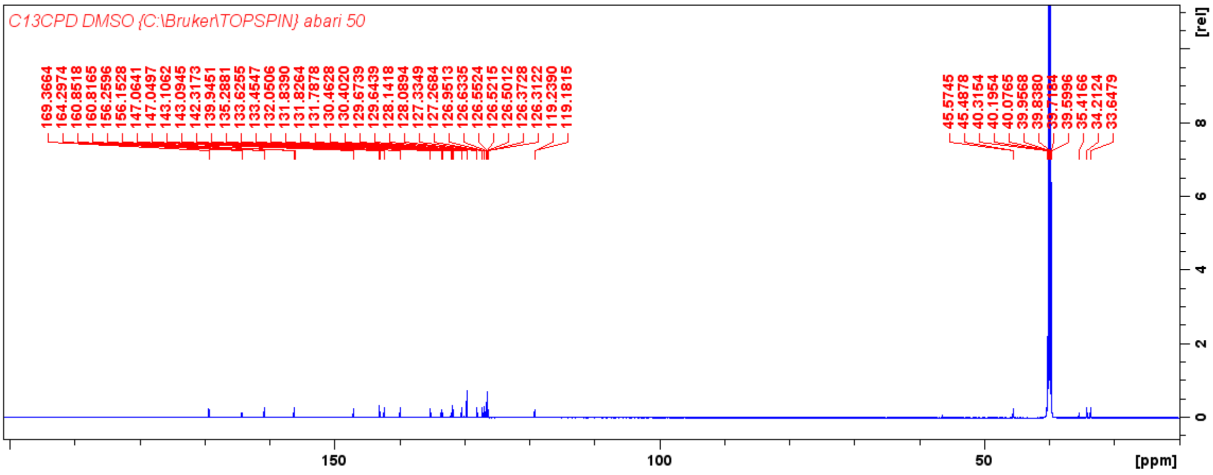

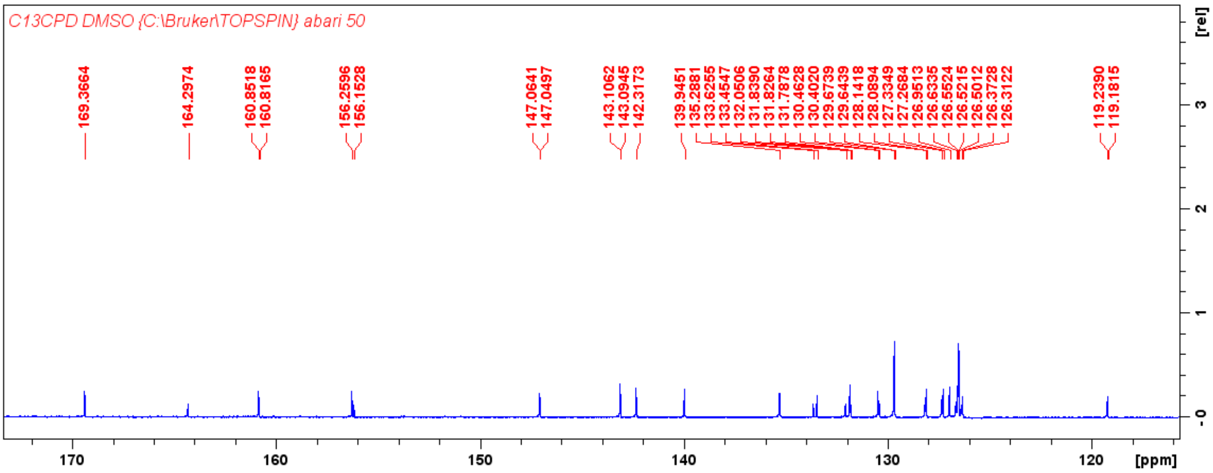

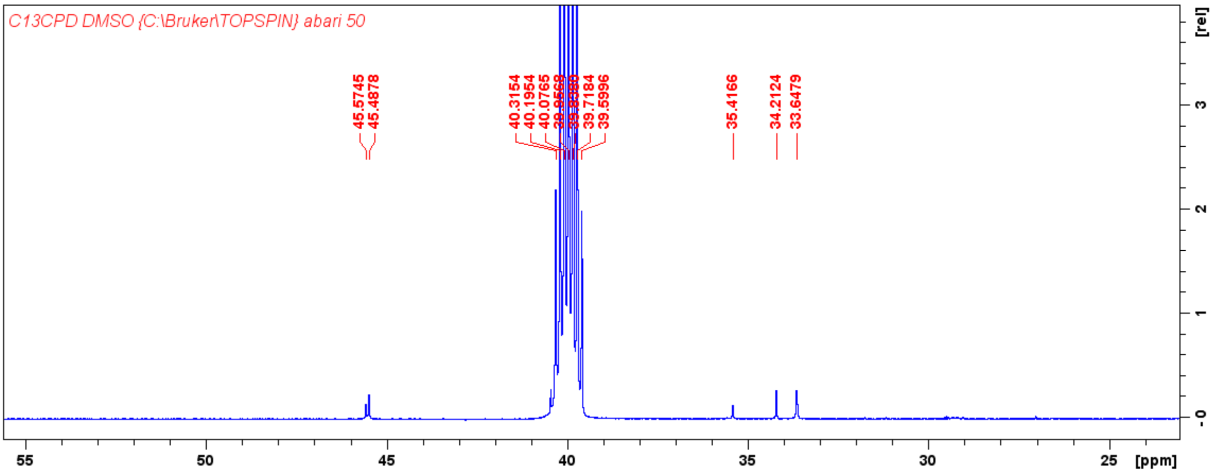

Compound 7 ^{1}H NMR

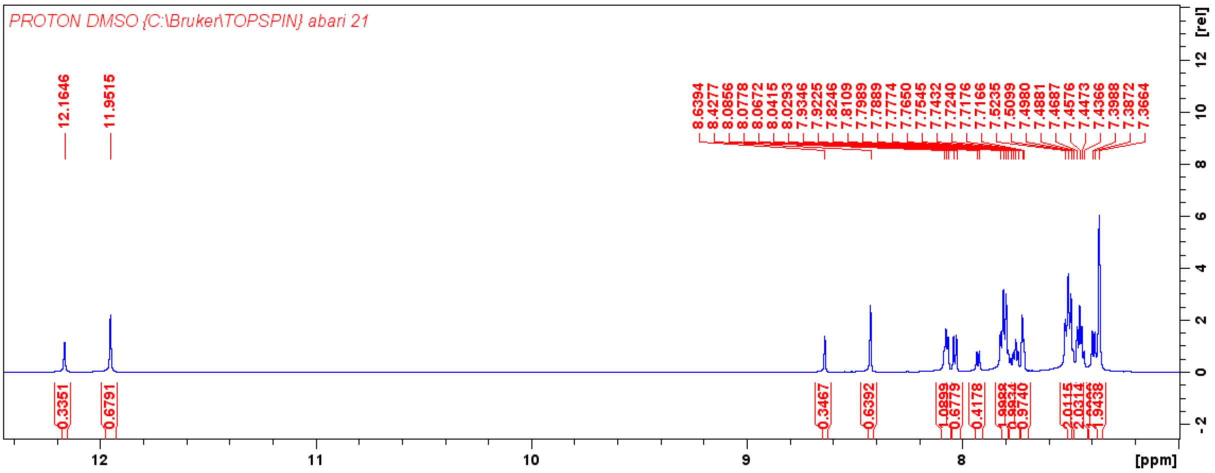

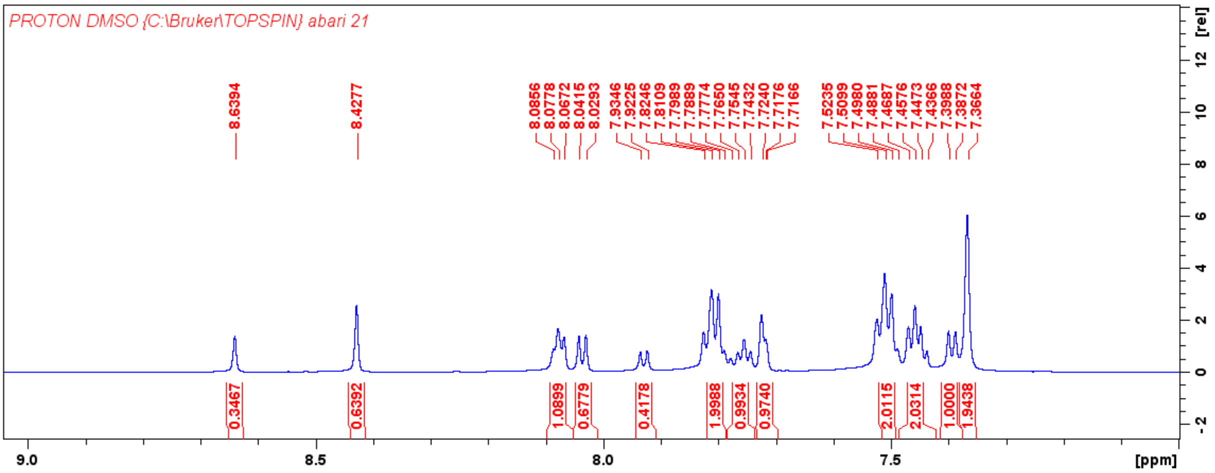

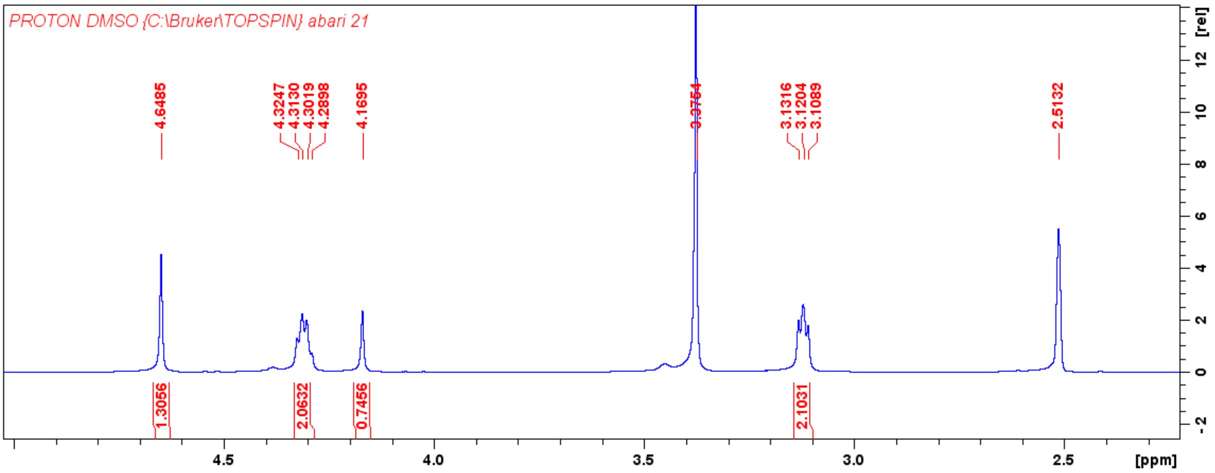

Compound 7 ^{13}C NMR

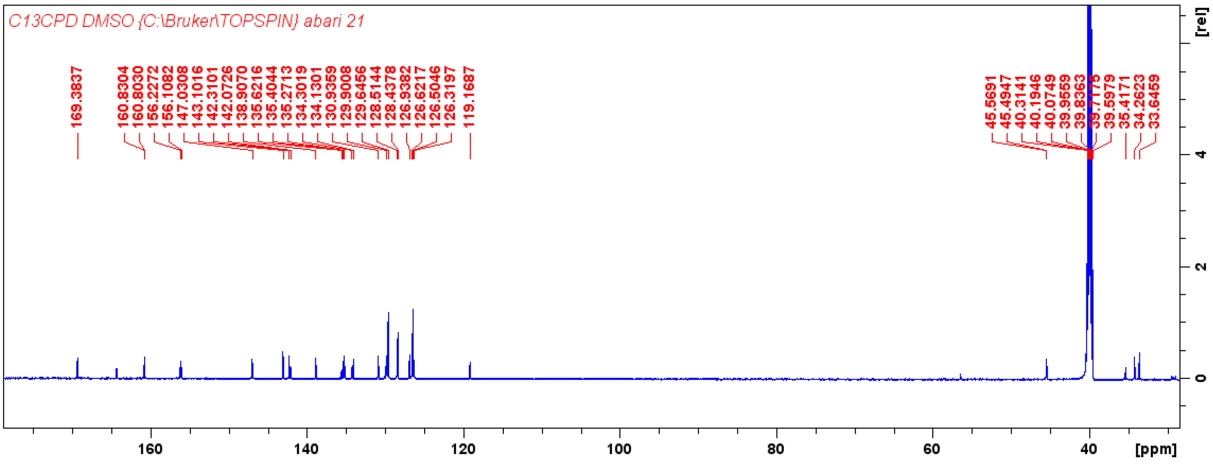

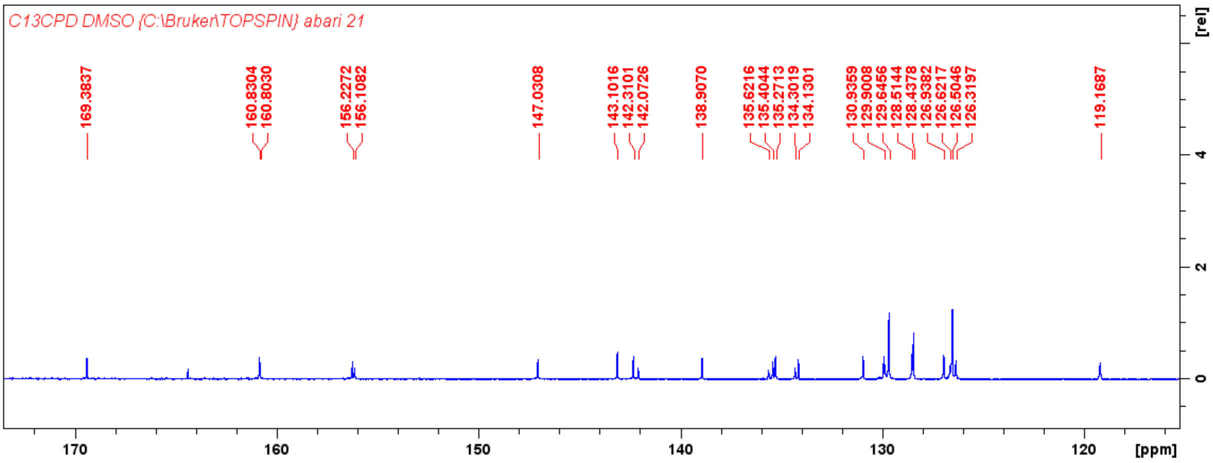

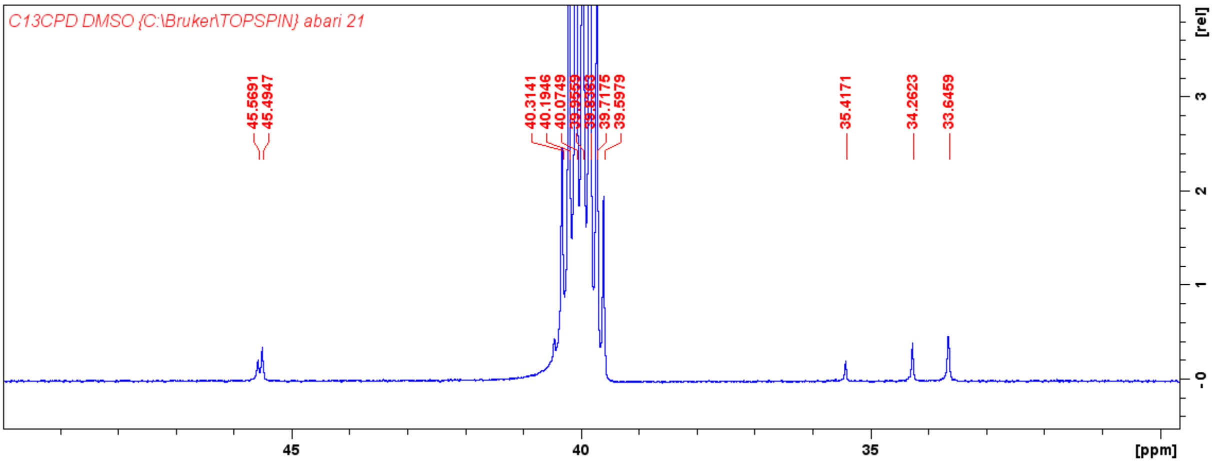

Compound 12 NMR

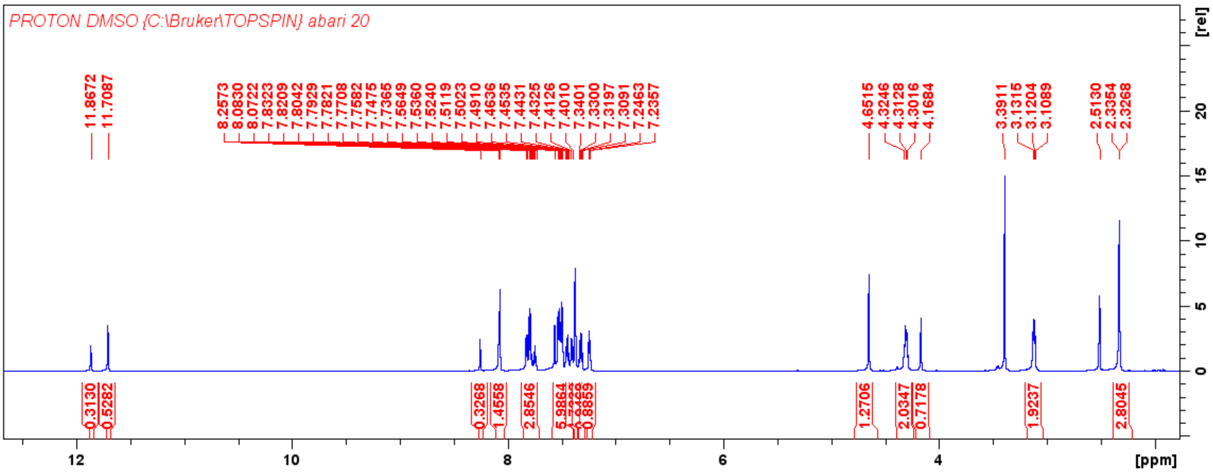

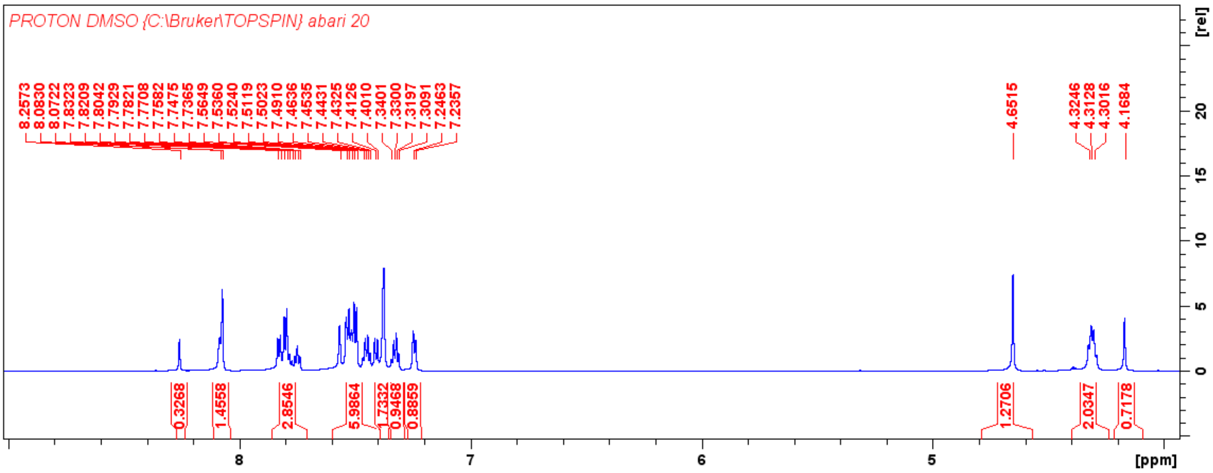

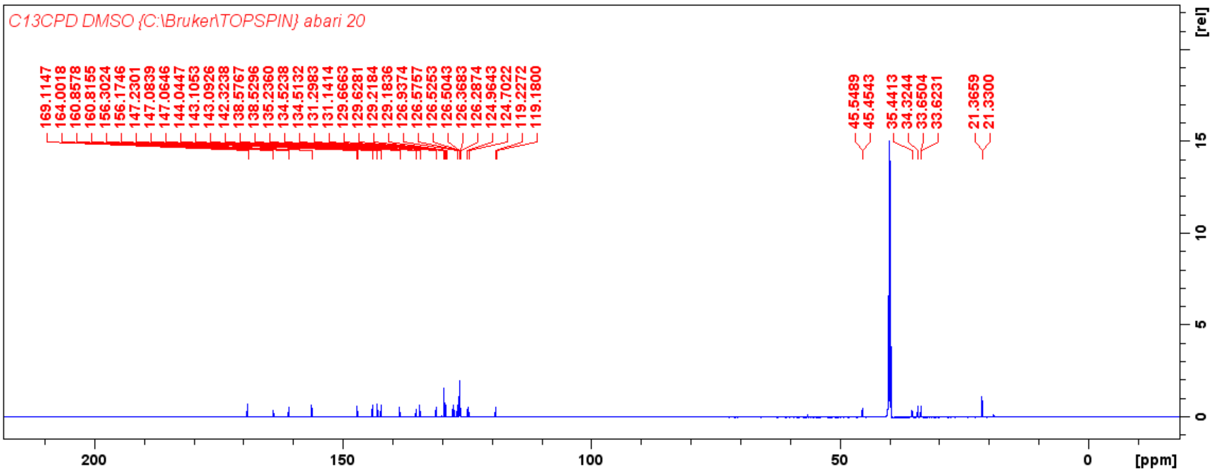

Compound 13 ^{1}H NMR

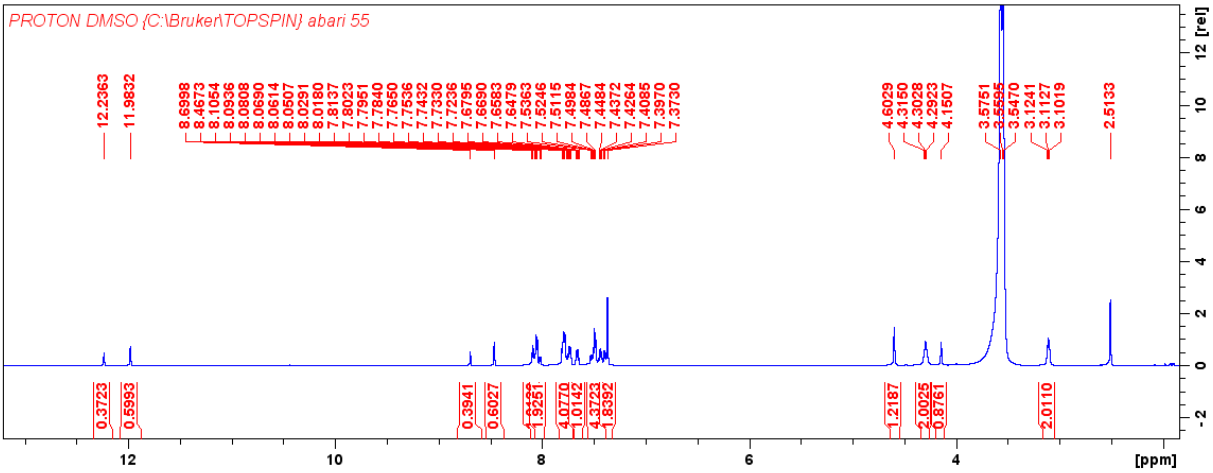

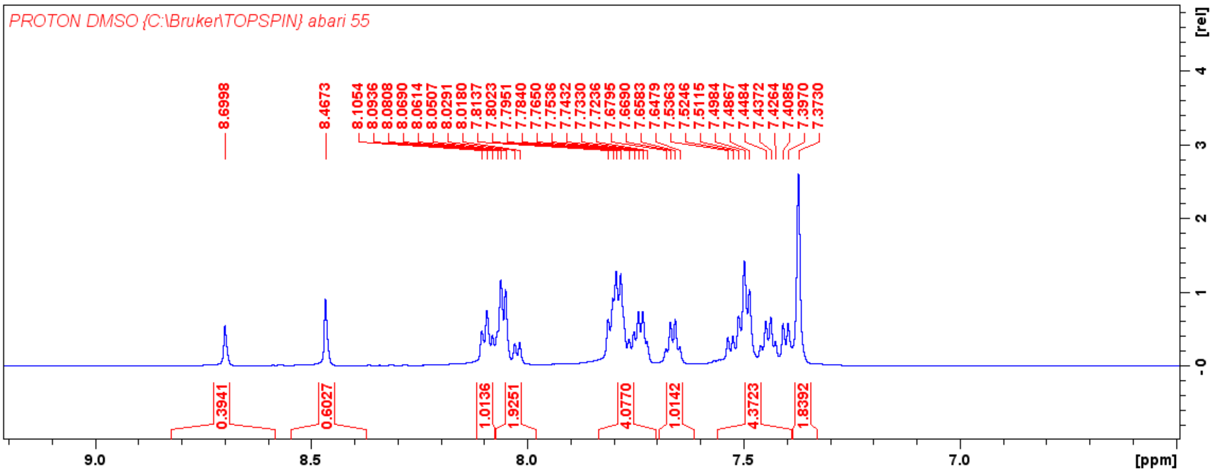

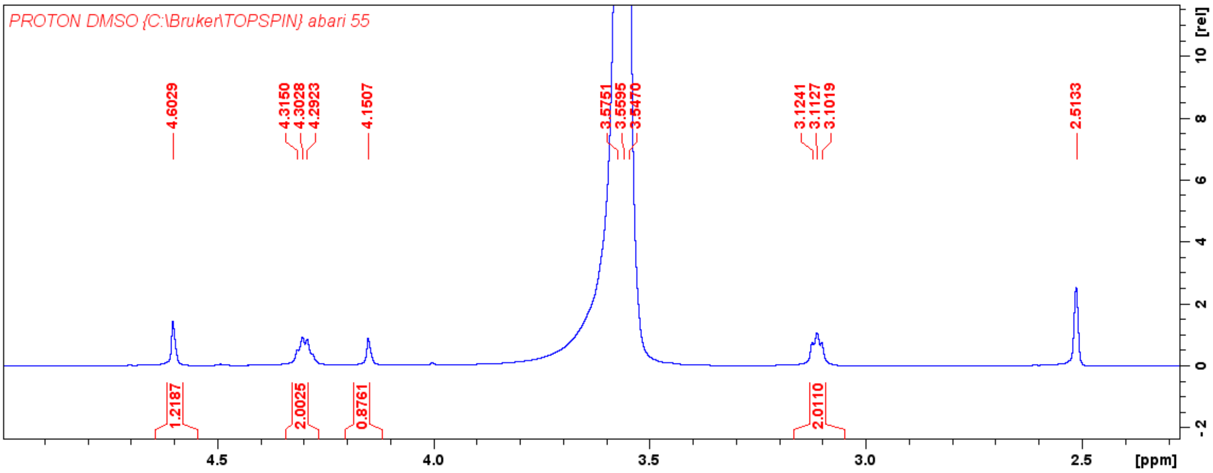

Compound 13 ^{13}C NMR

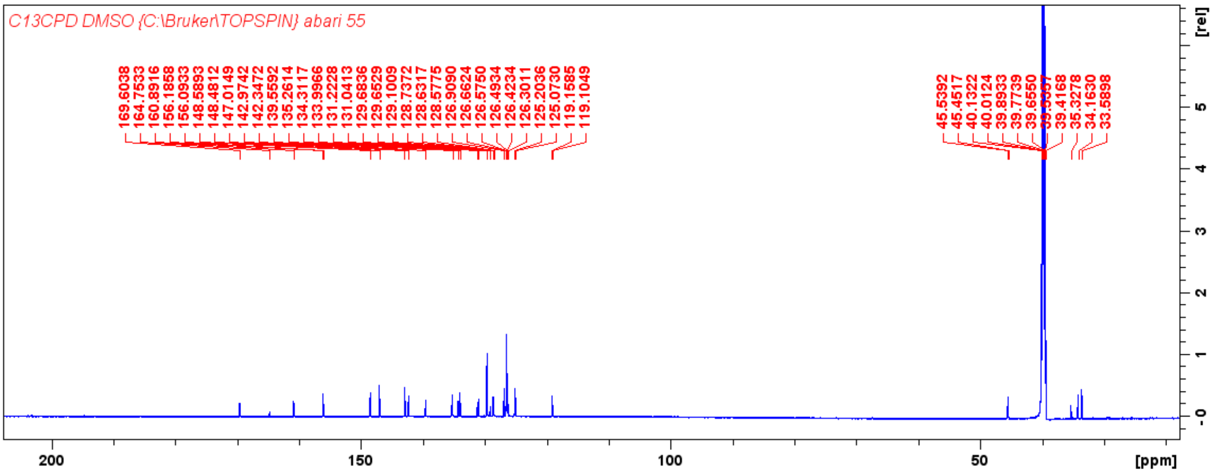

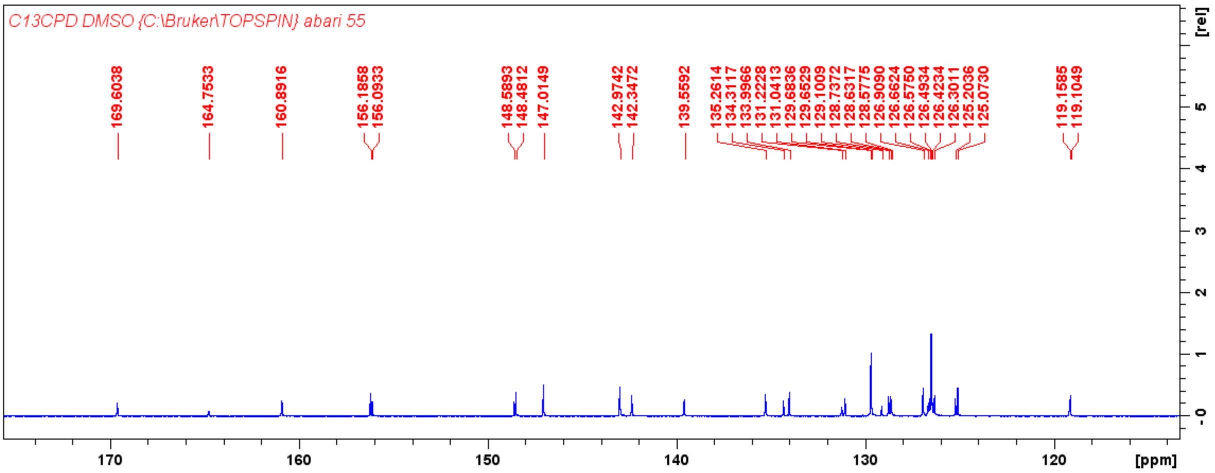

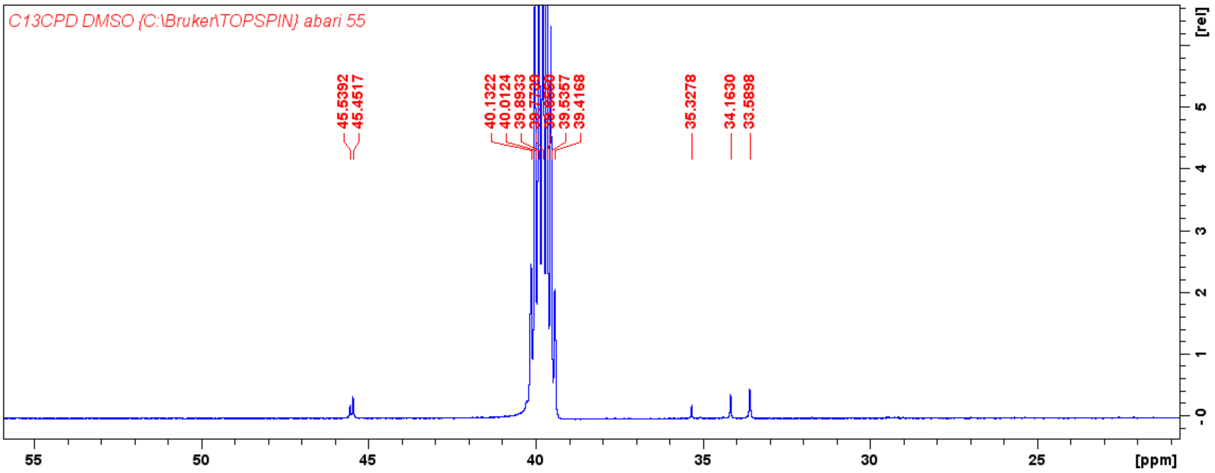

Compound 14 ^{1}H NMR

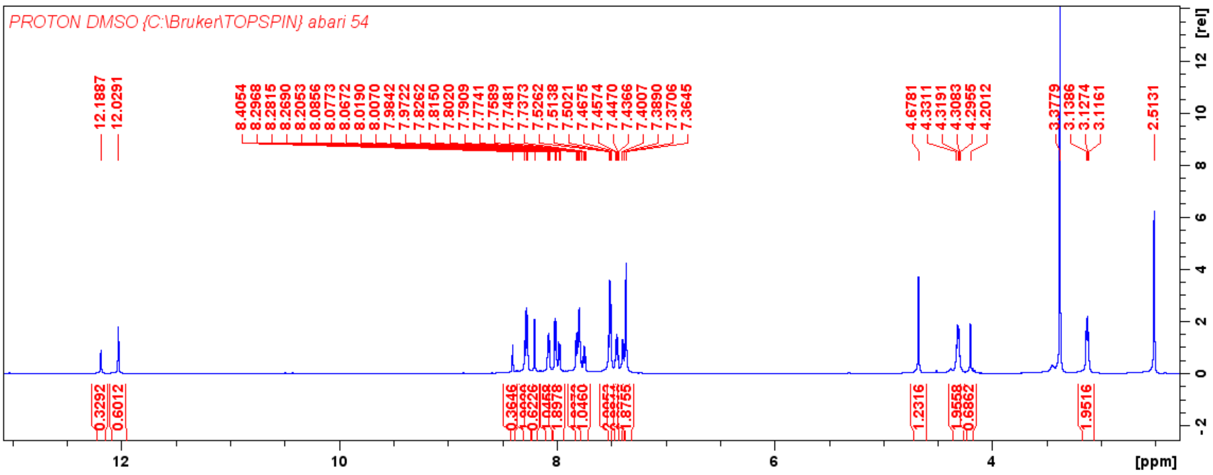

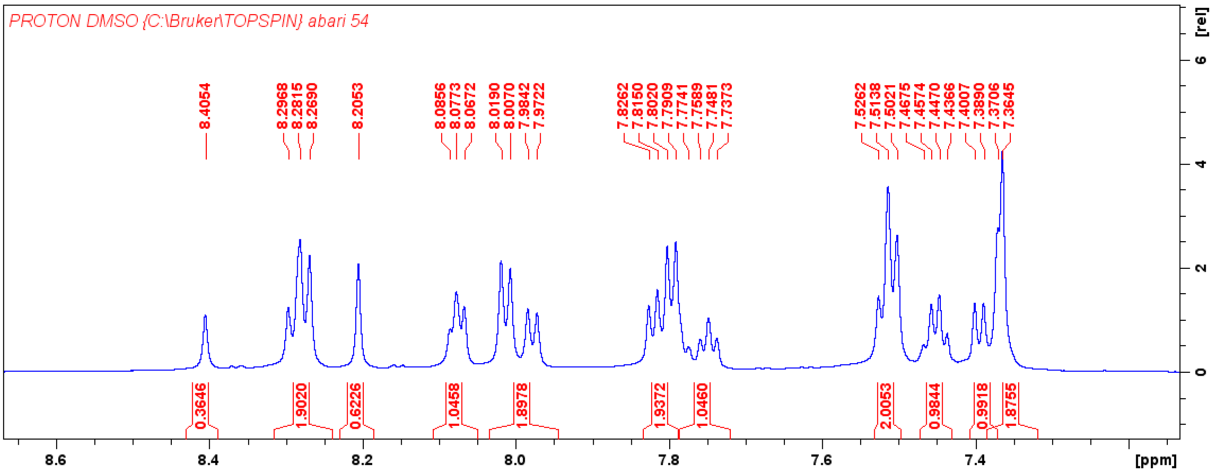

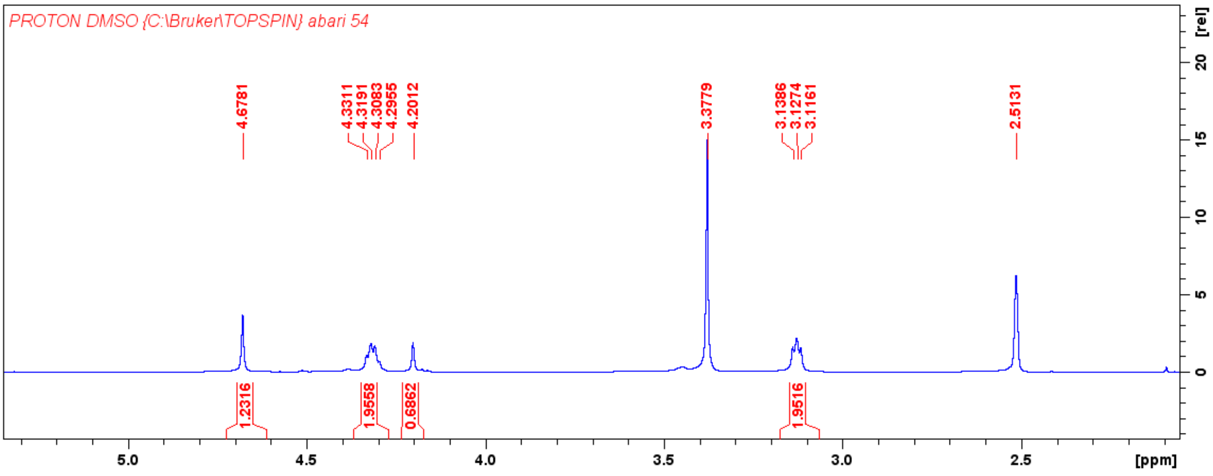

Compound 14 ^{13}C NMR

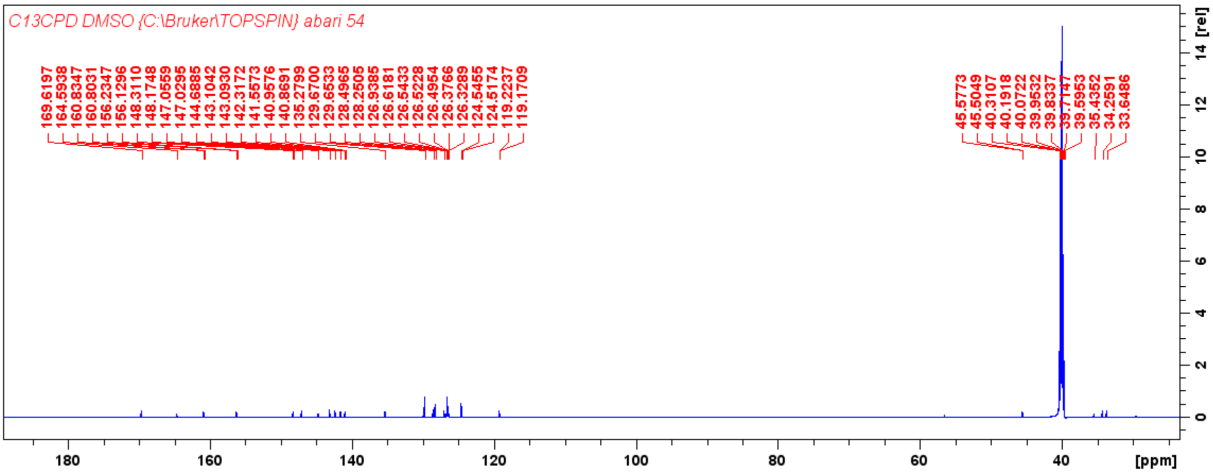

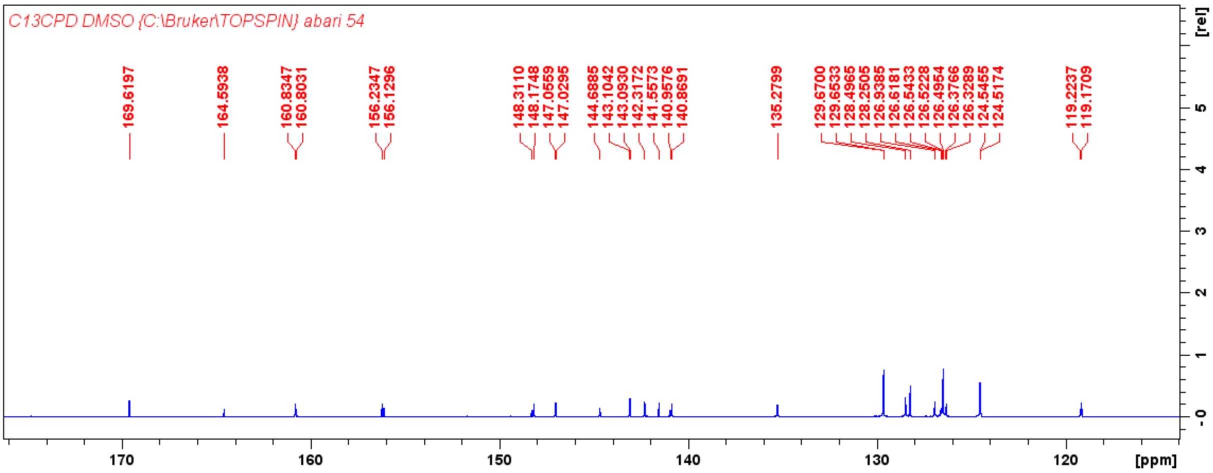

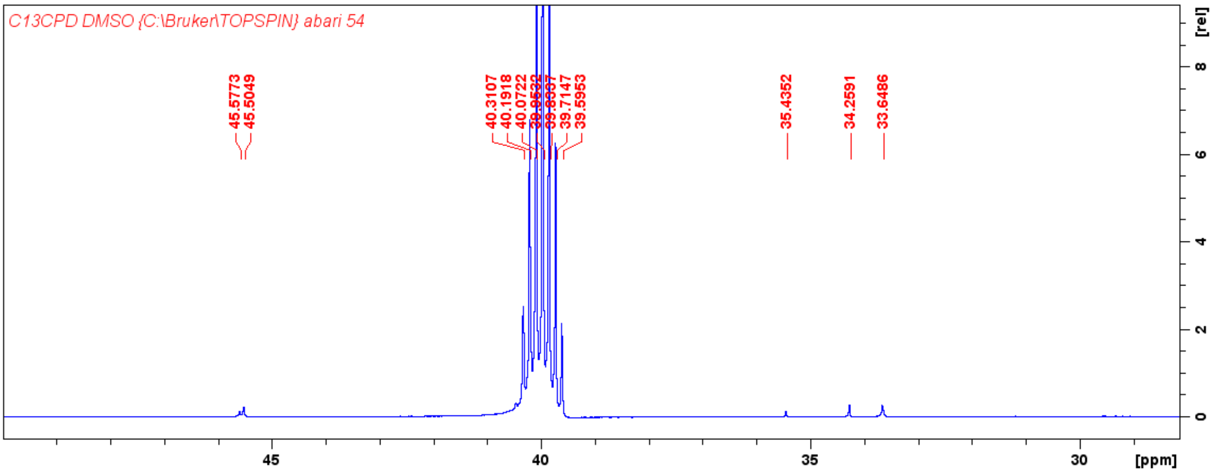

Compound 15 ^{1}H NMR

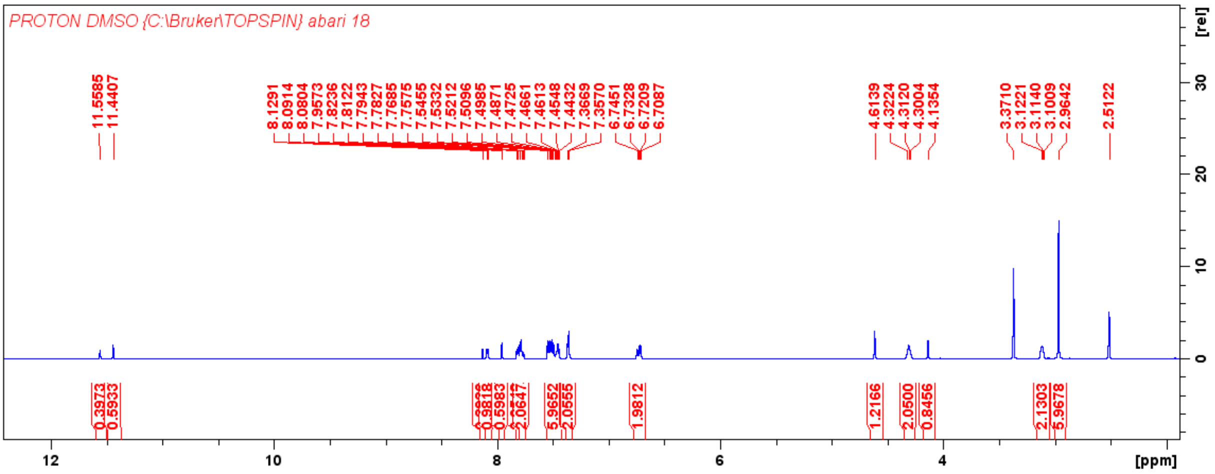

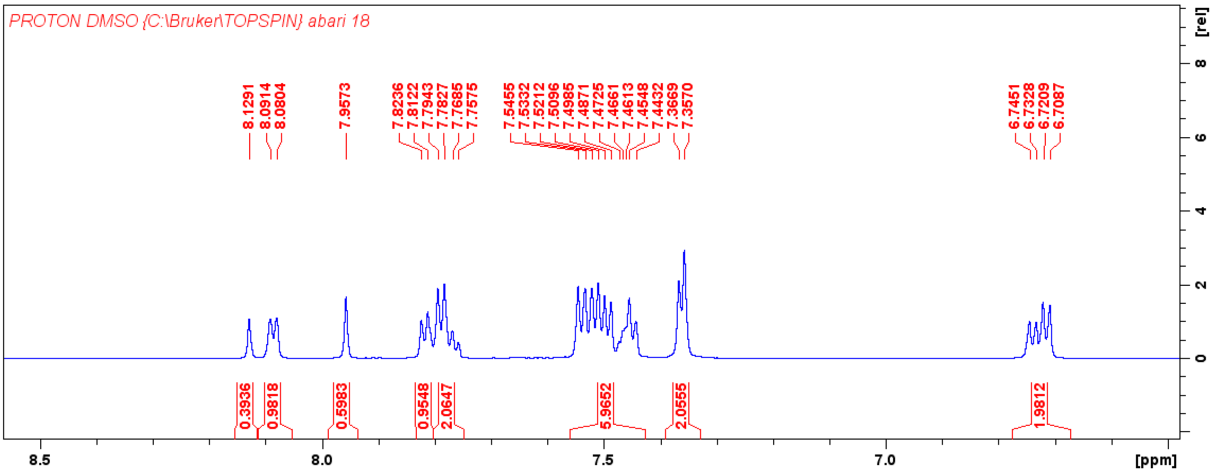

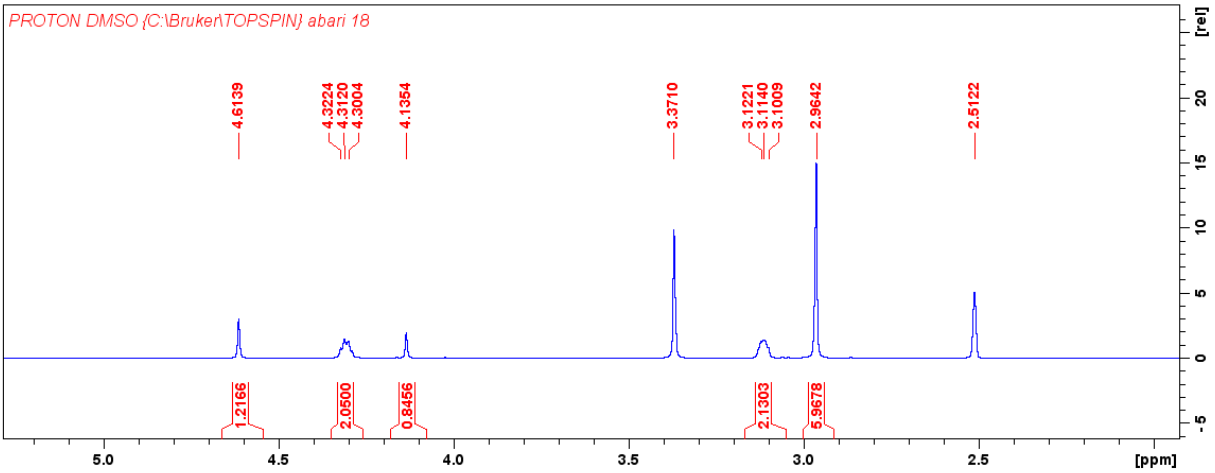

Compound 15 ^{13}C NMR

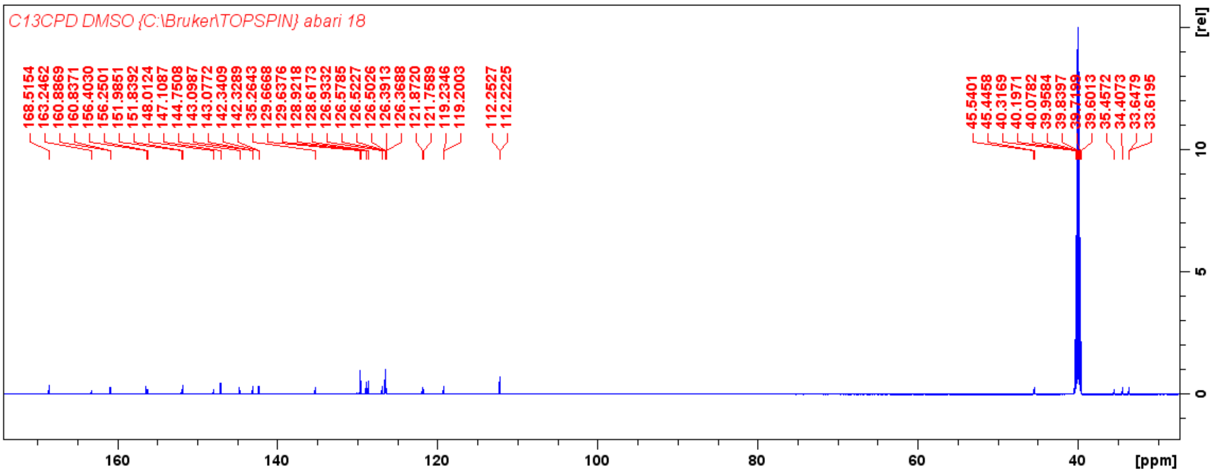

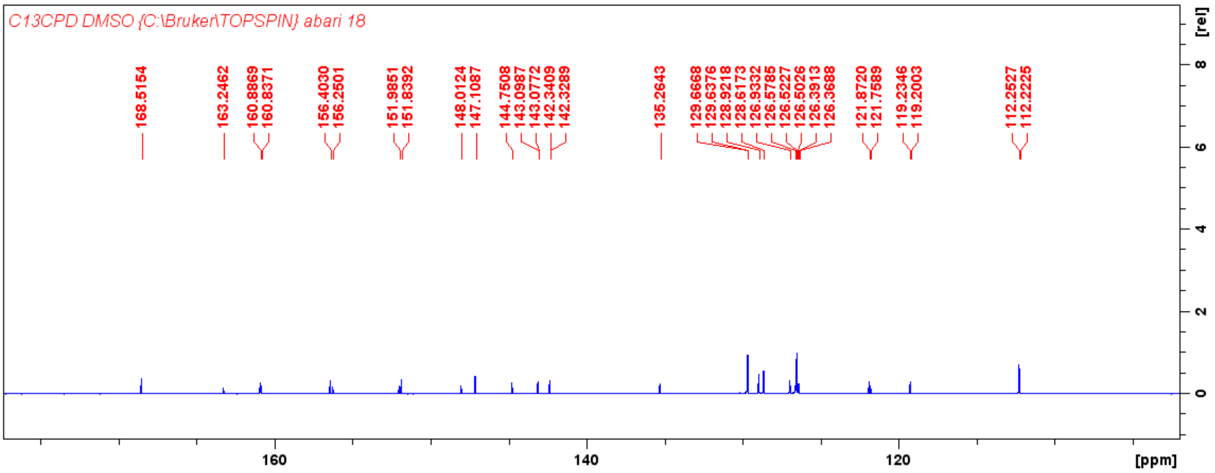

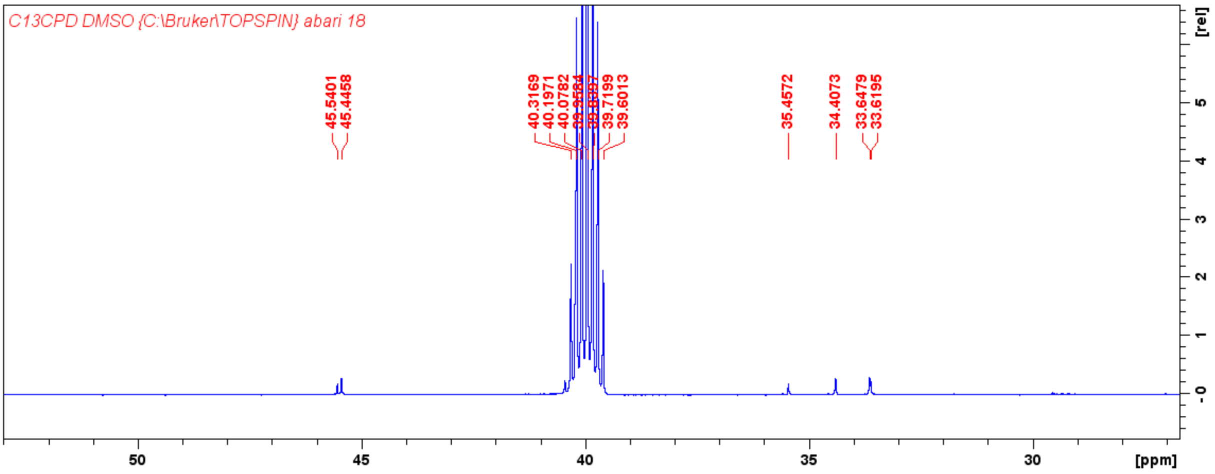

Compound 16 NMR

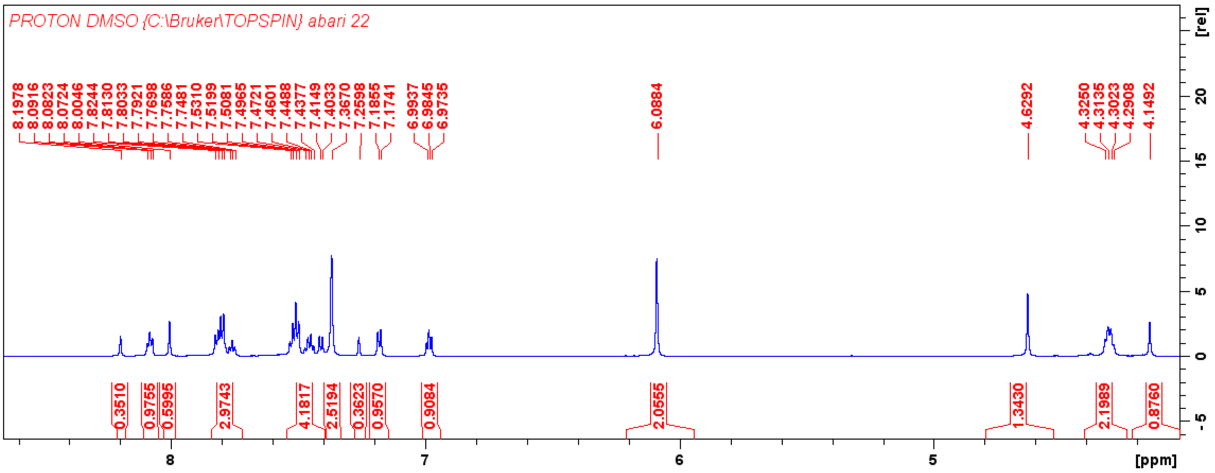

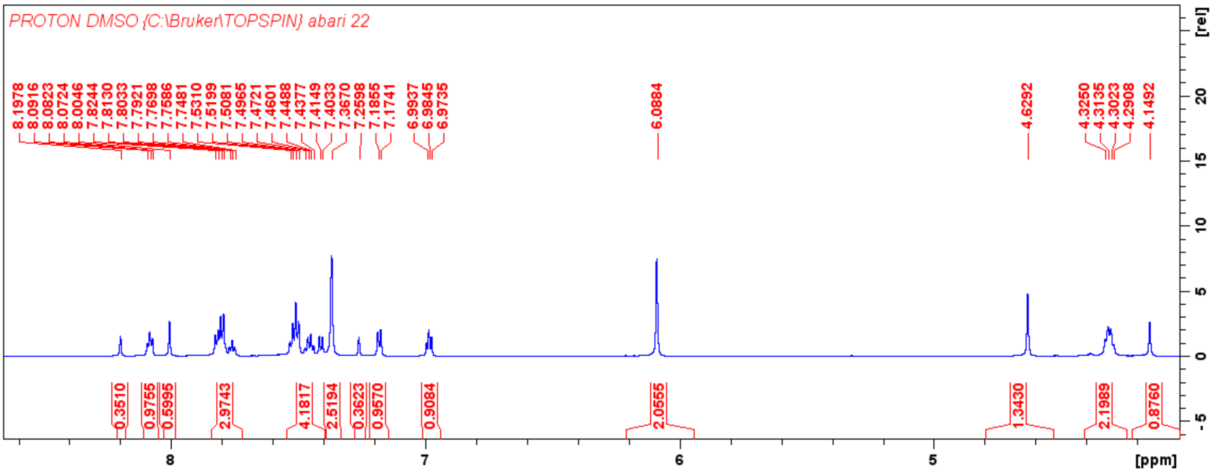

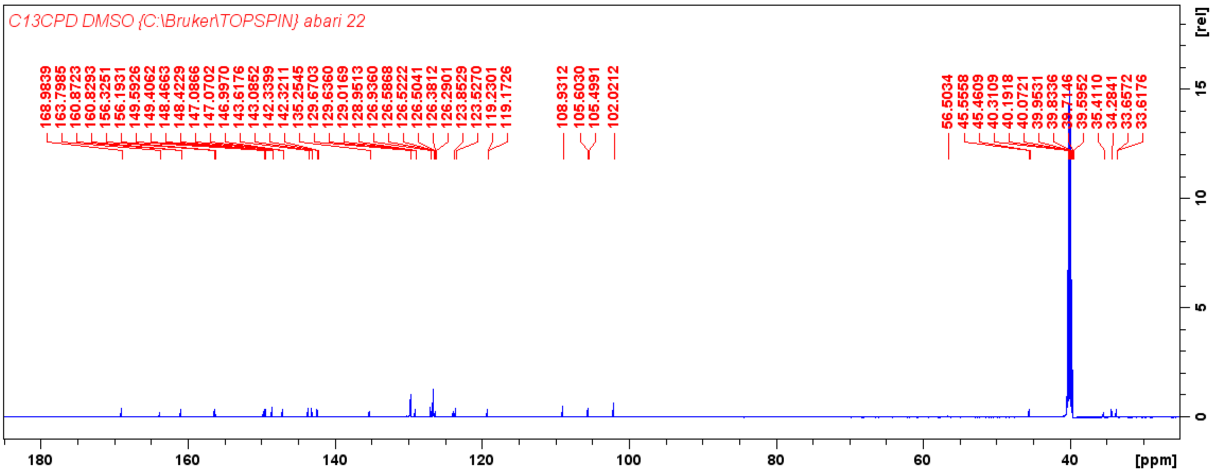

Compound 17 ^{1}H NMR

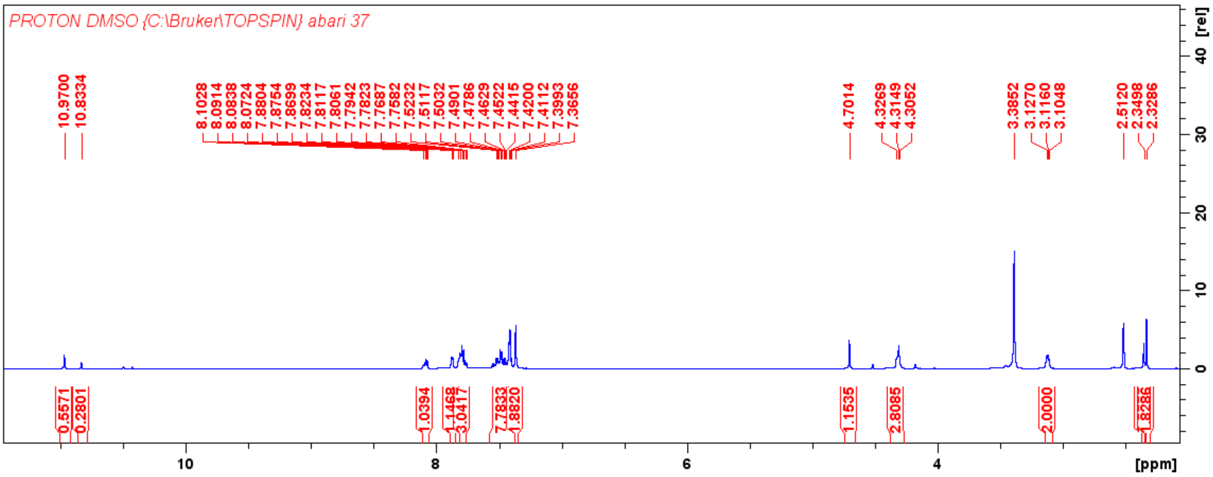

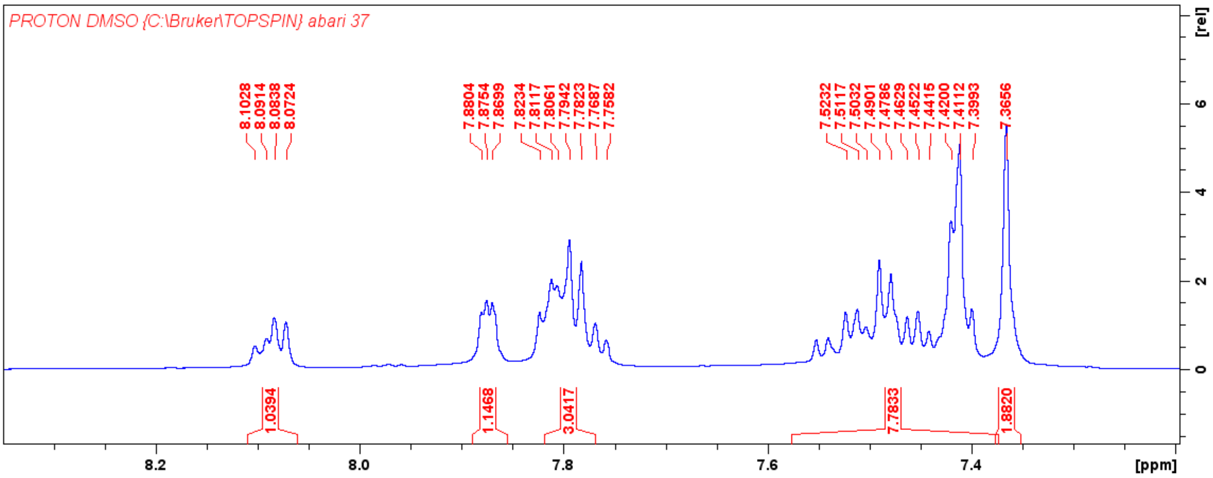

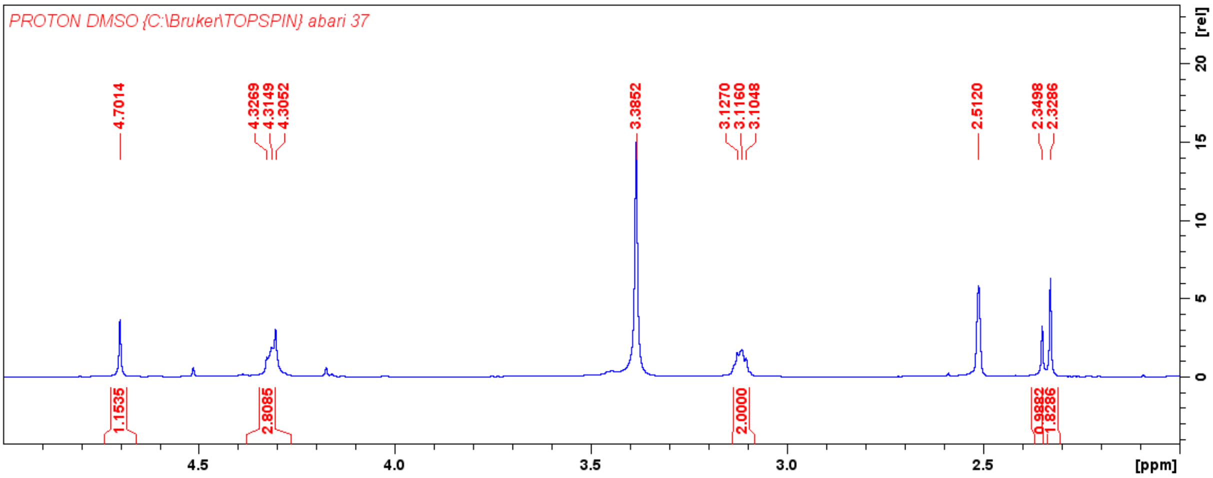

Compound 17 ^{13}C NMR

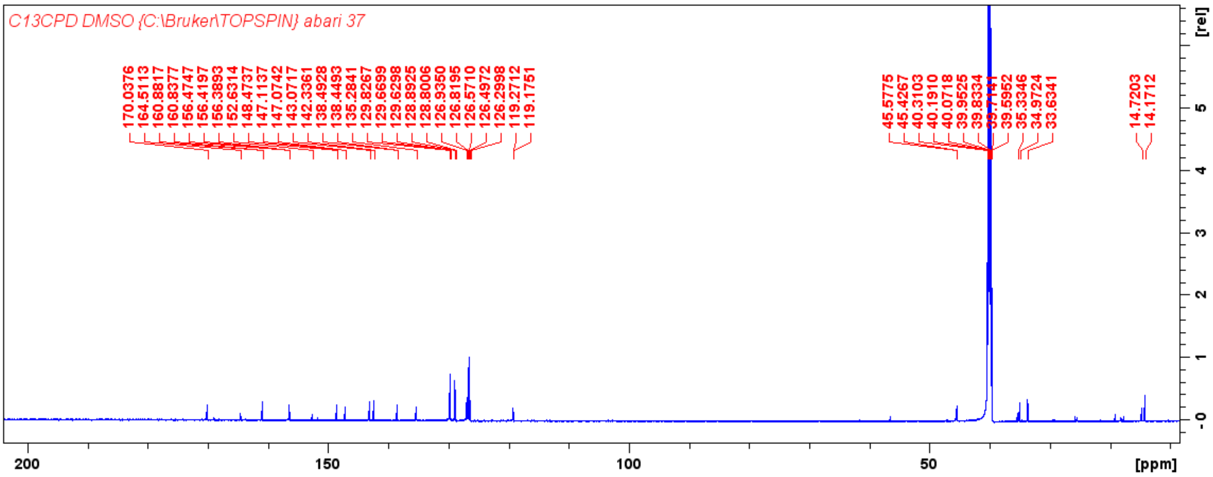

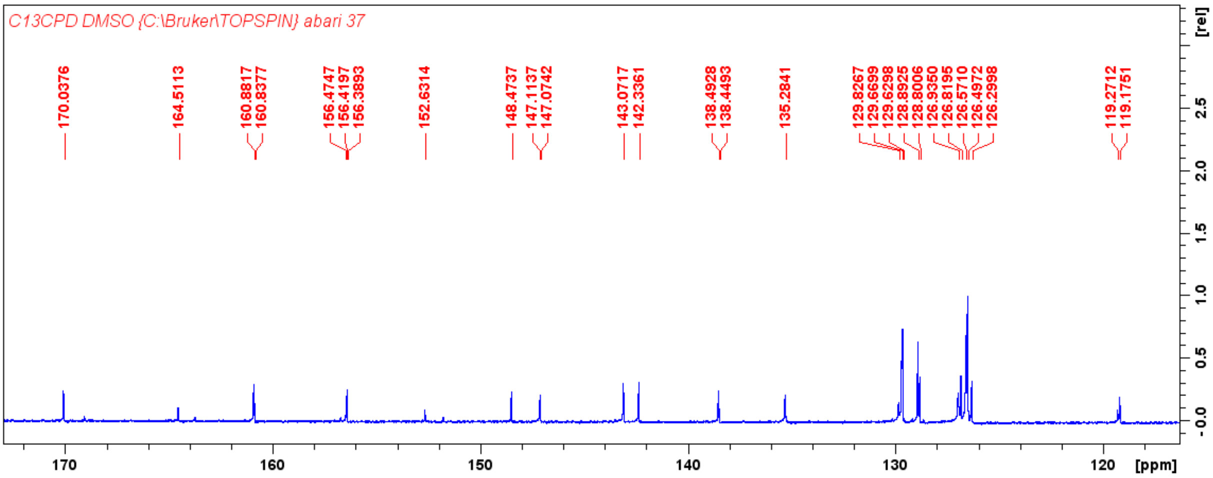

Compound 19 NMR

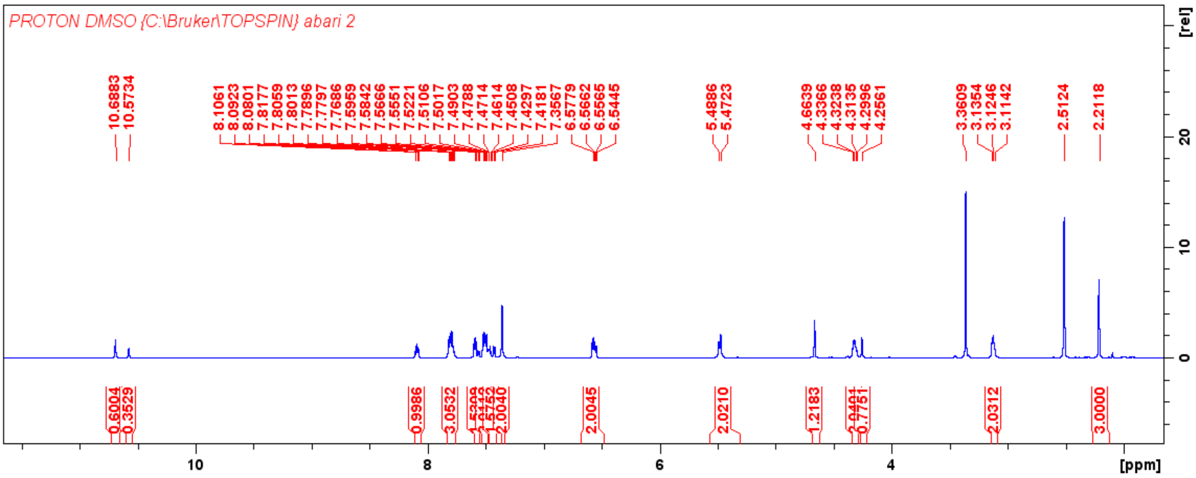

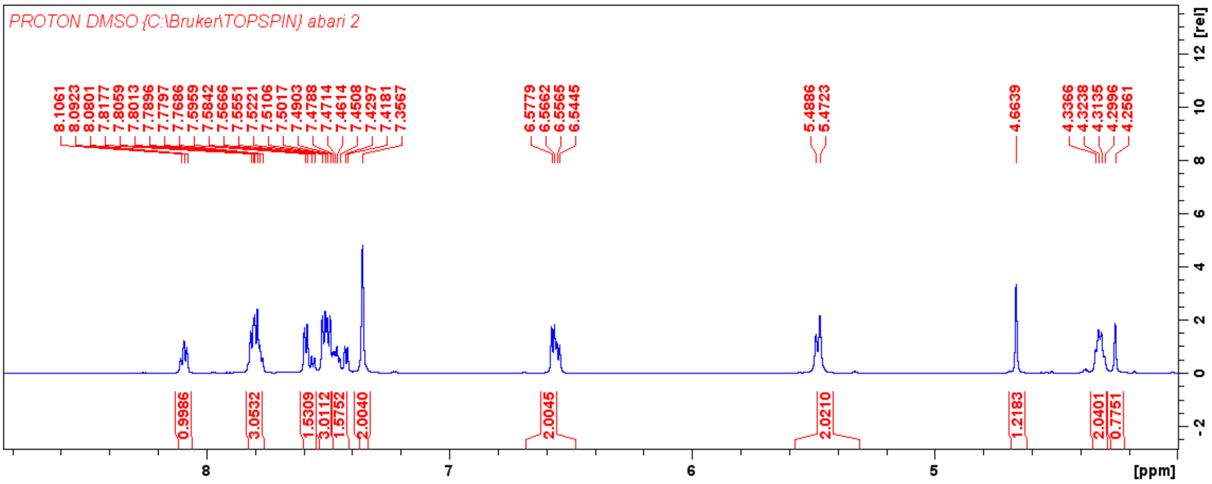

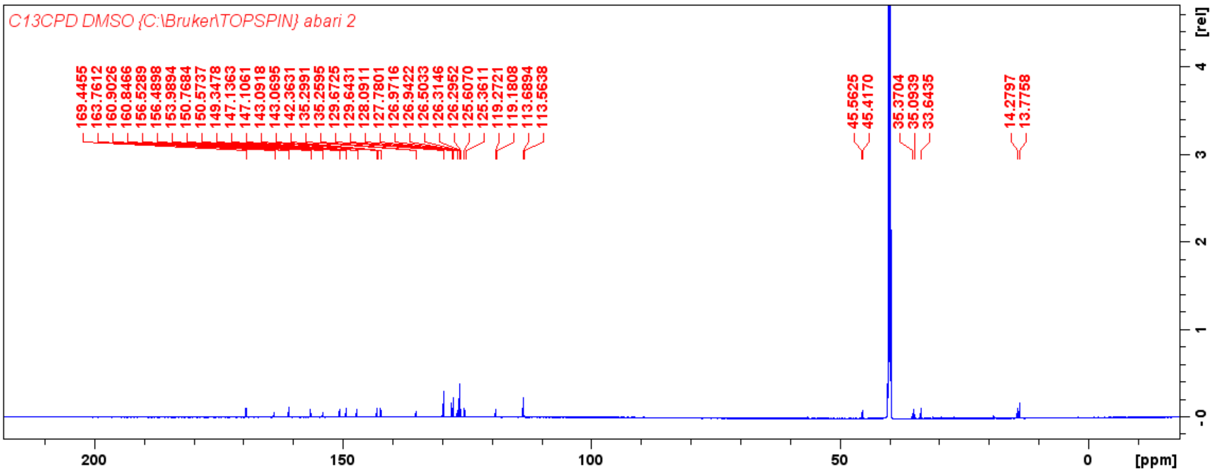

Compound 20 ^{1}H NMR

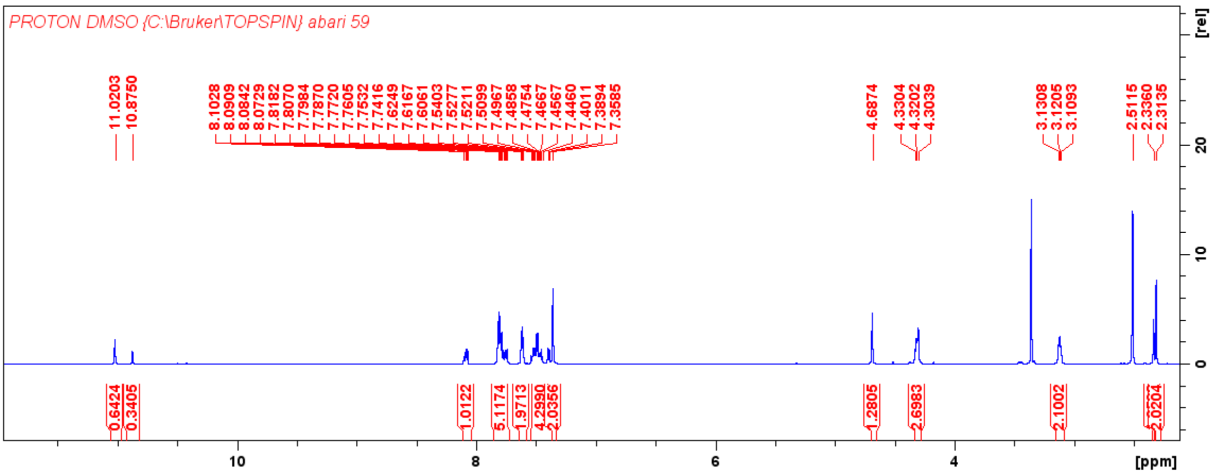

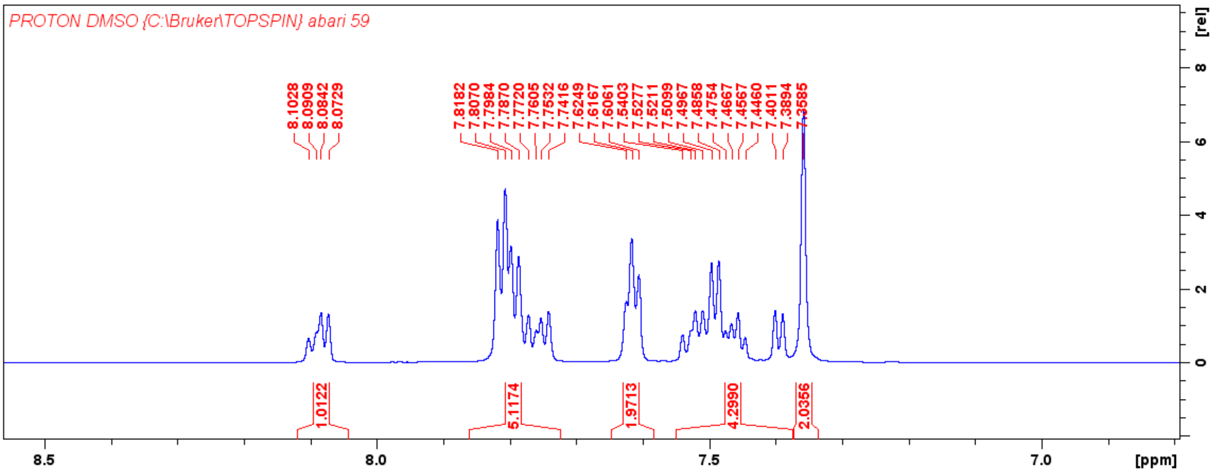

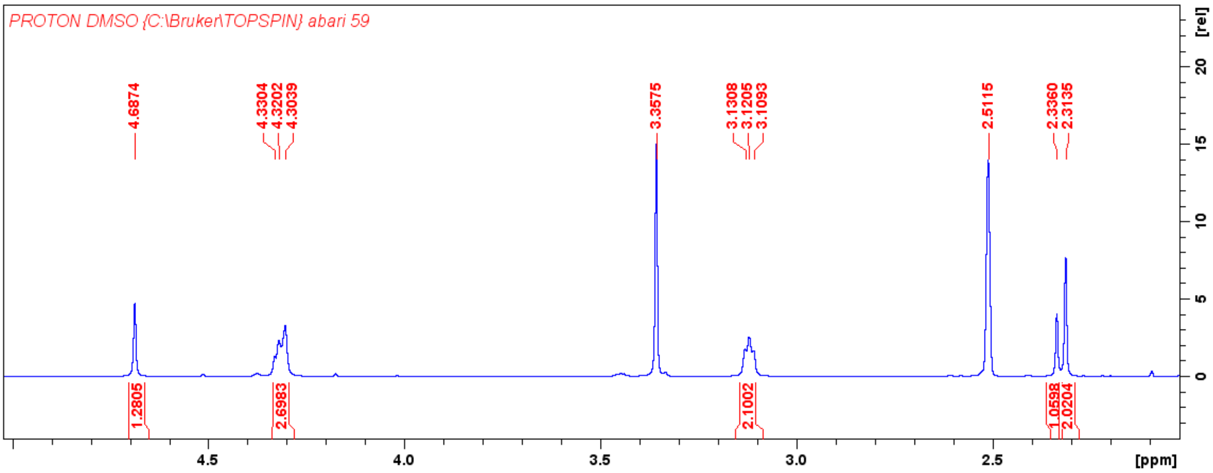

Compound 20 ^{13}C NMR

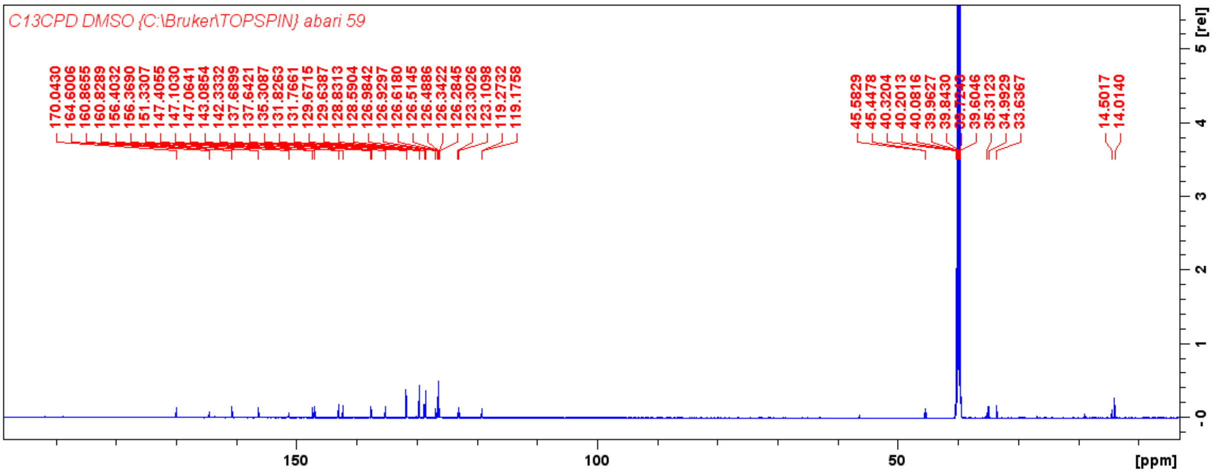

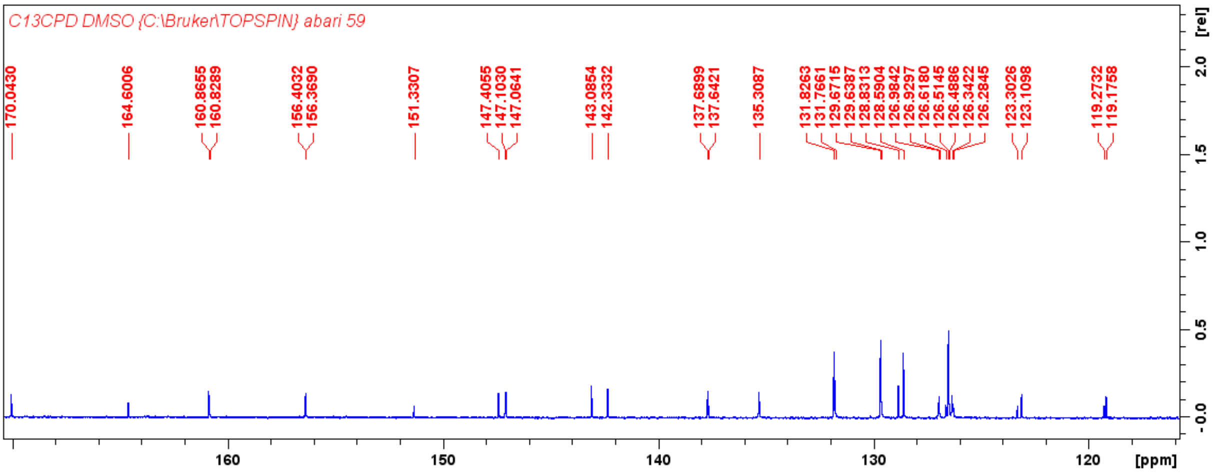

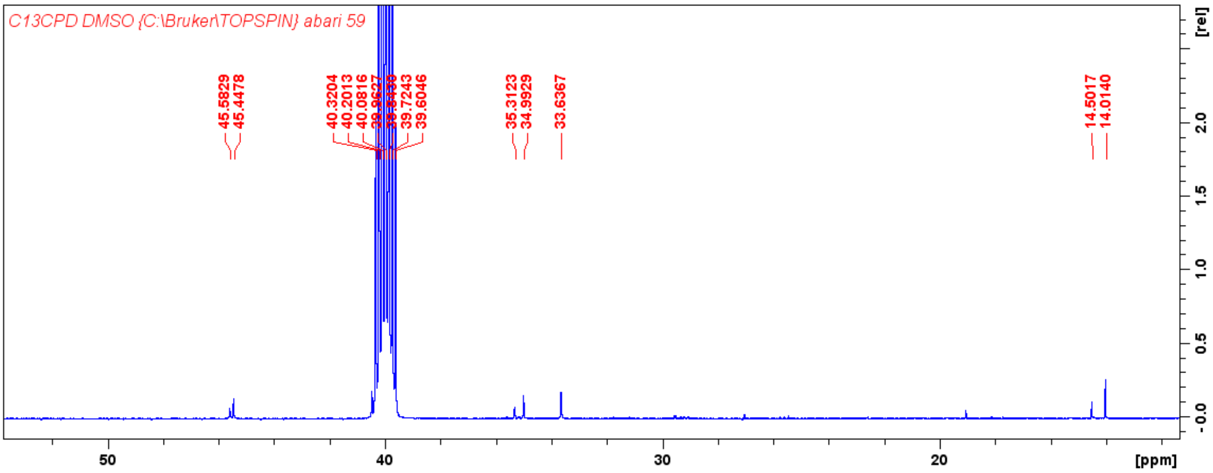

Compound 23 ¹H NMR

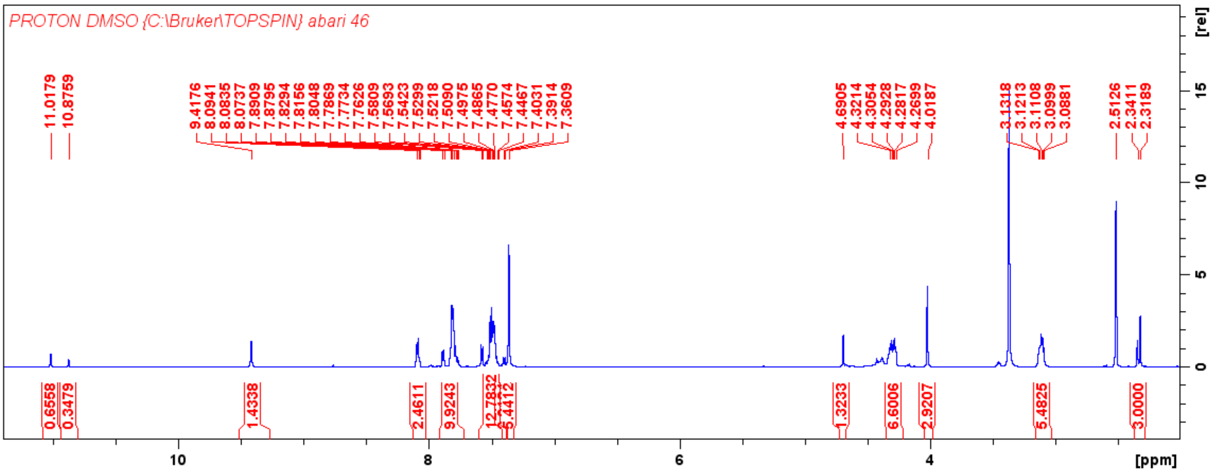

Compound 23 ¹³C NMR

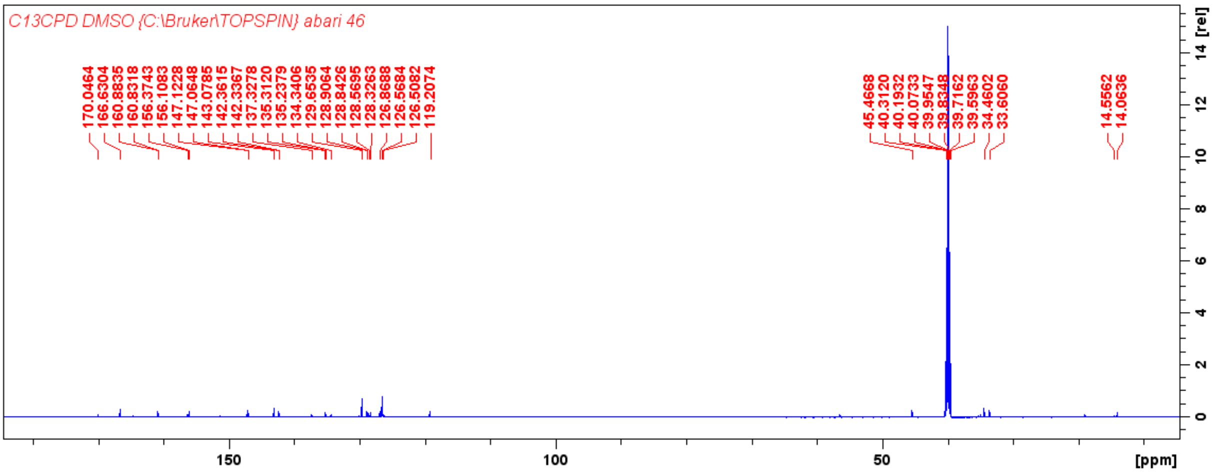

Compound 24 ^{1}H NMR

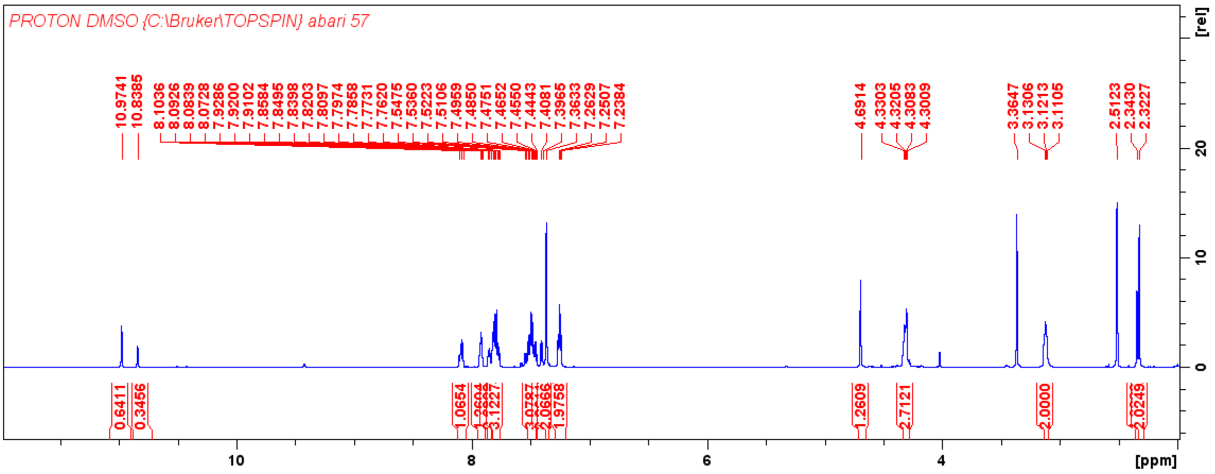

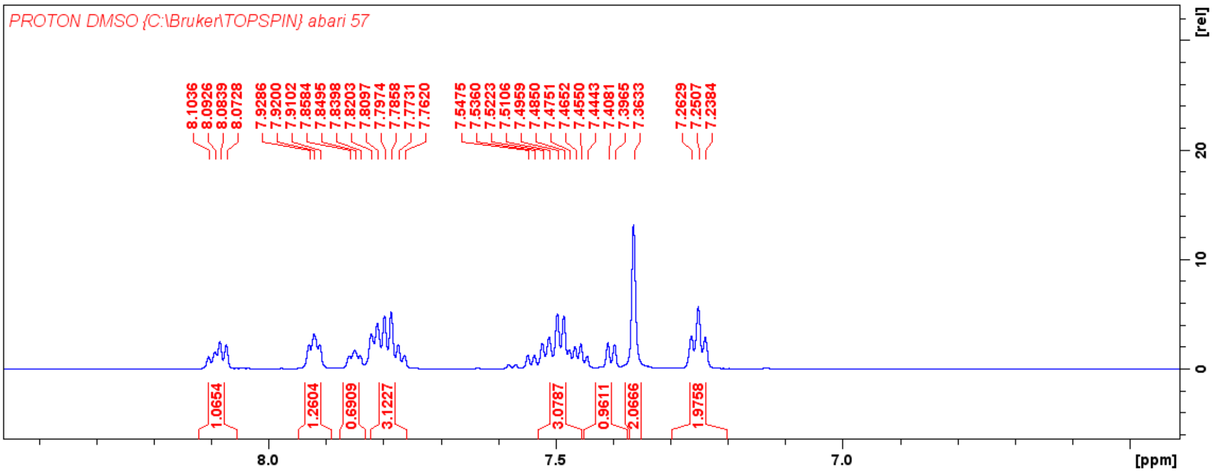

Compound 24 ^{13}C **NMR**

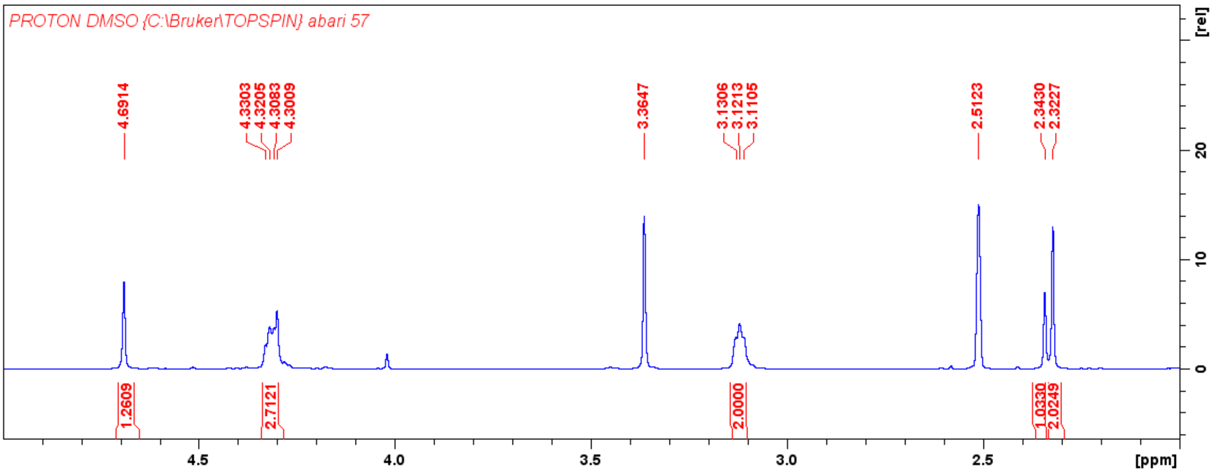

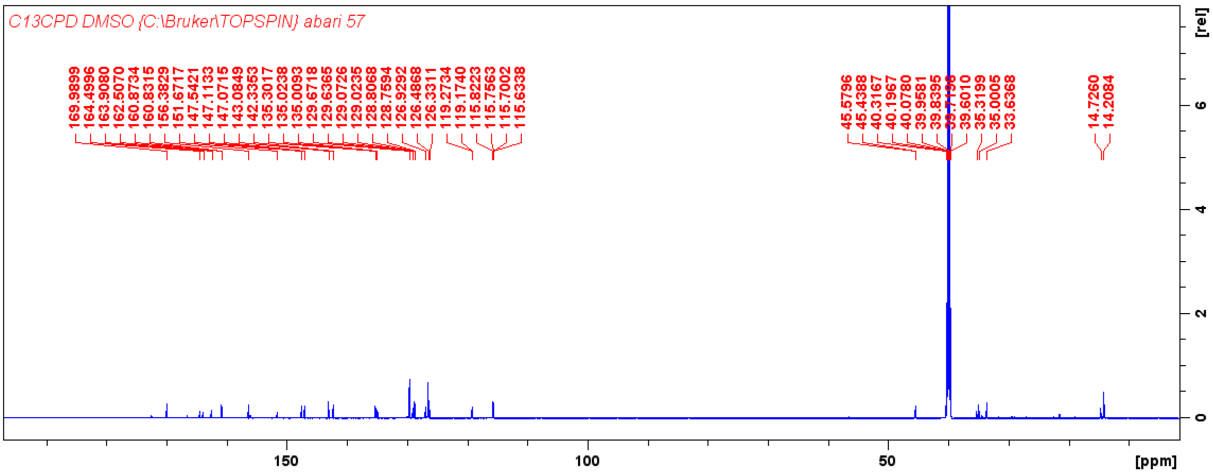

Compound 25 NMR

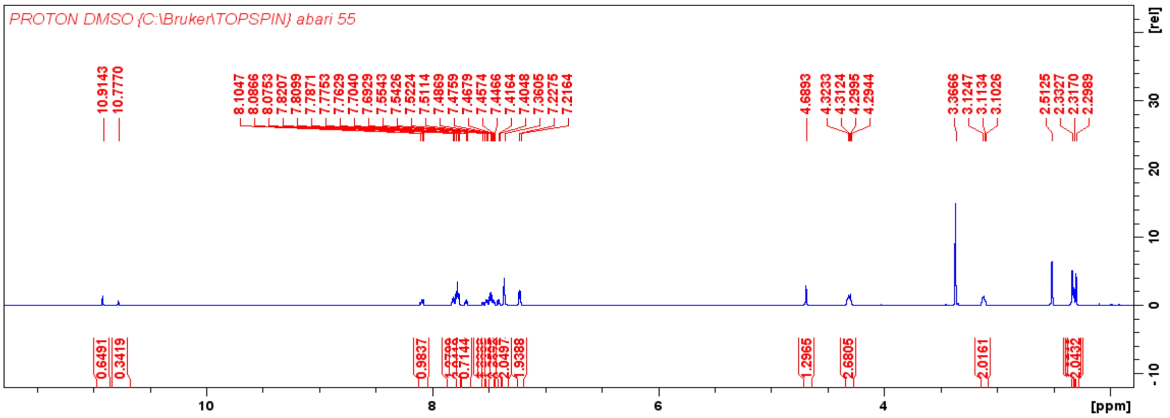

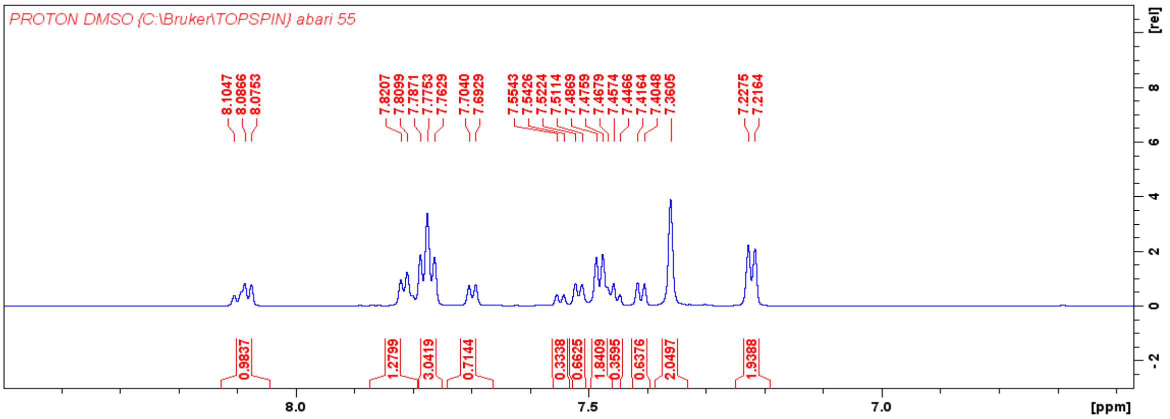

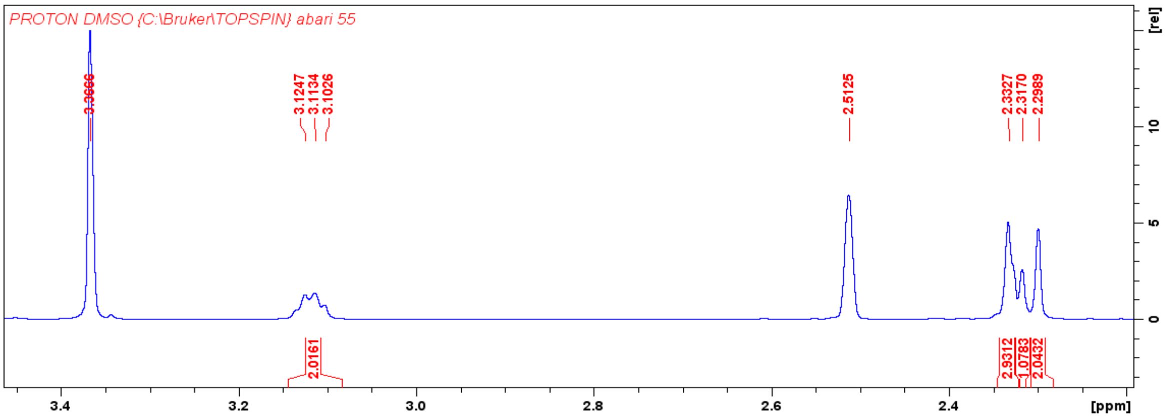

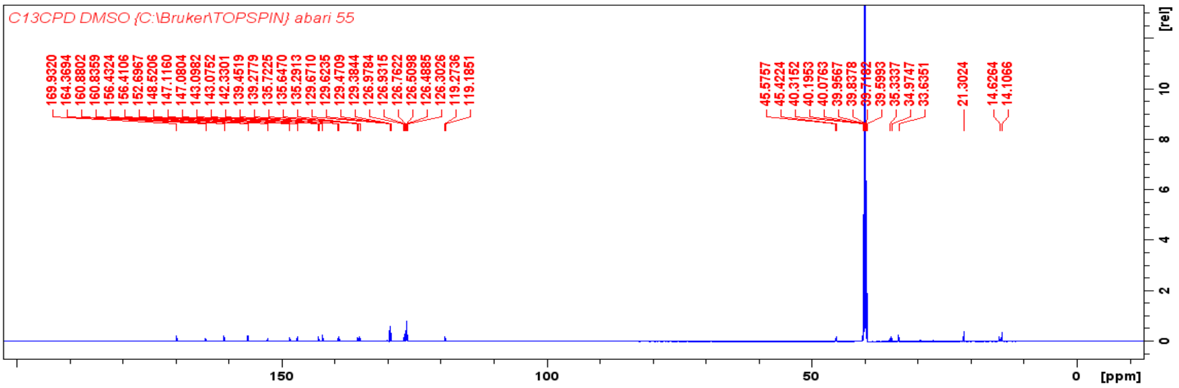

Compound 26 ^{1}H NMR

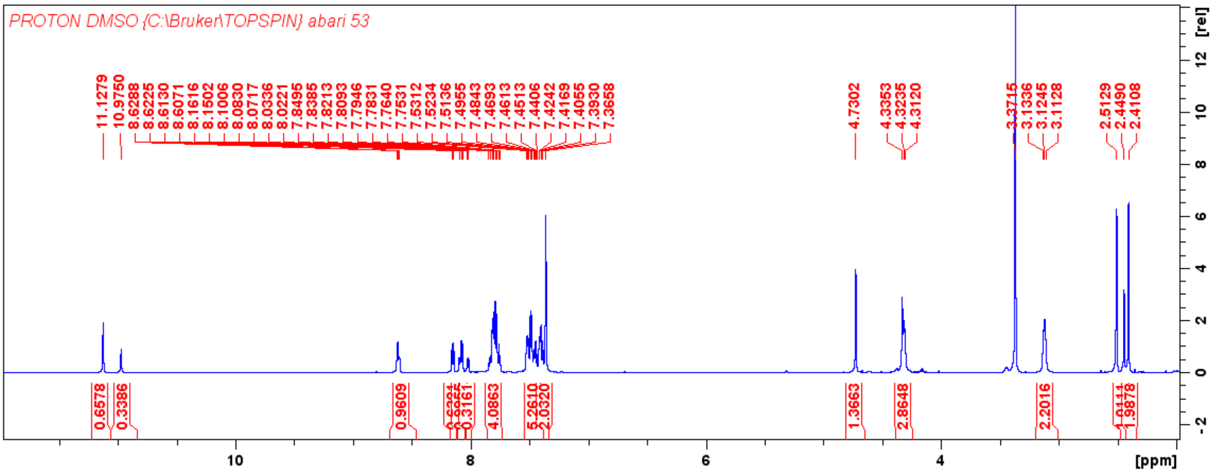

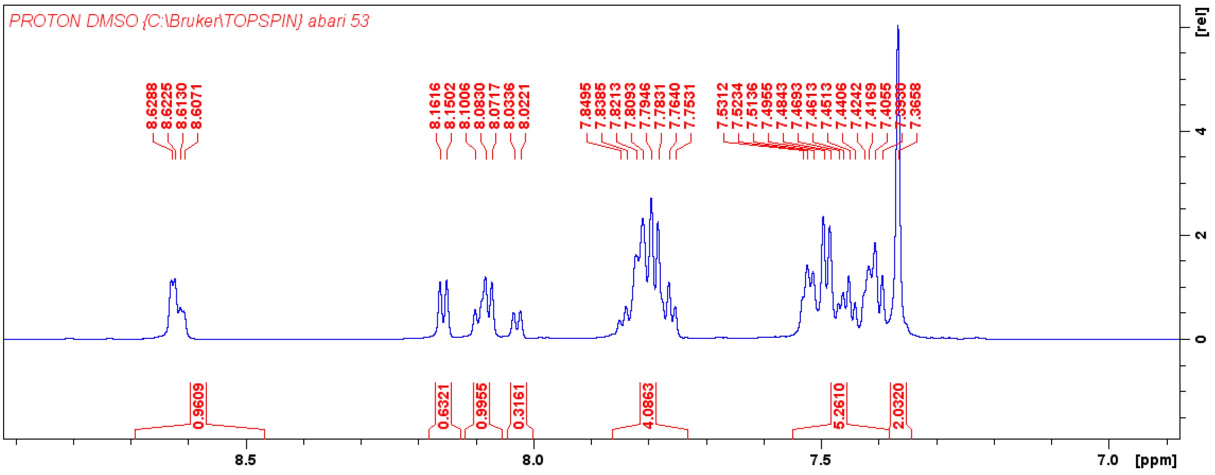

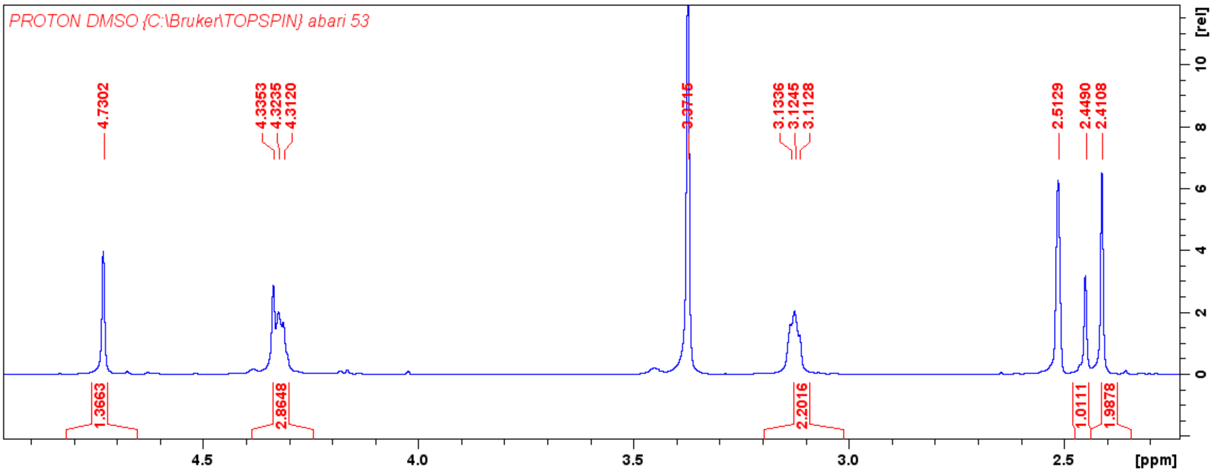

Compound 26 ^{13}C NMR

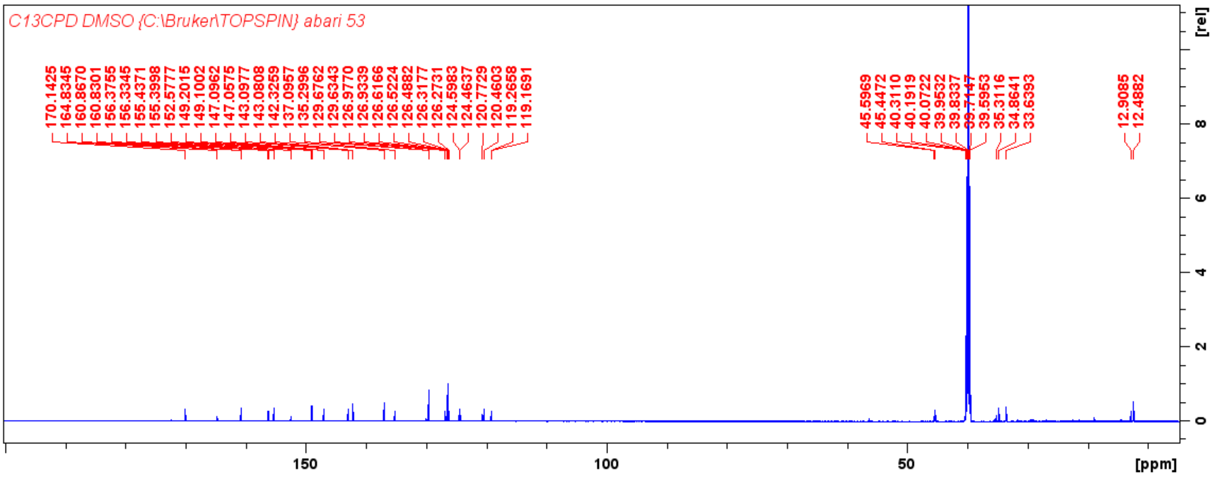

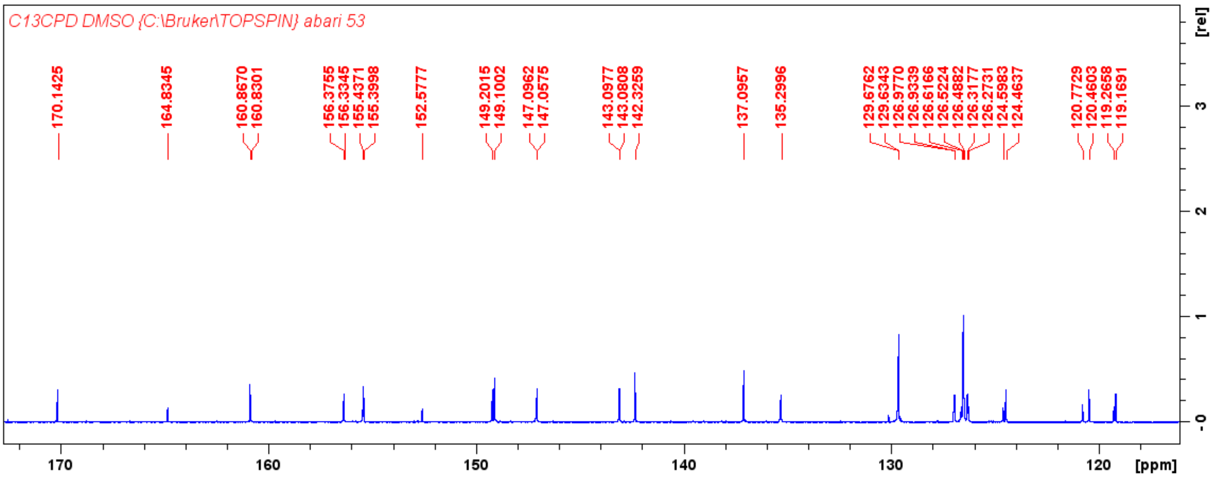

Supplement: Supplementary file 1 [file molecules-27-07703-s001.zip › molecules-1974617-supplementary.pdf]
